# Genome-Wide Analysis of Polyadenylation Events in *Schmidtea mediterranea*

**DOI:** 10.1534/g3.116.031120

**Published:** 2016-08-02

**Authors:** Vairavan Lakshmanan, Dhiru Bansal, Jahnavi Kulkarni, Deepak Poduval, Srikar Krishna, Vidyanand Sasidharan, Praveen Anand, Aswin Seshasayee, Dasaradhi Palakodeti

**Affiliations:** * Institute for Stem Cell Biology and Regenerative Medicine, NCBS campus, GKVK, Bellary Road, Bangalore 560065, Karnataka, India; ** National Centre for Biological Sciences, GKVK Campus, Bangalore 560065, Karnataka, India; † SASTRA University, Thirumalaisamudram, Thanjavur 613401, Tamil Nadu, India; ‡ Manipal University, Madhav Nagar, Near Tiger Circle, Manipal 576104, Karnataka, India; § University of Bergen, Department of Clinical Science, Bergen 5020, Norway

**Keywords:** posttranscriptional regulation, polyadenylation, alternate polyadenylation, 3P-Seq (poly(A)-position profiling), planaria

## Abstract

In eukaryotes, 3′ untranslated regions (UTRs) play important roles in regulating posttranscriptional gene expression. The 3′UTR is defined by regulated cleavage/polyadenylation of the pre-mRNA. The advent of next-generation sequencing technology has now enabled us to identify these events on a genome-wide scale. In this study, we used poly(A)-position profiling by sequencing (3P-Seq) to capture all poly(A) sites across the genome of the freshwater planarian, *Schmidtea mediterranea*, an ideal model system for exploring the process of regeneration and stem cell function. We identified the 3′UTRs for ∼14,000 transcripts and thus improved the existing gene annotations. We found 97 transcripts, which are polyadenylated within an internal exon, resulting in the shrinking of the ORF and loss of a predicted protein domain. Around 40% of the transcripts in planaria were alternatively polyadenylated (ApA), resulting either in an altered 3′UTR or a change in coding sequence. We identified specific ApA transcript isoforms that were subjected to miRNA mediated gene regulation using degradome sequencing. In this study, we also confirmed a tissue-specific expression pattern for alternate polyadenylated transcripts. The insights from this study highlight the potential role of ApA in regulating the gene expression essential for planarian regeneration.

The 3′ untranslated region (3′UTR) governs the stability, localization, and translational efficiency of mRNA within the cell (Barrett *et al.* 2012; Jia *et al.* 2013). Various *cis*-regulatory elements present within the 3′UTR bind to *trans*-acting RNA-binding proteins (RBPs) and small noncoding RNAs to regulate eukaryotic gene expression (Jia *et al.* 2013; Millevoi and Vagner 2010). Hence, 3′UTR processing can have a direct influence on posttranscriptional gene regulation. The generation of a defined 3′UTR involves the cleavage/polyadenylation of pre-mRNA resulting in a stretch of untemplated polyadenosine monophosphate residues [poly(A) tail] of varying length at the 3′ end (Colgan and Manley 1997; Zhao *et al.* 1999). Almost all transcripts in eukaryotes, excluding histone mRNA (Davila Lopez and Samuelsson 2008), are subjected to this process. Furthermore, cleavage/polyadenylation can occur at multiple sites on the same pre-mRNA, leading to different transcript isoforms; this is called alternative polyadenylation (ApA). Often, these ApA events alter the length of the 3′UTRs. In some scenarios, cleavage/polyadenylation events occur within the ORF of the transcript leading to the production of different protein isoforms. Currently, more than 30–50% of genes in higher eukaryotes are estimated to be alternatively polyadenylated (Tian *et al.* 2005; Derti *et al.* 2012). Moreover, the nature of polyadenylated genes and the site of polyadenylation on the mRNA change with the status of the cell. For example, proliferative cells derived upon T-cell activation express transcripts with shortened 3′UTRs (Sandberg *et al.* 2008). Reprogramming of somatic cells to iPS cells also favors transcripts with shorter ApA isoforms (Ji and Tian 2009). Transcripts with longer 3′UTRs are observed during mouse embryonic development, differentiation of monocytes, and in mammalian neurons (Ji *et al.* 2009; Shepard *et al.* 2011). In contrast, the average 3′UTR length was observed to decrease during the developmental stages in *Caenorhabditis elegans* (Mangone *et al.* 2010). Further, ApA was shown to contribute to oncogene activation through the loss of 3′UTR suppressor elements (Mayr and Bartel 2009). Recently, ApA was also implicated in the regulation of membrane protein localization (Berkovits and Mayr 2015).

The planarian *Schmidtea mediterranea* serves as an ideal model system to study stem cell function and regeneration (Oviedo *et al.* 2008). The unique property of planaria to regenerate any organ, including the brain, is attributed to the presence of adult pluripotent somatic stem cells called neoblasts (Sanchez Alvarado 2003). Various genes and gene regulatory mechanisms have been implicated in neoblast function (Rossi *et al.* 2008; Pearson and Sanchez Alvarado 2010; Onal *et al.* 2012; Hubert *et al.* 2013; Sasidharan *et al.* 2013; Solana *et al.* 2013; Zeng *et al.* 2013). Recent efforts have identified several RNA binding proteins, mostly involved in translation regulation and mRNA stability, that are enriched in neoblasts. These were shown to be essential for stem cell maintenance and differentiation (Rouhana *et al.* 2014, 2010).

At present, the study of posttranscriptional gene regulation in the planarian model system is limited by the lack of well-annotated 3′UTRs. In order to address this issue, we carried out poly(A) position profiling (Jan *et al.* 2011) on the transcriptome of *S. mediterranea*. Using the 31,377 poly(A) sites that were identified, we could reliably annotate the 3′UTRs for 44% of the protein coding genes. Around 40% of the annotated transcripts were associated with more than one poly(A) site, hinting at the predominant role played by ApA in regulating gene expression. We identified around 97 transcripts in planaria that are polyadenylated within their ORFs. Furthermore, we explored the functional association between miRNA-mediated gene regulation and ApA, which revealed that miRNA binding sites are either clustered around the proximal or distal polyadenylation sites. We confirmed tissue-specific expression of 3′UTR isoforms arising from alternate polyadenylation. Thus, the work presented here highlights the potential role played by ApA in planarian regeneration. This study will also help to identify the regulators essential for the ApA mechanism in planarians.

## Materials and Methods

### Identification of polyadenylation machinery in planaria

We used a polyadenylation machinery protein sequence from humans as a query and blasted it against the PlanMine database to determine the corresponding homologous in planaria (Brandl *et al.* 2015). For 12 of the proteins (CFIm25, CPSF100, CPSF30, CPSF160, CPSF73, CstF50, CstF77, CstF64, ClpI, PAPOA, PAPOB, and PAPOC) the homolog could be easily determined. The corresponding IDs from the Ox_Smed_V1 version of the transcriptome could be identified and the presence of these were experimentally verified through RT-PCR. For the four proteins from the polyadenylation machinery (CFIm59, CFIm68, hFIP1, and Pcf11), the sequence search through the PlanMine database did not result in a reliable hit. To rule out the possibility that these could be missed due to poor genome assembly/annotation, we searched for similar sequences in the recent version of the Smed genome (Robb *et al.* 2015). The query through BLAST resulted in hits that had partial similarity to the human homologs. All the orthologous sequences for these proteins were downloaded from the gene tree available at the Ensemble database. The gene tree was reconstructed by including the hits from the Smed genome along with orthologs from other organisms using *ete3* (Huerta-Cepas *et al.* 2010). The percentage identity matrix (PIM) was also constructed through multiple sequence alignment using clustal *omega* (Sievers *et al.* 2011). Both the constructed gene tree and the PIM confirmed the sequence divergence of these four proteins in planaria (Supplemental Material, Figure S1, Figure S2, Figure S3, and Figure S4).

### Preparation of 3P-Seq library

The 3P libraries were prepared as per the standard protocol previously described in the literature (Jan *et al.* 2011) with some modifications. The total RNA amounting to 200 μg was extracted using the trizol method from both sexual and asexual planaria, *S. mediterranea*, to prepare 3P libraries. The extracted RNA was subjected to RNase H treatment for 25 min. The cDNA was prepared from the library using Superscript III RT (Catalog no. 18080044) and the derived product was directly amplified using PCR (25 cycles) without desalting. The adaptor (Illumina genomic sequencing adapter) ligated product was sequenced using an Illumina GAIIx platform.

### Analysis of 3P-Seq reads

In order to determine the polyadenylation sites in *S. mediterranea*, poly(A) RNAs were isolated from the whole organism (both sexual and asexual forms separately) and then subjected to 3P-Seq. Around 165 million and 103 million reads were obtained for the sexual and asexual forms, respectively. These reads were combined, processed, and mapped to the SxlV3.1 genome using bowtie (Langmead *et al.* 2009). The raw reads obtained from the 3P-Seq protocol (Jan *et al.* 2011) were reverse complemented. The reads ending with a poly(A) stretch were trimmed down to contain a maximum of two A’s at the end. These were then selectively mapped onto the genome and the reads were considered as 3P-tags if: a) the alignment ended with the mismatch in the 3′, end as it would signify the untemplated adenosine nucleotide added by poly(A) machinery; and b) the minimum length of read was above 20 nucleotides (nt). Raw numbers are shown in Figure S5. The reads were aligned to the *S. mediterranea* genome using bowtie with –m 4 and –v 2 options. Around 77 million reads aligned to the genome, among which 34 million reads qualified as 3P-tags. The density profile of 3P-tags across the whole genome revealed that 81% of the genomic locus is covered with a minimum depth of three 3P-Seq reads (Figure S5). Hence, we used a minimum cut-off of three 3P-tags to identify the 3P-peaks. The 3P-peak has a minimum coverage of three 3P-tags associated across the complete length. This stringent scan across the entire genome involving the above cut-offs, resulted in 31,377 unique polyadenylation sites. The identified cleavage sites have a median length of 50 nt (Figure S5) and were highly AT-rich (Figure S5). The in-house python scripts used to process the raw FASTQ data can be obtained from github (https://github.com/VairavanL/3PSeq_analysis).

### Ion torrent sequencing

The total RNA, amounting to 7 μg extracted from both the sexual and asexual worms, was subjected to cDNA conversion using OligodT primers from an AffinityScript QPCR cDNA Synthesis Kit. The cDNA was fragmented using Covaris. The fragmented DNA was quantified and subsequently utilized for library preparation. End-repair and adapter ligation was carried out according to the protocol (Ion plus fragment library kit, # 4471252). The samples were bar-coded at this step and then cleaned using Ampure XP beads. The library was amplified using eight cycles of PCR for enrichment of adapter-ligated fragments. The amplified product was again cleaned using Ampure XP beads. The prepared library was quantified using Qubit and validated for quality by running an aliquot on a High Sensitivity Bioanalyzer Chip (Agilent). Ion PGM sequencing was performed on this sample. Around 2.3 million reads were derived from the transcriptome through ion torrent sequencing after poly(A) pulldown. Approximately 70% of these sequenced reads were > 50 nt in length (Figure S12). The reads containing poly(A/T) (28.6%) were selected as these would represent the polyadenylated mRNAs. The selected reads were subsequently trimmed to remove poly(A). We used blast (Altschul *et al.* 1990) and bowtie to align the trimmed reads to the genome. Around 0.37 million reads were successfully aligned and these covered 18,834 of 31,377 3P-peaks.

### Scanning for poly(A) signals (PAS)

In order to identify a statistically significant hexamer that is enriched across the identified 31,377 peaks, we adopted a pipeline depicted in Figure S6. A random dataset was generated by shuffling the 3P-peak sequences using the Knuth-shuffle algorithm. The probability of hexamer occurrences [4096 ($4^{6}$)] is calculated across all positions in both shuffled and unshuffled sequence datasets. The z-score is calculated from the occurrence value for each position. The z-scores are then converted into *P*-values using the *pnorm* function in R and the *P*-value is adjusted using Bonferroni corrections. A particular signal was considered as an enriched hexamer motif at a specific position if it had an adjusted *P*-value < 0.01 and at least a twofold enriched occurrence compared to a random sequence. We identified 737 enriched hexamer signals across various positions in the peak. These 737 hexamers are position-dependent enriched motifs. Among these, the canonical signal 5‘-AAUAAA-3’ was present in 14,183 peaks. A randomized dataset of 3P-peaks was generated to test for the significance of these hexamers and the whole pipeline was repeated on this dataset. We did not find any significant hexamer enrichment. This shows the robustness of our methodology. We also looked for pentamer, heptamer, and octamer sequence enrichment in the identified peaks. We did not find any enriched motifs of these sizes.

To identify other signals (minor) apart from 5‘-AAUAAA-3’ that could also act as PAS, we adopted a method as illustrated in Figure S6. We performed Fisher’s exact test to identify minor signals. Total peaks were divided into two categories: one that housed the canonical PAS (14,183 peaks) and another that did not contain canonical PAS (17,194 peaks). We considered 736 signals (apart from the canonical signal) that were enriched and performed a two-category association test to determine in which category they were enriched. Based on the association value (*i.e.*, odds ratio < 0.75 and *P*-value < 0.01), 82 signals were identified that could likely represent the minor signals. In order to test the robustness of this protocol, the whole analysis was also repeated with different cut-offs of 3P-tags (ranging from four 3P-tags up to 100 3P-tags). Although the number of 3P-peaks reduced with the increase in cut-off, the positional enrichment of the major PAS signal, along with the secondary (∼58% retained) and the minor signal (∼44% retained), did not change (Figure S7).

A directed search was also performed to identify the PAS associated with each of 31,377 3P-peaks by scanning the sequence within the distance of 10–40 nt from the cleavage site (3′ end of the sequence). The first priority was given to the canonical signal 5‘-AAUAAA-3’, and around 40.8% peaks were found to house this signal within 40 nt from the cleavage site. The second priority was given to all hexameric sequences that had just one mismatch to the canonical signal 5‘-AAUAAA-3’. The one closest to the cleavage site was considered as PAS for the peak. Additionally, of the remaining peaks, around 40.4% could now be associated with PAS containing a variant of the canonical signal with one mismatch. Similarly, the same process was repeated with PAS containing two mismatches. Finally, around 97.5% (30,994) of the polyadenylation sites were assigned with a PAS signal (Figure S6).

### Identification of hexameric motifs using MEME

We used MEME 4.10.2 (Bailey *et al.* 2006) to predict PAS along with two other strategies. We used 3′UTR sequences from zebrafish, *Drosophila*, humans, *C. elegans*, and mouse as positive background datasets for MEME. We categorized the identified polyadenylation sites into three bins based on its coverage as top, median, and last 1000 sites. When we individually ran MEME on these three datasets, we identified 5‘-AAUAAA-3’ as a major enriched signal. Along with 5‘-AAUAAA-3’, we got other variant signals, which overlapped with 737 signals that we identified before (Figure S6).

### Transcript and peak statistics

There are 43,209 contigs in the smed genome (Version 3.1). In total, 31,067 maker transcripts are spread across 16,053 contigs. Among these, 7219 contigs (9449 maker transcripts) have only maker-annotated transcripts and no identified 3P-peaks falls into these contigs. On the contrary, there are 3793 contigs (5988 3P-peaks) that have only 3P-peaks but no annotated transcripts that fall within them. There are 8834 contigs that have both annotated transcripts and 3P-peaks associated with them. These 8834 contigs constitute 21,577 transcripts and 29,134 3P-peaks. Similarly, in the Ox_Smed_v1 transcriptome, 2432 contigs (3175 transcripts) have only annotated transcripts. 5145 contigs (7916 3P-peaks) have only peaks associated without containing any transcripts. In the dd_Smed_v4 transcriptome, 3888 contigs (6459 transcripts) have only transcripts and 4341 contigs (6566 3P-peaks) have only 3P-peaks coming from them.

### Stitching of transcripts

We could classify the association of polyadenylation sites to the transcript into five different categories. Polyadenylation sites that falls inside the transcript were excluded from this analysis, except for the ones that are present in the last exon and end after the annotated transcript model. The transcripts that have a 3P-peak outside the annotated end were extended to get the complete gene model by utilizing the genomic information (Figure S9). We used four datasets (Genscan, Genpred, Cufflinks from RNA-seq, and the EST database) to fill the gap between known transcript model ends and the polyadenylation site start. At least two of the four datasets should support a region to extend the existing transcript model. If a transcript is associated with more than one polyadenylation site, we considered it as a different 3′UTR isoform and stitched each polyadenylation site individually. For comparison of the 3′UTR lengths across different organisms, we derived the information from biomart (Ensemble Genes 84 version). The longest transcript isoform was chosen for each gene to calculate the 3′UTR length.

### Using RNA-seq data as evidence for stitch and validation of cleavage site

We considered those transcripts that have one cleavage site and filtered them by genomic length. A cut-off of 1 kb between transcript end and peak start was used. Transcripts that satisfied these two conditions were taken for analysis. We divided these transcripts into two bins. One was the Upstream bin, which had sequences corresponding to the region between the transcript end and peak start. We also took exactly the same genomic length sequence as the Upstream bin after the cleavage site and called it the Downstream bin. We made sure that there was no transcript that started in the Downstream bin. To calculate RNA-seq coverage we used the following formula.

Coverage=(((Number of reads aligned/2)∗Read length)/(Transcript length))

The total numbers of reads are reduced by half as it represents paired-end data. Coverage is normalized to the total length of the transcript.

### Validation of the candidates through RT-PCR

The forward primers were designed from the region within the annotated transcripts and the reverse primers were designed from the region spanning the derived 3P-peak. Candidates were chosen based on the strength of the 3P-peaks (number of 3P-tags within the 3P-peak region). Candidates having a similar number of 3P-tags on both the proximal and distal sites were selected. As the 3P-Seq was performed from the RNA isolated from the whole organism, this would ensure that both the isoforms of the transcripts are expressed. These were further filtered to ensure that the distance between the proximal and distal peaks was ∼2 kb. 1 μg of RNA was converted to cDNA using SSII RT (Invitrogen). The PCR primers for cDNA were designed as shown in Figure 4E (Table S9). The RT-PCR was performed using LA Taq (TAKARA). Amplicons were run on agarose gel and subsequently sequenced.

### Degradome sequencing

We adopted parallel analysis of RNA ends (PARE), a specific sequencing method, to identify degraded RNA products (German *et al.* 2008; Karginov *et al.* 2010; Cass *et al.* 2016). These degraded RNAs are the by-products of AGO-DROSHA processing. Total RNA was isolated from sexual and asexual strains of *S. mediterranea*. Around 500 ng of RNA was directly adapter ligated using T4 RNA ligase, to select only for degraded mRNA that would have a free 5′ phosphate. The ligated RNA was then reverse transcribed using oligo dT (with an attached adapter sequence) and PCR amplified using primers from the TruSeq Illumina Small RNA library. The library was purified using Ampure XP beads and sequencing was performed on an Illumina HiSeq-1000. We got 61 and 72 million reads from the sexual and asexual strains, respectively. These reads were adapter trimmed and the first 15 nt of the reads were used for alignment to the genome and transcriptome using bowtie allowing no mismatches. To find miRNA correspondence for each tag, we used the FASTA v36 program using the parameters -n -H -Q -f -16 -r +15/-10 -g -10 -w 100 -W 25 -E 100 -i –U and scored the alignments using a match-mismatch profile in the seed and guide region. The analysis pipeline used here has been published previously in the literature (Moran *et al.* 2014). In metazoans, the requirement for complementarity between the miRNA and its corresponding target for AGO cleavage is still debatable. The previously published literature hints at the requirement of complementarity at the ninth, 10th, and 11th nucleotides, along with the seed region for AGO-mediated mRNA degradation (Shin *et al.* 2010; Elkayam *et al.* 2012; Addo-Quaye *et al.* 2009). Hence, we selected for sequence complementarity between the degradome tag and the miRNA, even at the ninth, 10th, and 11th positions. This would ensure that the derived degradome tags were a result of AGO cleavage. We then specifically analyzed these degradome tags based upon their location on the proximal or distal 3′UTR region.

### miRNA predictions using miRanda

All of the transcripts that had two cleavage sites associated with them were chosen for prediction of miRNA targets. Transcripts that had both the cleavage sites within them were not considered further for the analysis. We extracted the sequences corresponding to the proximal and distal UTRs from the transcripts. These sequences were specifically scanned for miRNA binding sites using a tool called miRanda (John *et al.* 2004). We downloaded all the known miRNAs of *S. mediterranea* from miRbase (Griffiths-Jones *et al.* 2008). The predicted binding sites from miRanda were filtered based on the complementarity score (score ≥ 140) calculated from an algorithm, as well as the free energy of optimal complementary base pair interaction between the 3′UTR and miRNA sequence (Δ G ≤ −14Kcal/mol). These filtered binding sites were normalized to the length of the 3′UTR region.

### Control dataset for distribution of miRNA binding sites

A randomized dataset was generated to determine the statistical significance for bimodal distribution of miRNA binding sites in transcripts with two 3P-peak candidates. The dataset was generated by randomizing the association of a second 3P-peak to single 3P-peak-containing transcripts. Around 1550 (same size as the actual dataset) single 3P-peaks were also randomly chosen 100 times. In order to capture the native distance distribution between two 3P-peaks, only the distal 3P-peak from two 3P-peak candidates were randomly chosen, along with the region between the peaks and stitched to single 3P-peak candidates. We predicted miRNA binding sites and normalized the dataset as described above. We performed Hartigan’s dip test for multi-modality for the randomized 100 datasets to see how many of the randomized datasets followed a multi-modal distribution as an actual dataset. Significant *P*-values and a higher Dip score suggested multimodal distribution. Randomized datasets with *P*-values < 0.05 were considered to be multimodal (Figure S14).

### Data availability

The data generated in this study has been deposited in the National Center for Biotechnology Information Sequence read archive (SRA) database. Accession ID: SRP070102. All the NGS data used in this study can also be visualized through JBrowse applet (Westesson *et al.* 2013) at http://bugbears.ncbs.res.in/planaria3p.

## Results

### Identification of polyadenylation machinery in planaria

The process of polyadenylation involves two major reactions. The first reaction is the endonucleolytic cleavage of pre-mRNA at a specific site. This is followed by the polymerase reaction that adds the polyadenylated tail to the cleaved product. These reactions require various protein factors that are specifically guided to the cleavage site, dictated by the *cis*-elements present within the pre-mRNA (Millevoi and Vagner 2010; Colgan and Manley 1997; Zhao *et al.* 1999; Davila Lopez and Samuelsson 2008; Tian *et al.* 2005; Barabino and Keller 1999). The polyadenylation machinery comprises four multi-protein complexes: CPSF (cleavage and polyadenylation specificity factor), CstF (cleavage stimulation factor), CFI, and CFII (cleavage factor I and II, respectively).

**Figure 1:**
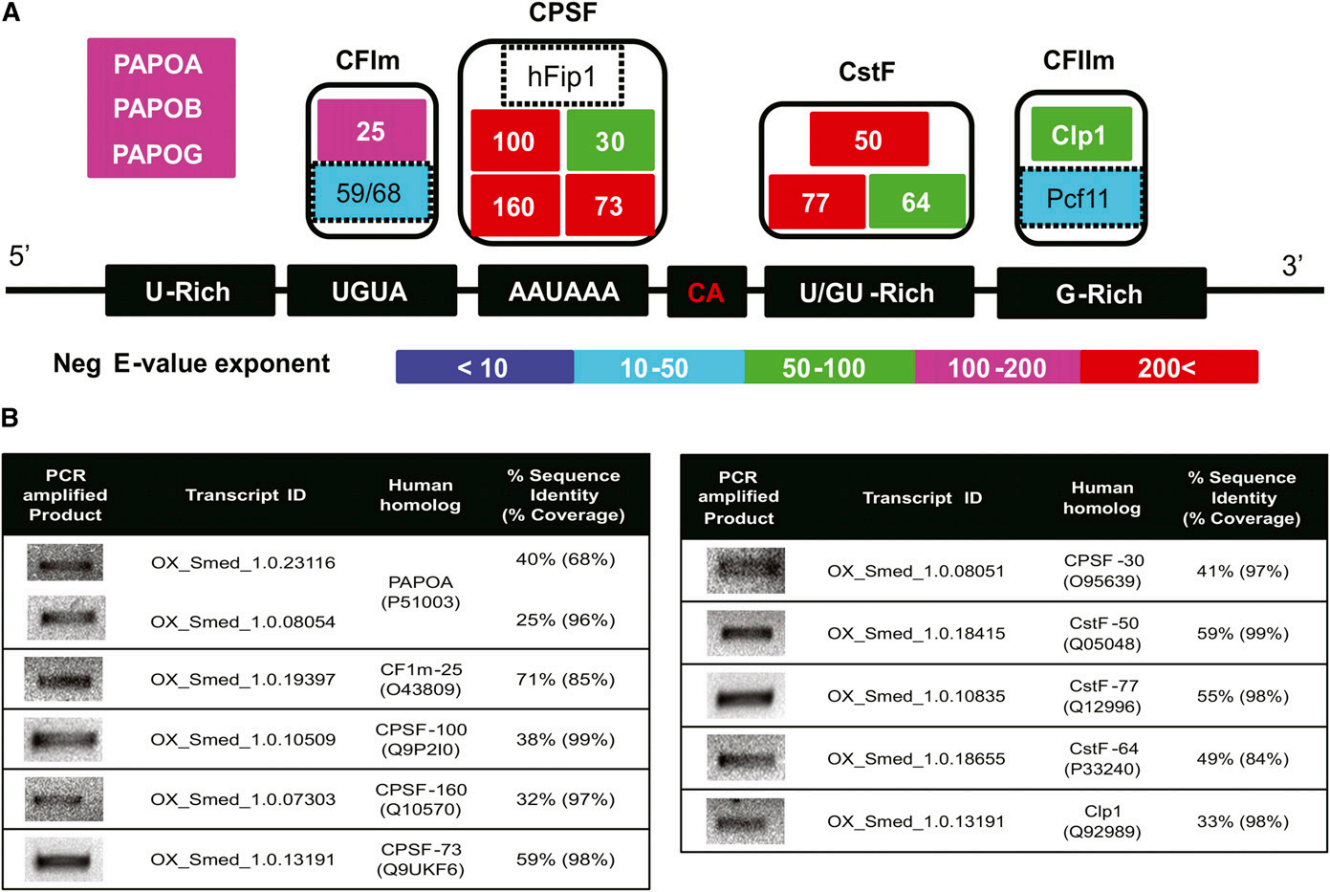
Polyadenylation machinery in planaria. (A) A schematic representing all the components of the eukaryotic polyadenylation machinery in planaria. These specifically include multi-subunit protein complexes such as: Cleavage Factor I (CFIm), Cleavage and Polyadenylation Specificity Factor (CPSF), Cleavage stimulation Factor (CstF), Cleavage factor II (CFIIm), and poly(A) polymerases (PAPOA, PAPOB, or PAPOG). The different protein subunits for each of these complexes are colored based upon the negative E-value exponent returned by BLAST-hit against its human homolog. The components for which the homolog could not be determined confidently in planaria (hFip1, CFIm-59/68, and Pcf11) are marked with dotted lines. The corresponding *cis*-elements recognized by these protein complexes are also shown in the schematic. (B) RT-PCR products showing the expression of the transcripts coding for proteins of the polyadenylation machinery, transcript ID, their corresponding human homolog, percentage sequence identity, and coverage (derived from blast2seq using BLOSUM62 matrix). ID, identity; PCR, polymerase chain reaction.

The components of the polyadenylation machinery in planaria were computationally identified based on their sequence similarity to the known components in the human genome. Of the 16 protein subunits that comprise the polyadenylation machinery, 12 had homologs in planaria (Figure 1A). For the four proteins [hFIP1 (CPSF subunit), CFIm 59/68 (components of CFI), and Pcf11 (component of CFIIm)], confident planarian homologs could not be identified (due to low sequence identity to human homologs; Figure S1, Figure S2, Figure S3, and Figure S4). The presence of transcripts coding for these polyadenylation machinery components were subsequently confirmed through PCR, followed by sequencing (Figure 1B). This suggests that the classical eukaryotic mRNA 3′end-processing machinery is well-conserved in planarians.

### Characterization of polyadenylation sites reveals a conserved mechanism in planarians

In order to determine polyadenylation sites in *S. mediterranea*, poly(A) RNAs were isolated from the whole organism (both sexual and asexual forms separately) and then subjected to 3P-Seq. Sequencing reads thus obtained were mapped to the SxlV3.1 genome using bowtie (Langmead *et al.* 2009). A read was considered as a 3P-tag if the alignment to the genome resulted in a mismatch of “A” at the 3′ end, which would be an untemplated adenosine from the poly(A) tail (Figure S5). The alignment thus obtained was used to derive 3P-peaks, defined as discrete regions on the genome covered by the 3P-tags. Each of these peaks could represent an individual polyadenylation event. In total, 31,377 polyadenylated sites were identified from this analysis (Table S1).

**Figure 2:**
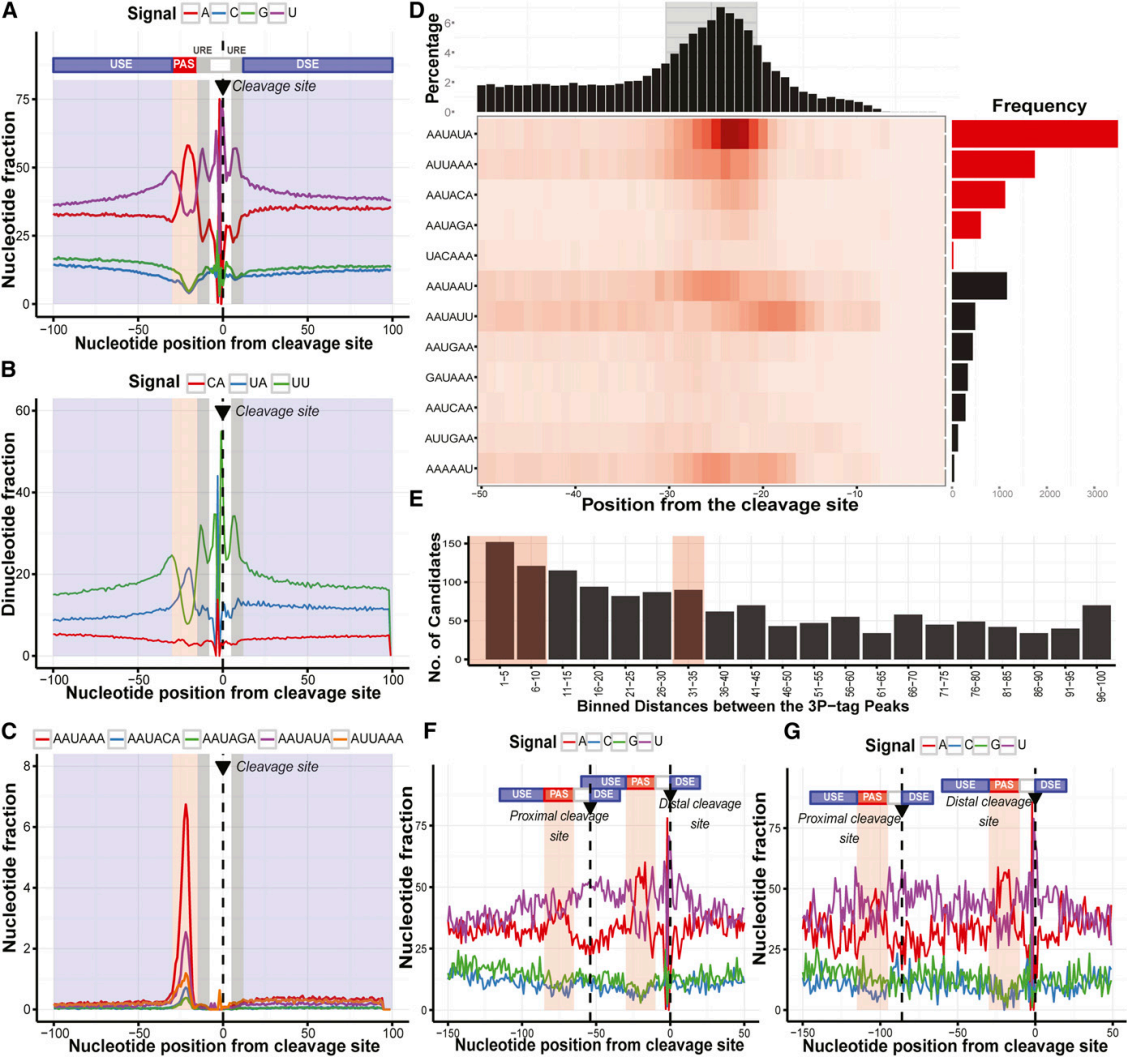
Conservation of polyadenylation signals in planaria. A line-plot showing the (A) mononucleotide profile and (B) the dinucleotide profile (CA, UU, and UA) of all the 31,377 3P-peaks derived from 3P-Seq (poly(A)-position profiling by sequencing). The 0-position on the x-axis denotes the cleavage site (annotated with dotted line and arrow). The upstream elements (USE) and the downstream elements (DSE), along with the polyadenylation signal (PAS) have been separately highlighted in blue and red, respectively. The U-rich element (URE) flanking the cleavage site has been highlighted in gray. (C) A line plot showing the distribution of different hexameric polyadenylation signals on the 3P-peaks, which are specifically recognized by the CPSF (Cleavage and Polyadenylation Specificity Factor subunit). (D) The matrix plot highlighting the distribution of secondary hexameric signals (other than canonical “AAUAAA”) across the 3P-peaks. The histogram in the top panel represents the positional distribution of the hexameric PAS across the 3P-peaks. The panel on the right is the bar plot reporting the frequency of the corresponding hexameric sequences across polyadenylation sites. The hexameric signals that passed the Fisher’s test of independence are highlighted in red. (E) The histogram showing the number of 3P-peaks that were binned based on the genomic distances. The mononucleotide scan was performed for 3P-peaks by fixing the distal cleavage sites that were separated by (F) 10 and (G) 30–35 nucleotides.

Next, we looked for *cis*-regulatory elements associated with the 3P-peaks identified in our study. We found an enrichment of “U” around the cleavage site, a property which is conserved across eukaryotes (Figure 2A). These U-rich regions are specifically recognized by the CFI and CFII complexes. The A-rich region, corresponding to PAS recognized by CPSF, was also observed 10–30 nt upstream of the cleavage site. The CPSF complex cleaves adjacent to a “CA” dinucleotide, which is conserved in eukaryotes (Mandel *et al.* 2006). In planarians, in addition to “CA,” we also observed an enrichment of “UA” and “UU” near the cleavage site (Figure 2B). The CFIm-59/68 (CFI subunit), which recognizes the “UGUA” site, could not be detected in planarians using a sequence homolog search. This is also further supported by the lack of a “UGUA” motif upstream of the PAS in planaria.

In higher eukaryotes, the canonical hexameric sequence of “AAUAAA” is located 10–30 nt upstream of the cleavage site (Figure 2C) (Colgan and Manley 1997). This motif is specifically recognized by the CPSF160 subunit (Schonemann *et al.* 2014). The machinery also tolerates a single/double nucleotide variation within this canonical hexamer. In planaria, we found “AAUAAA” and its four single-nucleotide variants (AAUAUA, AUUAAA, AAUACA, and AAUAGA) enriched 10–30 nt upstream of the cleavage site (Figure 2D). These four hexameric variants have also been reported to act as PAS in other metazoans (You *et al.* 2015) (Figure S6). This is consistent with the results of a motif search using the MEME software (Figure S6). The PAS hexameric signal, including the single and double-nucleotide variants, were found in 98.8% of 3P-peaks. Around 41% of these 3P-peaks contained the canonical “AAUAAA” signal; another 40.4% contained a single-nucleotide variant of the canonical signal; and the remaining 16.3% peaks had two nucleotide variations (Figure S6 and Table S1).

We also determined the resolution of the polyadenylation sites by measuring the genomic distances between adjacent 3P-peaks. The distance is defined by the number of nucleotides between the end of one 3P-peak and the beginning of the neighboring peak. We observed 1390 peaks that were separated by a distance of only 100 nt. The gap between a majority of these 3P-peak pairs was between 10–35 nt (Figure 2E and Figure S8). To rule out the possibility that these short distances are not merely single unresolved polyadenylation sites, we tested for the presence of polyadenylation signals within them. For this analysis, 150 bp spanning both upstream and downstream of the distal cleavage site was used. The enrichment of the U-rich signal (Figure 2, F and G) along with the PAS (determined by fixing the proximal cleavage site, Figure S8) confirmed these to be genuine 3P-peaks.

### Annotation of 3′UTRs in planaria

**Figure 3:**
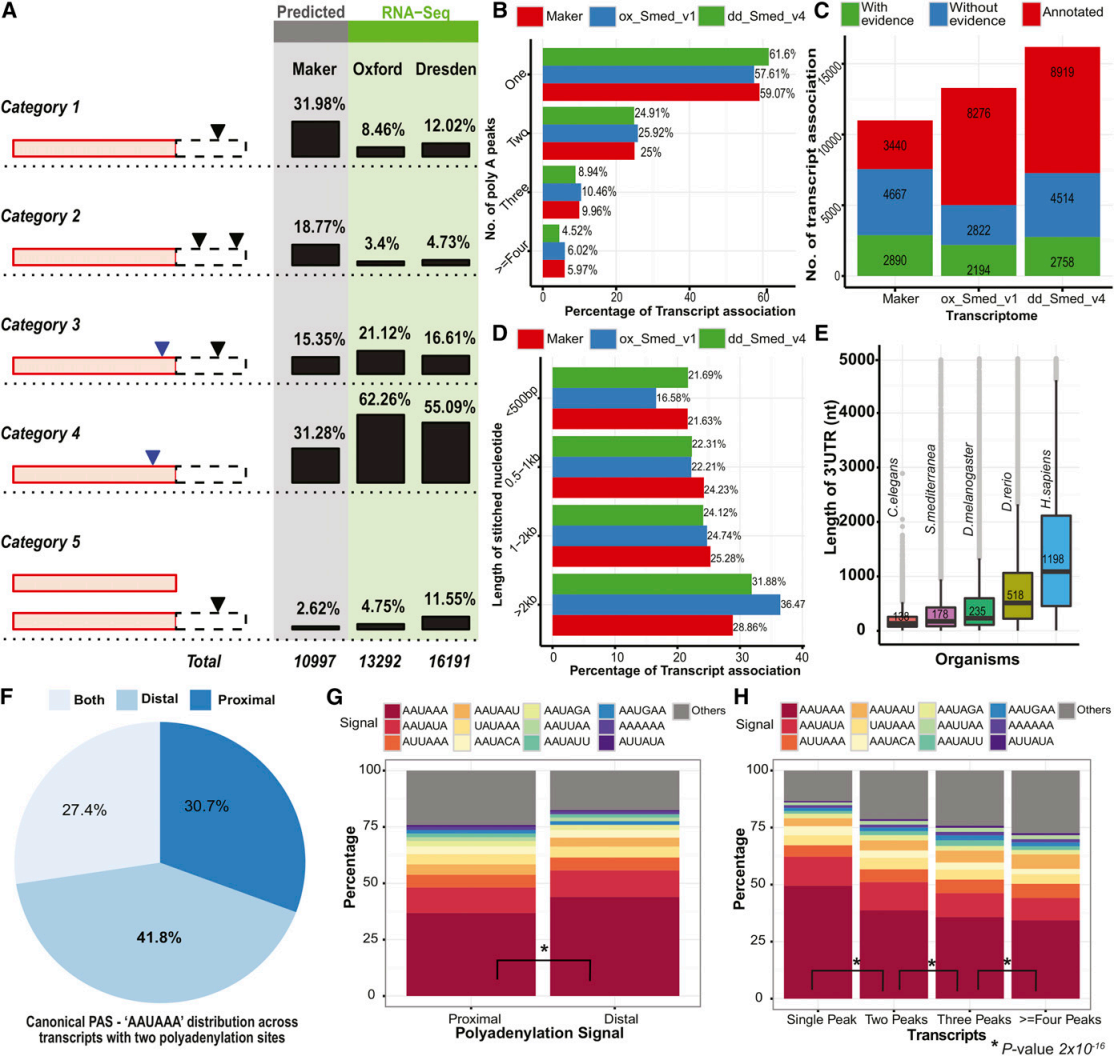
Annotation of 3′UTR sequences in planaria. (A) The association of each 3P-peak to the corresponding transcript was categorized based on the relative positioning of the 3P-peak on the existing transcript model. The red bar represents the annotated part of the transcript and the dotted line represents the unannotated region, downstream of the transcript. The arrows represent the cleavage site that was derived from the 3P-Seq analysis in these unannotated (black) and annotated (blue) regions of the transcript. The association was carried out using three different transcriptomes (Makers, Ox_Smed_v1, and dd_smed_v4). The percentage association of each category across different transcriptomes are also depicted. (B) A bar plot showing the number of 3P-peaks [poly(A) peaks] associated with each of the transcripts across different transcriptomes. (C) Stacked bar plot representing the number of transcripts that could be extended further based upon the 3P-peaks associated with them. (D) The bar plot highlights the length of the stitched region for the transcripts across different transcriptomes. The percentage denotes the total number of transcripts that were extended by specific length across the three different transcriptomes. (E) Box plot illustrating the distribution of 3′UTR length across different species: *C. elegans*, *S. mediterranea*, *D. melanogaster*, *Danio rerio*, and *Homo sapiens*. The data for the length of the 3′UTRs were derived from the Ensemble database. (F) Pie chart revealing the presence of the canonical “AAUAAA” signal across transcripts associated with two polyadenylation sites. (G) Stacked bar plot illustrating the occurrences of different hexameric PAS across proximal and distal polyadenylation sites. (H) Stacked bar plot reflecting the abundance of different hexameric PAS across transcripts associated with one and multiple 3P-peaks. 3P-Seq, poly(A)-position profiling by sequencing; PAS, poly(A) signals; UTR, untranslated region.

We associated each transcript to its nearest 3P-peak to determine its polyadenylation site. For this, we used three different transcriptome models of planaria: a) Maker, a computational pipeline that performs abinitio gene prediction and annotation of the genome (Cantarel *et al.* 2008); b) Ox_Smed_v1 (Blythe *et al.* 2010); and c) dd_Smed_v4 (Liu *et al.* 2013), both of which are derived from *de novo* transcriptome assemblies of RNA-Seq data. The polyadenylation sites obtained could be categorized depending upon its position with respect to the transcript model (Figure 3A, and Table S2). Around 92% of these polyadenylation sites were present on the terminal exon (Category 4, Figure S9). We also observed polyadenylation sites (43.51%) outside the transcript models (Category 1 in Figure 3A), emphasizing that these transcripts could have longer 3′UTRs than previously annotated. We found that ∼40% of transcripts were associated with more than one polyadenylation site (Figure 3B), which is close to the estimated ApA in other eukaryotes (Derti *et al.* 2012). For those transcripts with a polyadenylation site outside the annotated transcript, we extended the transcript to include the nearest 3P-peak. Various sources of evidence such as RNA-Seq reads, Genscan (Burge and Karlin 1997), and Genpred were used to confirm the extended transcripts (Figure 3C and Figure S9). Around 70% of the extended transcripts, across the three transcriptomes, had a length < 2 kb (Figure 3D). For 213 transcripts, we were able to extend the protein coding region by more than 15 amino acids (Figure S9). For 21 of these transcripts, we were able to assign pfam domains to the extended region (Figure S9). Around 9972 unique 3P-peaks that were mapped to genomic contigs had no transcript annotations (Category 6, Figure S9). These could be putative long noncoding RNAs (Ulitsky *et al.* 2011), which needs to be further characterized. We also observed cleavage sites that were in the opposite orientation to a neighboring transcript, which suggests the presence of antisense transcripts in these loci (Category 7, Figure S9). To remove the redundancy arising from the pooling of transcripts from three different datasets, we clustered all protein sequences (coded by longest ORFs) based on sequence similarity using Cd-hit (Li and Godzik 2006). A high sequence identity cut-off of 90% was used to retain the paralogs. This resulted in 31,990 gene clusters (Figure S10 and Table S3). The longest protein sequence from each cluster was chosen as representative of that cluster to identify the length of 3′UTRs. The median length of the 3′UTR was found to be around 176 bp, as compared to 138 in *C. elegans*, 235 in *Drosophila*, 518 in zebrafish, and 1198 in humans (Figure 3E).

We found that 3639 transcripts contained two polyadenylation sites. We analyzed the occurrence of hexameric sequence motifs in these 3P-peaks. The canonical hexameric sequence “AAUAAA” was enriched in the distal compared to the proximal site (Figure 3, F and G; *t*-test unpaired: *P*-value < 2 x $10^{-16}$). The overall abundance of this canonical “AAUAAA” hexameric signal decreases with the increase in the number of polyadenylation sites per transcript (Figure 3H, *t*-test unpaired: *P*-value < 2 x $10^{-16}$). This suggests that most of the transcripts associated with multiple 3P-peaks have a combination of strong (canonical), and a variant of the canonical, signals, which could have evolved to play a regulatory role. Further, we also explored the association of the canonical signal and its variants with the expression levels of the transcripts, but we did not find any correlation between the expression levels of the transcripts and the corresponding hexameric PAS signals associated with them (Figure S11).

### Validation and confirmation of cleavage sites and polyadenylation signals in planarians

**Figure 4:**
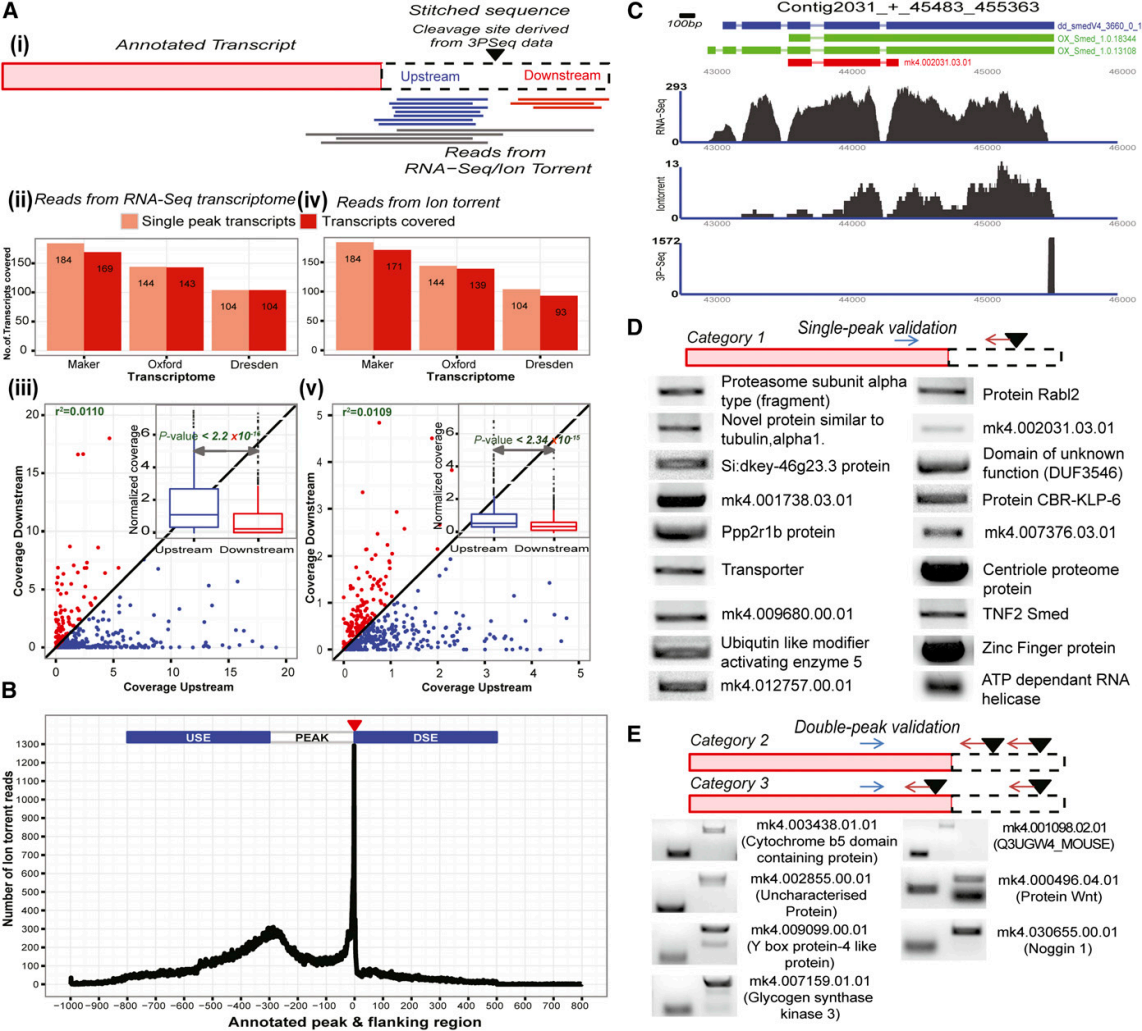
Validation of polyadenylation sites and transcript association. (A) A schematic highlighting the coverage of ion-torrent reads (black) and RNA-seq reads in the regions upstream (red) and downstream (blue) of polyadenylation sites determined from 3P-Seq (i). The bar plot shows the comparison between the number of transcripts associated with one 3P-peak and those transcripts that were also mapped by the RNA-seq reads (ii). The scatter plot depicts the transcripts with the coverage of RNA-seq reads in the inner (blue) and outer (red) region of the polyadenylation site (iii). The bar plot depicts comparison of the number of transcripts associated with one 3P-peak and those transcripts that were mapped by ion-torrent reads across different transcriptomes (iv). The scatter plot depicts the transcripts with the coverage derived from the ion-torrent reads aligned to the upstream and downstream regions of the cleavage site (v). (B) The coverage of ion-torrent reads for the region spanning the 3P-peaks. (C) A coverage plot showing the coverage of RNA-seq reads and ion-torrent reads across different transcript models for ARS2 domain-containing protein. The corresponding 3P-peak derived for it supports the ox_Smed_V1 and dd_Smed_V4 models. (D) Single peak candidates that were experimentally validated through PCR amplification. A schematic highlighting the strategy for designing forward (blue) and reverse primers (red). (E) The transcripts with two 3P-peaks were experimentally validated using PCR. The upper band represents the amplification of longer transcript isoform and the lower band represents the shorter form of the transcript. 3P-Seq, poly(A)-position profiling by sequencing; PCR, polymerase chain reaction; RNA-Seq, RNA sequencing.

The association of transcripts with polyadenylated sites derived from 3P-Seq were validated using various approaches. Transcripts with a single 3P-peak were specifically selected and their 3′ UTR expression probed using publicly available RNA-Seq data (Resch *et al.* 2012). We found that the region upstream of the polyadenylation site has more RNA-Seq coverage in comparison to its downstream region (Figure 4A and Figure S12; Wilcox test, paired: *P*–value < 2.2 × $10^{-16}$). This difference in RNA-Seq coverage increases with the strength (number of 3P-tags) of the 3P-peak (Figure S12). Although we do see a few outliers with more reads in the downstream, most of these outliers may code for putative transcripts (as determined by Genscan, Genpred, and EST databases) (Figure S12). The other possible explanation for these outliers could be the presence of a downstream polyadenylation signal that could not be captured by the current depth of 3P-Seq.

The 3′UTRs derived from 3P-Seq were further confirmed through ion-torrent sequencing (Rothberg *et al.* 2011) of polyadenylated mRNAs. This technology adopts a different chemistry optimized to derive reads of longer length (100–300 bp, Figure S12). The reads derived from this ion-torrent sequencing were trimmed to remove the polyadenylated tail and subsequently mapped to the genome. The alignment covered around 42% of the 3P-peaks derived from 3P-Seq, and the majority of these reads mapped upstream of the cleavage site, thus validating the 3P-seq data (Figure 4B). Thus, RNA-Seq reads and ion-torrent reads coupled to 3P-Seq data from this study can help to obtain complete gene models in planaria (Figure 4C).

Some of the transcripts associated with polyadenylation sites were also experimentally validated using RT-PCR and Sanger sequencing. The validation (Figure 4D) clearly suggests that existing transcript models coding for TNF2, Rabl2, and Ppp2r1b can be extended to capture the complete 3′UTR. Some transcripts with two polyadenylation sites were also experimentally validated through RT-PCR. We found that genes involved in the regulation of cell fate and patterning like *smed-wnt*, *smed-noggin*, and *smed-ybox-4* undergo ApA.

### Biological functions mediated by alternate polyadenylation

Around 40% of the transcripts in planaria were associated with more than one polyadenylation site, which emphasizes the need to explore the biological significance of ApA. A Gene Ontology (GO) enrichment analysis was carried out for these transcripts. The following biological processes were significantly enriched: hemophilic cell adhesion via plasma membrane (GO:0007156), transmembrane transport (GO:0055085), the G-protein coupled receptor-signaling pathway (GO:0007186), and ion transport (GO:0006811). We also observed the enrichment of genes involved in cell cycle arrest (GO:0007050) and the regulation of sequence-specific DNA-binding transcription factors (Table S4). The molecular functions that were enriched included: zinc-ion binding (GO:0008270), DNA/nucleic acid binding (GO:0003677, GO:0003676), protein serine/threonine kinase activity (GO:0004674), protein dimerization activity (GO:0046983). and ribonuclease activity (GO:0004540) (Figure S13 and Table S4).

Candidate-based approaches have previously reported that ApA plays a role in regulating biological processes such as the production of membrane-bound and secreted immunoglobins (Alt *et al.* 1980; Rogers *et al.* 1980; Early *et al.* 1980), T-cell activation (Sandberg *et al.* 2008), hippocampal neuron stimulation (Flavell and Greenberg 2008), embryonic development (Ji *et al.* 2009; Mangone *et al.* 2010; Hilgers *et al.* 2011), lymphomas (Singh *et al.* 2009), and breast cancer (Fu *et al.* 2011). We performed a literature survey to assemble a list of genes known to be regulated by ApA (Mayr and Bartel 2009; Smibert *et al.* 2012; Sun *et al.* 2012). Around 70 genes were identified by this survey (Table S5) and homologs for 22 of them could be found in planaria. Of these, 10 were associated with more than one polyadenylation signal.

### Functional consequences of alternate polyadenylation (ApA)

**Figure 5:**
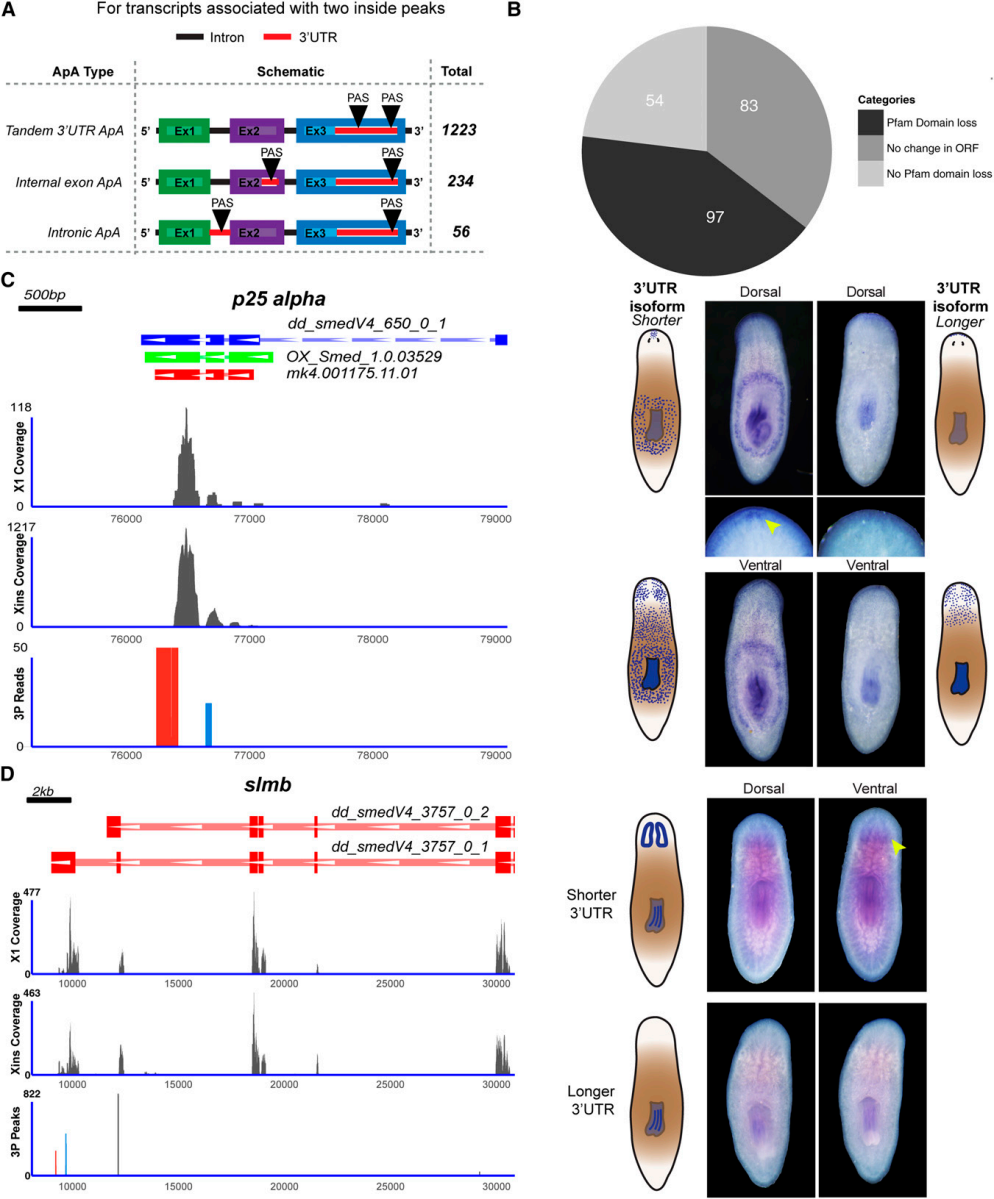
Functional consequences of alternate polyadenylation. (A) Schematic highlighting the classification of the alternate polyadenylation events based upon the existing transcriptome model. (B) Pie chart showing the polyadenylation events in the internal exons that could potentially lead to either loss of domain or no change in ORF. (C) The RNA-Seq coverage plot for OX_Smed.1.0.03529 (p25α) from X1, Xins population along with 3P-peaks is shown on the left panel. The corresponding whole mount *in situ* hybridization (WISH) patterns for the p25α is shown on the right. (D) Similar to (C), RNA-seq coverage and WISH patterns for the dd_smed_V4_3757_0_1 (*slmb*) are shown. ORF, open reading frame; RNA-Seq, RNA sequencing; UTR, untranslated region.

**Figure 6:**
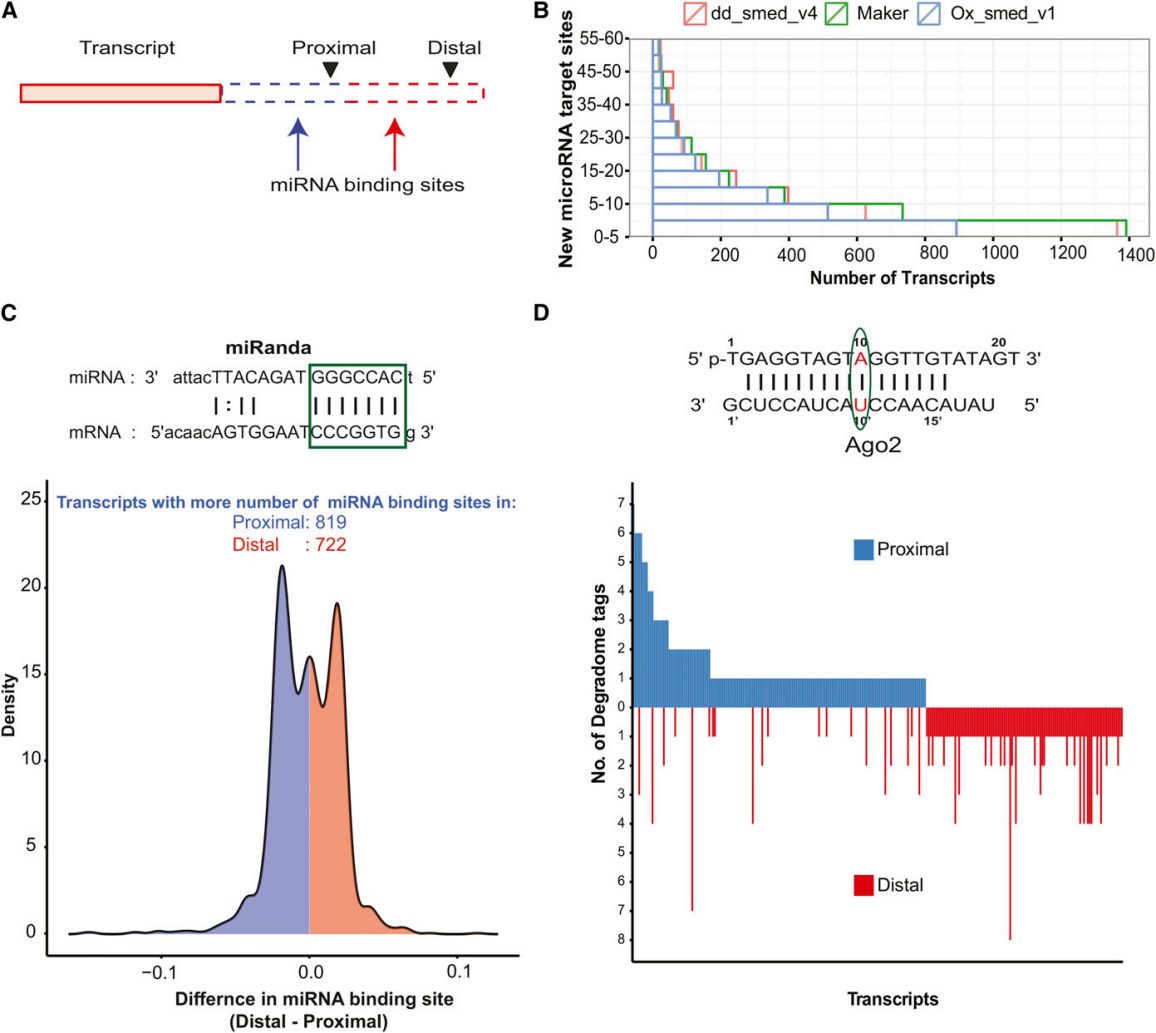
Effect of altered 3′UTR on miRNA-mediated gene regulation. (A) Schematic describing the location of miRNA binding sites with respect to the proximal and distal polyadenylation events. (B) The miRNA binding sites that were predicted to be associated with stitched transcripts across different transcriptomes. (C) The distribution of miRNA binding sites on the transcripts at the regions spanning the proximal and distal polyadenylation sites. (D) The bar plot shows the number of the degradome tags mapped to the transcripts on either the proximal (positive y-axis) or distal (negative y-axis) polyadenylation site. miRNA, microRNA; UTR, untranslated region.

For transcripts containing two 3P-peaks, we investigated their location relative to the known transcript model. These peaks could either be located on the 3′UTR, giving rise to tandem 3′UTR ApA, or on the coding regions resulting in coding region polyadenylation (CR-ApA) (Elkon *et al.* 2013). For a majority of transcripts (∼80.1%, 1213 transcripts), the multiple ApA sites were all located on the last exon resulting in tandem 3′ UTR ApA (Figure 5A). This tandem 3′UTR ApA results in mRNA with an altered 3′UTR sequence and thus, could regulate posttranscriptional gene expression. The altered 3′UTR produced by ApA can result in a loss or gain of miRNA binding sites (Figure 6A). Hence, a systematic search was performed to predict miRNA binding sites within the 3′UTRs of the transcripts using miRanda (John *et al.* 2004). This is the first comprehensive prediction of miRNA binding sites in the planarian genome (Figure 6B). We defined the proximal 3′UTR to include the region from the end of the last exon to the proximal cleavage site. The distal 3′UTR involved the region between the proximal and the distal cleavage site. Thus, we ensured that both these regions had no overlap for miRNA binding site prediction. For transcripts with two polyadenylated sites, we observed that miRNA binding sites are preferentially located either at the proximal or the distal 3′UTR. This is reflected by the bimodal distribution of the occurrence of these sites along the transcript. This trend was rarely observed in a randomized control dataset (Figure 6C and Figure S14). Around 14 out of 100 randomized datasets displayed a bimodal distribution (as reported by Hartigan’s dip test with *P*-value < 0.05), but seven of these bimodal distributions were qualitatively different from the actual profile (Figure S14).

We further performed degradome sequencing to experimentally identify the miRNA targets in the planarian transcriptome. This method has been previously used to identify miRNA targets in other metazoans (Cass *et al.* 2016; Karginov *et al.* 2010). The reads that map to the miRNA targets are those that are cleaved by the AGO machinery because of the complementarity at the ninth, 10th, and 11th nucleotides. The targets that were translationally repressed will not be captured by this method. The mapped reads that represent the miRNA binding sites on the transcript were annotated as degradome tags (Table S6). We identified around 219 tags that mapped to 259 transcripts having two polyadenylation sites associated with them. These tags mapped exclusively either to the proximal (135 transcripts) or the distal (104 transcripts) 3′UTRs of the transcripts. Very few transcripts (20 transcripts) had an equal number of degradome tags on both the proximal and the distal 3′UTR (Figure 6D).

Later, we also investigated ApA events that alter the protein coding sequence and subsequently its biological function. Around 19% of the transcripts had polyadenylation sites located within introns/exons; these are called coding region alternate polyadenylation (CR-ApA) sites. We found ApA sites on more than one exon for around 270 transcripts (Figure 5B and Table S7). Of these, 97 could potentially result in truncated proteins (Table S7). We found that truncation could result in the loss of certain domains from these proteins. These domains include an EF-hand domain pair, a WD domain, tetracopeptide repeats, dynein heavy chain region D6 of dynein motor, a myosin tail, Ras family, and a methyltransferase domain.

We performed whole mount *in situ* hybridization (WISH) to study the expression pattern of alternatively polyadenylated transcripts dd_smed_V4_650_0_1 (p25α) and dd_smed_V4_3757_0_1 (*slmb*). The *in situ* probe designed from the region upstream of the proximal PAS signal will recognize both the isoforms, whereas the probe designed from the region immediately upstream of the distal PAS signal will specifically hybridize to the longer isoform of the transcript. The whole mount *in situ* hybridization using the probe, which recognizes both the isoforms of the transcript p25α showed expression in the anterior-most region of the head, with characteristic expression at the dorsal tip and around the pharynx. The longer isoform of the transcript was expressed in the anterior-most region of the head but its expression was not seen at the dorsal tip. Weak expression of the longer isoform was seen around the pharyngeal region. The other transcript, dd_smed_V4_3757_0_1 (*slmb*), has also been reported to be alternatively polyadenylated in *Drosophila* (Smibert *et al.* 2012). The *in situ* hybridization for dd_smed_V4_3757_0_1 using the probe designed to recognize both the isoforms showed its expression in the pharynx, photoreceptor, and cephalic ganglion, whereas the probe designed to recognize the longer isoform revealed its expression only within the pharyngeal region (Figure 5D). These results demonstrate the tissue-specific expression of ApA isoforms, which implies the role of ApA in tissue organization and function in planaria.

## Discussion

This study is the first to report genome-scale polyadenylation events in *S. mediterranea* using poly(A) position profiling (3P-Seq). An in-house computational pipeline was developed to reliably identify the cleavage site and to determine the PAS associated with it. Nearly 14,000 transcripts could be annotated with their 3′UTRs. The analysis presented here was done using the older version of the genome. Recently, a newer version of the Smed genome (SxlV4 and AsxlVs1) was released that contains 15,334 scaffolds in comparison to the 43,294 contigs in the previous SxlV3.1 genome (Robb *et al.* 2015). We repeated the analysis using the new genome to determine the extent of overlap of 3P-peaks between the new and old genome. Around 83% of the 3P-peaks determined using the new genome overlapped (≥ 50% overlap) with the previously determined polyadenylation sites using the SxlV3.1 genome (Figure S15 and Table S8), which lends support to our approach of annotating the 3′ UTR from the 3P-seq data.

Our study also identified most of the proteins involved in the cleavage/polyadenylation process with high confidence, except for FIP-1, Pcf11, CFI, and CFII. The best hits obtained for these four genes, along with 12 other genes of the polyadenylation machinery, showed the evidence for their expression in the transcriptome databases (Onal *et al.* 2012; Labbé *et al.* 2012; Resch *et al.* 2012). This suggests that they are not merely hypothetical proteins and are actively expressed within the planarian system. Interestingly, the cell type-specific expression data from these transcriptomes revealed the enrichment (> twofold) of almost all the transcripts of the polyadenylation machinery excluding PAPγ (no change), in the proliferating neoblast (X1) when compared to differentiated cells (Xins). Further experiments would be required to decipher the biological significance of the enrichment. In other metazoans, FIP-1 typically binds to U-rich elements (URE) spanning the cleavage/polyadenylated site (Kaufmann *et al.* 2004). Though we identified URE in the 3′ UTR regions of the planarians, the inability to identify the FIP-1 homolog could be due to the high sequence divergence, which cannot be detected using standard homology based searches. The proteins CFIm and Pcf11 (Noble *et al.* 2007), subunits of the CFII complex, recognize a “UGUA” signal in other eukaryotes. In planarians, lack of a “UGUA” signal at the cleavage site in 3′ UTR regions could potentially explain the absence/divergence of CFIm and Pcf11 protein subunits.

In planarians, the canonical hexameric signal “AAUAAA” is the most prevalent polyadenylation signal. We identified several variants of this canonical signal, which were also reported in other metazoans. They function as secondary signals, which were less preferred for polyadenylation compared to the canonical signals (You *et al.* 2015). In planarians, for those transcripts with more than one 3P-peak, the distal 3P-peaks were mostly associated with a canonical hexameric signal, whereas the proximal 3P-peaks were associated with the secondary signal variants. This trend was also observed in other metazoans, which supports the hypothesis proposed by Shi (2012) that distal PAS may be recognized as default polyadenylation site by the machinery as it is highly conserved, whereas the proximal PAS could mostly play a regulatory role and is less conserved.

Here, we also showed that > 40% of the planarian transcripts were alternately polyadenylated. We identified transcripts that contained the polyadenylation signal in the internal exon that could lead to altered protein products. One such candidate, p25α, was experimentally validated using *in situ* hybridization. This protein is reported to stimulate the process of tubulin polymerization and also play a key role in α-synuclein aggregation. However, most of the transcripts that undergo ApA have alternate PAS in the 3′ UTR regions, which will result in mRNAs with distinct 3′ UTRs. We validated the presence of altered 3′UTR for *slmb* (supernumerary limb gene) through *in situ* hybridization (ISH). The *slmb* gene is a member of the F-box/WD40 protein family of the ubiquitin ligase SCF complex that targets phosphorylated proteins for degradation. In addition to these transcripts that undergo tissue-specific alternate polyadenylation, we also identified 1606 ApA transcripts that were enriched by at least twofold in the neoblast population by analyzing the previously published RNA-Seq data (Onal *et al.* 2012) (Table S10). However, the ApA status of these candidates in the neoblast population needs to be validated. This observation suggests that ApA could potentially play an important role in the regulation of gene expression in the neoblast population. The 3′UTR is one of the key regulatory elements that determine the fate of the mRNA within the cell. By altering the length of the 3′ UTR in a given cell type, the transcript may either get destabilized leading to translational repression or undergo active translation, mediated by RNA-binding proteins that bind to the 3′ UTR. The microRNAs, which are translational repressors, have their binding sites on the 3′ UTRs. microRNA binding could be altered by changing the length of the 3′ UTR. In planarians, we observed that miRNA binding sites on the transcripts with two 3P-peaks were either present upstream of the proximal 3P-peak or the distal 3P-peak in a mutually exclusive manner. Together, these results support the observation that miRNA-mediated regulation of gene expression could be altered by ApA events.

In summary, our study demonstrates 3P-Seq as a powerful tool to identify alternate polyadenylation events on a genome-wide scale. Our analysis is one of the first to provide a detailed account of the 3′UTR landscape in planaria. This study will facilitate the exploration of various posttranscriptional regulatory events mediated by the 3′UTRs. More detailed study of polyadenylation events across tissue types and regenerating tissues will provide better insights into the role of ApA in the regulation of tissue homeostasis, stem cell function, and regeneration in planarians. This study will also provide a platform to explore the posttranscriptional regulatory mechanisms mediated through 3′UTRs in planaria.

## 

## Supplementary Material

Supplemental Material

## References

[bib1] Addo-Quaye C. Miller W. Axtell M. J., 2009 Cleaveland: a pipeline for using degradome data to find cleaved small RNA targets. Bioinformatics 25: 130–131. 19017659 10.1093/bioinformatics/btn604 PMC3202307

[bib2] Alt F. W. Bothwell A. L. Knapp M. Siden E. Mather E., 1980 Synthesis of secreted and membrane-bound immunoglobulin mu heavy chains is directed by mRNAs that differ at their 3′ ends. Cell 20: 293–301. 6771018 10.1016/0092-8674(80)90615-7

[bib3] Altschul S. F. Gish W. Miller W. Myers E. W. Lipman D. J., 1990 Basic local alignment search tool. J. Mol. Biol. 215: 403–410. 2231712 10.1016/S0022-2836(05)80360-2

[bib4] Bailey T. L. Williams N. Misleh C. Li W. W., 2006 MEME: discovering and analyzing DNA and protein sequence motifs. Nucleic Acids Res. 34: W369–373. 16845028 10.1093/nar/gkl198 PMC1538909

[bib5] Barabino S. M. Keller W., 1999 Last but not least: regulated poly(A) tail formation. Cell 99: 9–11. 10520989 10.1016/s0092-8674(00)80057-4

[bib6] Barrett L. W. Fletcher S. Wilton S. D., 2012 Regulation of eukaryotic gene expression by the untranslated gene regions and other non-coding elements. Cell. Mol. Life Sci. 69: 3613–3634. 22538991 10.1007/s00018-012-0990-9 PMC3474909

[bib7] Berkovits B. D. Mayr C., 2015 Alternative 3′ UTRs act as scaffolds to regulate membrane protein localization. Nature 522: 363–367. 25896326 10.1038/nature14321 PMC4697748

[bib8] Blythe M. J. Kao D. Malla S. Rowsell J. Wilson R., 2010 A dual platform approach to transcript discovery for the planarian Schmidtea mediterranea to establish RNAseq for stem cell and regeneration biology. PLoS One 5: e15617. 21179477 10.1371/journal.pone.0015617 PMC3001875

[bib9] Brandl H. Moon H. Vila-Farre M. Liu S. Y. Henry I., 2015 PlanMine - a mineable resource of planarian biology and biodiversity. Nucleic Acids Res. 44: D764–D773. 10.1093/nar/gkv1148 PMC4702831 26578570

[bib10] Burge C. Karlin S., 1997 Prediction of complete gene structures in human genomic DNA. J. Mol. Biol. 268: 78–94. 9149143 10.1006/jmbi.1997.0951

[bib11] Cantarel B. L. Korf I. Robb S. M. Parra G. Ross E., 2008 MAKER: an easy-to-use annotation pipeline designed for emerging model organism genomes. Genome Res. 18: 188–196. 18025269 10.1101/gr.6743907 PMC2134774

[bib12] Cass A. A. Bahn J. H. Lee J.-H. Greer C. Lin X., 2016 Global analyses of endonucleolytic cleavage in mammals reveal expanded repertoires of cleavage-inducing small rnas and their targets. Nucleic Acids Res. 44: 3253–3263. 26975654 10.1093/nar/gkw164 PMC4838385

[bib13] Colgan D. F. Manley J. L., 1997 Mechanism and regulation of mRNA polyadenylation. Genes Dev. 11: 2755–2766. 9353246 10.1101/gad.11.21.2755

[bib14] Davila Lopez M. Samuelsson T., 2008 Early evolution of histone mRNA 3′ end processing. RNA 14: 1–10. 17998288 10.1261/rna.782308 PMC2151031

[bib15] Derti A. Garrett-Engele P. Macisaac K. D. Stevens R. C. Sriram S., 2012 A quantitative atlas of polyadenylation in five mammals. Genome Res. 22: 1173–1183. 22454233 10.1101/gr.132563.111 PMC3371698

[bib16] Early P. Rogers J. Davis M. Calame K. Bond M., 1980 Two mRNAs can be produced from a single immunoglobulin mu gene by alternative RNA processing pathways. Cell 20: 313–319. 6771020 10.1016/0092-8674(80)90617-0

[bib17] Elkayam E. Kuhn C.-D. Tocilj A. Haase A. D. Greene E. M., 2012 The structure of human argonaute-2 in complex with mir-20a. Cell 150: 100–110. 22682761 10.1016/j.cell.2012.05.017 PMC3464090

[bib18] Elkon R. Ugalde A. P. Agami R., 2013 Alternative cleavage and polyadenylation: extent, regulation and function. Nat. Rev. Genet. 14: 496–506. 23774734 10.1038/nrg3482

[bib19] Flavell S. W. Greenberg M. E., 2008 Signaling mechanisms linking neuronal activity to gene expression and plasticity of the nervous system. Annu. Rev. Neurosci. 31: 563–590. 18558867 10.1146/annurev.neuro.31.060407.125631 PMC2728073

[bib20] Fu Y. Sun Y. Li Y. Li J. Rao X., 2011 Differential genome-wide profiling of tandem 3′ UTRs among human breast cancer and normal cells by high-throughput sequencing. Genome Res. 21: 741–747. 21474764 10.1101/gr.115295.110 PMC3083091

[bib21] German M. A. Pillay M. Jeong D. H. Hetawal A. Luo S., 2008 Global identification of microRNA-target RNA pairs by parallel analysis of RNA ends. Nat. Biotechnol. 26: 941–946. 18542052 10.1038/nbt1417

[bib22] Griffiths-Jones S. Saini H. K. van Dongen S. Enright A. J., 2008 miRBase: tools for microRNA genomics. Nucleic Acids Res. 36: D154–D158. 17991681 10.1093/nar/gkm952 PMC2238936

[bib23] Hilgers V. Perry M. W. Hendrix D. Stark A. Levine M., 2011 Neural-specific elongation of 3′ UTRs during Drosophila development. Proc. Natl. Acad. Sci. USA 108: 15864–15869. 21896737 10.1073/pnas.1112672108 PMC3179109

[bib24] Hubert A. Henderson J. M. Ross K. G. Cowles M. W. Torres J., 2013 Epigenetic regulation of planarian stem cells by the SET1/MLL family of histone methyltransferases. Epigenetics 8: 79–91. 23235145 10.4161/epi.23211 PMC3549883

[bib25] Huerta-Cepas J. Dopazo J. Gabaldon T., 2010 ETE: a python Environment for Tree Exploration. BMC Bioinformatics 11: 24. 20070885 10.1186/1471-2105-11-24 PMC2820433

[bib26] Jan C. H. Friedman R. C. Ruby J. G. Bartel D. P., 2011 Formation, regulation and evolution of Caenorhabditis elegans 3′UTRs. Nature 469: 97–101. 21085120 10.1038/nature09616 PMC3057491

[bib27] Ji Z. Tian B., 2009 Reprogramming of 3′ untranslated regions of mRNAs by alternative polyadenylation in generation of pluripotent stem cells from different cell types. PLoS One 4: e8419. 20037631 10.1371/journal.pone.0008419 PMC2791866

[bib28] Ji Z. Lee J. Y. Pan Z. Jiang B. Tian B., 2009 Progressive lengthening of 3′ untranslated regions of mRNAs by alternative polyadenylation during mouse embryonic development. Proc. Natl. Acad. Sci. USA 106: 7028–7033. 19372383 10.1073/pnas.0900028106 PMC2669788

[bib29] Jia J. Yao P. Arif A. Fox P. L., 2013 Regulation and dysregulation of 3′UTR-mediated translational control. Curr. Opin. Genet. Dev. 23: 29–34. 23312843 10.1016/j.gde.2012.12.004 PMC3644486

[bib30] John B. Enright A. J. Aravin A. Tuschl T. Sander C., 2004 Human MicroRNA targets. PLoS Biol. 2: e363. 15502875 10.1371/journal.pbio.0020363 PMC521178

[bib31] Karginov F. V. Cheloufi S. Chong M. M. W. Stark A. Smith A. D., 2010 Diverse endonucleolytic cleavage sites in the mammalian transcriptome depend upon microRNAs, Drosha, and additional nucleases. Mol. Cell 38: 781–788. 20620951 10.1016/j.molcel.2010.06.001 PMC2914474

[bib32] Kaufmann I. Martin G. Friedlein A. Langen H. Keller W., 2004 Human Fip1 is a subunit of CPSF that binds to U-rich RNA elements and stimulates poly(A) polymerase. EMBO J. 23: 616–626. 14749727 10.1038/sj.emboj.7600070 PMC1271804

[bib33] Labbé R. M. Irimia M. Currie K. W. Lin A. Zhu S. J., 2012 A comparative transcriptomic analysis reveals conserved features of stem cell pluripotency in planarians and mammals. Stem Cells 30: 1734–1745. 22696458 10.1002/stem.1144 PMC4161212

[bib34] Langmead B. Trapnell C. Pop M. Salzberg S. L., 2009 Ultrafast and memory-efficient alignment of short DNA sequences to the human genome. Genome Biol. 10: R25. 19261174 10.1186/gb-2009-10-3-r25 PMC2690996

[bib35] Li W. Godzik A., 2006 Cd-hit: a fast program for clustering and comparing large sets of protein or nucleotide sequences. Bioinformatics 22: 1658–1659. 16731699 10.1093/bioinformatics/btl158

[bib36] Liu S. Y. Selck C. Friedrich B. Lutz R. Vila-Farre M., 2013 Reactivating head regrowth in a regeneration-deficient planarian species. Nature 500: 81–84. 23883932 10.1038/nature12414

[bib37] Mandel C. R. Kaneko S. Zhang H. Gebauer D. Vethantham V., 2006 Polyadenylation factor CPSF-73 is the pre-mRNA 3′-end-processing endonuclease. Nature 444: 953–956. 17128255 10.1038/nature05363 PMC3866582

[bib38] Mangone M. Manoharan A. P. Thierry-Mieg D. Thierry-Mieg J. Han T., 2010 The landscape of C. elegans 3′UTRs. Science 329: 432–435. 20522740 10.1126/science.1191244 PMC3142571

[bib39] Mayr C. Bartel D. P., 2009 Widespread shortening of 3′UTRs by alternative cleavage and polyadenylation activates oncogenes in cancer cells. Cell 138: 673–684. 19703394 10.1016/j.cell.2009.06.016 PMC2819821

[bib40] Millevoi S. Vagner S., 2010 Molecular mechanisms of eukaryotic pre-mRNA 3′ end processing regulation. Nucleic Acids Res. 38: 2757–2774. 20044349 10.1093/nar/gkp1176 PMC2874999

[bib41] Moran Y. Fredman D. Praher D. Li X. Z. Wee L. M., 2014 Cnidarian microRNAs frequently regulate targets by cleavage. Genome Res. 24: 651–663. 24642861 10.1101/gr.162503.113 PMC3975064

[bib42] Noble C. G. Beuth B. Taylor I. A., 2007 Structure of a nucleotide-bound Clp1-Pcf11 polyadenylation factor. Nucleic Acids Res. 35: 87–99. 17151076 10.1093/nar/gkl1010 PMC1761425

[bib43] Onal P. Grun D. Adamidi C. Rybak A. Solana J., 2012 Gene expression of pluripotency determinants is conserved between mammalian and planarian stem cells. EMBO J. 31: 2755–2769. 22543868 10.1038/emboj.2012.110 PMC3380209

[bib44] Oviedo, N. J., C. L. Nicolas, D. S. Adams, and M. Levin, 2008 Planarians: a versatile and powerful model system for molecular studies of regeneration, adult stem cell regulation, aging, and behavior. Cold Spring Harb. Protoc. 2008. Available at: http://cshprotocols.cshlp.org/content/2008/10/pdb.emo101.long. 10.1101/pdb.emo101 PMC10467510 21356684

[bib45] Pearson B. J. Sanchez Alvarado A., 2010 A planarian p53 homolog regulates proliferation and self-renewal in adult stem cell lineages. Development 137: 213–221. 20040488 10.1242/dev.044297 PMC2799157

[bib46] Resch A. M. Palakodeti D. Lu Y. C. Horowitz M. Graveley B. R., 2012 Transcriptome analysis reveals strain-specific and conserved stemness genes in Schmidtea mediterranea. PLoS One 7: e34447. 22496805 10.1371/journal.pone.0034447 PMC3319590

[bib47] Robb S. M. Gotting K. Ross E. Sanchez Alvarado A., 2015 SmedGD 2.0: The Schmidtea mediterranea genome database. Genesis 53: 535–546. 26138588 10.1002/dvg.22872 PMC4867232

[bib48] Rogers J. Early P. Carter C. Calame K. Bond M., 1980 Two mRNAs with different 3′ ends encode membrane-bound and secreted forms of immunoglobulin mu chain. Cell 20: 303–312. 6771019 10.1016/0092-8674(80)90616-9

[bib49] Rossi L. Salvetti A. Batistoni R. Deri P. Gremigni V., 2008 Planarians, a tale of stem cells. Cell. Mol. Life Sci. 65: 16–23. 18030424 10.1007/s00018-007-7426-y PMC11131768

[bib50] Rothberg J. M. Hinz W. Rearick T. M. Schultz J. Mileski W., 2011 An integrated semiconductor device enabling non-optical genome sequencing. Nature 475: 348–352. 21776081 10.1038/nature10242

[bib51] Rouhana L. Shibata N. Nishimura O. Agata K., 2010 Different requirements for conserved post-transcriptional regulators in planarian regeneration and stem cell maintenance. Dev. Biol. 341: 429–443. 10.1016/j.ydbio.2010.02.037 20230812

[bib52] Rouhana L. Weiss J. A. King R. S. Newmark P. A., 2014 PIWI homologs mediate histone H4 mRNA localization to planarian chromatoid bodies. Development 141: 2592–2601. 24903754 10.1242/dev.101618 PMC4067957

[bib53] Sanchez Alvarado A., 2003 The freshwater planarian Schmidtea mediterranea: embryogenesis, stem cells and regeneration. Curr. Opin. Genet. Dev. 13: 438–444. 12888018 10.1016/s0959-437x(03)00082-0

[bib54] Sandberg R. Neilson J. R. Sarma A. Sharp P. A. Burge C. B., 2008 Proliferating cells express mRNAs with shortened 3′ untranslated regions and fewer microRNA target sites. Science 320: 1643–1647. 18566288 10.1126/science.1155390 PMC2587246

[bib55] Sasidharan V. Lu Y. C. Bansal D. Dasari P. Poduval D., 2013 Identification of neoblast- and regeneration-specific miRNAs in the planarian Schmidtea mediterranea. RNA 19: 1394–1404. 23974438 10.1261/rna.038653.113 PMC3854530

[bib56] Schonemann L. Kuhn U. Martin G. Schafer P. Gruber A. R., 2014 Reconstitution of CPSF active in polyadenylation: recognition of the polyadenylation signal by WDR33. Genes Dev. 28: 2381–2393. 25301781 10.1101/gad.250985.114 PMC4215183

[bib57] Shepard P. J. Choi E. A. Lu J. Flanagan L. A. Hertel K. J., 2011 Complex and dynamic landscape of RNA polyadenylation revealed by PAS-Seq. RNA 17: 761–772. 21343387 10.1261/rna.2581711 PMC3062186

[bib58] Shi Y., 2012 Alternative polyadenylation: new insights from global analyses. RNA 18: 2105–2117. 23097429 10.1261/rna.035899.112 PMC3504663

[bib59] Shin C. Nam J.-W. Farh K. K.-H. Chiang H. R. Shkumatava A., 2010 Expanding the microRNA targeting code: functional sites with centered pairing. Mol. Cell 38: 789–802. 20620952 10.1016/j.molcel.2010.06.005 PMC2942757

[bib60] Sievers F. Wilm A. Dineen D. Gibson T. J. Karplus K., 2011 Fast, scalable generation of high-quality protein multiple sequence alignments using Clustal Omega. Mol. Syst. Biol. 7: 539. 21988835 10.1038/msb.2011.75 PMC3261699

[bib61] Singh P. Alley T. L. Wright S. M. Kamdar S. Schott W., 2009 Global changes in processing of mRNA 3′ untranslated regions characterize clinically distinct cancer subtypes. Cancer Res. 69: 9422–9430. 19934316 10.1158/0008-5472.CAN-09-2236 PMC2794997

[bib62] Smibert P. Miura P. Westholm J. O. Shenker S. May G., 2012 Global patterns of tissue-specific alternative polyadenylation in Drosophila. Cell Reports 1: 277–289. 22685694 10.1016/j.celrep.2012.01.001 PMC3368434

[bib63] Solana J. Gamberi C. Mihaylova Y. Grosswendt S. Chen C., 2013 The CCR4-NOT complex mediates deadenylation and degradation of stem cell mRNAs and promotes planarian stem cell differentiation. PLoS Genet. 9: e1004003. 24367277 10.1371/journal.pgen.1004003 PMC3868585

[bib64] Sun Y. Fu Y. Li Y. Xu A., 2012 Genome-wide alternative polyadenylation in animals: insights from high-throughput technologies. J. Mol. Cell Biol. 4: 352–361. 23099521 10.1093/jmcb/mjs041

[bib65] Tian B. Hu J. Zhang H. Lutz C. S., 2005 A large-scale analysis of mRNA polyadenylation of human and mouse genes. Nucleic Acids Res. 33: 201–212. 15647503 10.1093/nar/gki158 PMC546146

[bib66] Ulitsky I. Shkumatava A. Jan C. H. Sive H. Bartel D. P., 2011 Conserved function of lincRNAs in vertebrate embryonic development despite rapid sequence evolution. Cell 147: 1537–1550. 22196729 10.1016/j.cell.2011.11.055 PMC3376356

[bib67] Westesson O. Skinner M. Holmes I., 2013 Visualizing next-generation sequencing data with JBrowse. Brief. Bioinform. 14: 172–177. 22411711 10.1093/bib/bbr078 PMC3603211

[bib68] You L. Wu J. Feng Y. Fu Y. Guo Y., 2015 Apasdb: a database describing alternative poly(a) sites and selection of heterogeneous cleavage sites downstream of poly(a) signals. Nucleic Acids Res. 43: D59–D67. 25378337 10.1093/nar/gku1076 PMC4383914

[bib69] Zeng A. Li Y. Q. Wang C. Han X. S. Li G., 2013 Heterochromatin protein 1 promotes self-renewal and triggers regenerative proliferation in adult stem cells. J. Cell Biol. 201: 409–425. 23629965 10.1083/jcb.201207172 PMC3639387

[bib70] Zhao J. Hyman L. Moore C., 1999 Formation of mRNA 3′ ends in eukaryotes: mechanism, regulation, and interrelationships with other steps in mRNA synthesis. Microbiol. Mol. Biol. Rev. 63: 405–445. 10357856 10.1128/mmbr.63.2.405-445.1999 PMC98971

