# Supplementary material for: Genome-Wide Analysis of Polyadenylation Events in Schmidtea mediterranea
Source: G3 (Bethesda). 2016 Aug 2;6(10):3035–48. doi: 10.1534/g3.116.031120 (PMC5068929; doi:10.1534/g3.116.031120)
Supplement: Supplemental Material [file supp_6_10_3035__index.html]

Genome-Wide Analysis of Polyadenylation Events in Schmidtea mediterranea — Genome-Wide Analysis of Polyadenylation Events in Schmidtea mediterranea — Supplemental Material 

# Genome-Wide Analysis of Polyadenylation Events in *Schmidtea mediterranea*

## Supplemental Material for Lakshmanan, *et al*, 2016

**Files in this Data Supplement:**

- File S1 - This file contains all supplemental figures legends for supplemental tables. (.pdf, 5.63 MB)
- Figure S9 - Completing gene models for planarian transcriptomes. (.pdf, 152 KB)
- Figure S10 - 3ï¿½UTR association with the non-redundant planarian transcriptome. (.pdf, 135 KB)
- Figure S11 - Correlation of PAS with transcript level (FPKM). (.pdf, 114 KB)
- Figure S12 - Validation of 3ï¿½UTRs using RNA-Seq data. (.pdf, 263 KB)
- Figure S13 - GO enrichment for two-3P peak & coding region polyadenylation (crApA) candidates. (.pdf, 647 KB)
- Figure S14 - Control dataset for miRNA binding site distribution across two 3P peak candidates. (.pdf, 610 KB)
- Figure S15 - Lift over of polyadenylation site coordinates from SmedSxl\_V3.1 to SmedSxl\_V4.0. (.pdf, 157 KB)
- Figure S1 - CFIm59 (CPSF7) in *Schmidtea mediterranea*. (.pdf, 332 KB)
- Figure S2 - Gene tree for putative *Schmidtea* Fip1. (.pdf, 420 KB)
- Figure S3 - Gene tree for *Schmidtea* CFIm68 (CPSF6). (.pdf, 476 KB)
- Figure S4 - Sequence divergence of putative *Schmidtea* Pcf11. (.pdf, 1.2 MB)
- Figure S5 - Identification of polyadenylation/cleavage sites from SmedSxl.V3.1 genome. (.pdf, 153 KB)
- Figure S6 - Identification of conserved PolyA signal (PAS). (.pdf, 194 KB)
- Figure S7 - Retained PolyA signals (PAS) in different 3P-Tags cutoff. (.pdf, 180 KB)
- Figure S8 - Distribution of PolyA signals for 3P-peaks that are in close proximity. (.pdf, 310 KB)
- Table S1 - Polyadenylation sites identified from this study. (.xls, 15.38 MB)
- Table S2 - 3P-seq based polyadenylation site association to known transcript models. (.xls, 20.82 MB)
- Table S3 - Gene clusters obtained from CD-HIT. (.xls, 4.16 MB)
- Table S4 - Functional Enrichment analyses for alternatively polyadenylated transcripts. (.xls, 134 KB)
- Table S5 - Transcripts reported to be alternatively polyadenylated in other organisms. (.xls, 42 KB)
- Table S6 - Degradome tags from sexual and asexual genome. (.xls, 7.24 KB)
- Table S7 - Coding region polyadenylation candidates that exhibited domain loss. (.xls, 117 KB)
- Table S8 - Liftover of polyadenylation site coordinates from SxlV3.1 to SxlV4 genome. (.xls, 3.69 MB)
- Table S9 - Primers for the RT-PCR and WISH used in this study. (.xls, 30 KB)
- Table S10 - X1 enriched Alternatively polyadenylated transcripts. (.xls, 746 KB)
