# Supplementary material for: Genome-Wide Analysis of Polyadenylation Events in Schmidtea mediterranea
Source: G3 (Bethesda). 2016 Aug 2;6(10):3035–48. doi: 10.1534/g3.116.031120 (PMC5068929; doi:10.1534/g3.116.031120)
Supplement: Supplemental Material [file supp_g3.116.031120_FileS1.pdf]

Supplemental Material

Genome-wide analysis of polyadenylation events in *Schmidtea mediterranea*

Vairavan Lakshmanan[1, 2#], Dhiru Bansal[1, 3#], Jahnavi Kulkarni[1], Deepak Poduval[1], Srikar Krishna[1, 2], Vidyanand Sasidharan[1, 3], Praveen Anand [1], Aswin Seshasayee[4]* and Dasaradhi Palakodeti[1]**

Supplemental tables:

Supplemental Tables

Table S1: Polyadenylation sites identified from this study. This table contains all the information on the identified cleavage/polyadenylation site from the computational pipeline described in the manuscript. It reports the cleavage/polyadenylation site from all the three currently available smed genomes - SmedSxlV3.1, SmedSxlV4 and AsxlV1. The 3P-Peak sequence identified along with the PAS is given in this table.

Table S2: 3P-seq based polyadenylation site association to known transcript models. Transcripts from three different transcriptome (mk4, OX-smed_v1, dd_smed_v4), along with the associated cleavage site are reported. The number of 3P-tags associated with the cleavage site is also reported.

Table S3: Gene clusters obtained from CD-HIT. All the clusters obtained from CD-HIT with clustering threshold of 90% sequence identity. The information on the association of the 3P-peak for the clustered transcript is also reported in Supplemental Table 2 (sheet2).

Table S4: Functional Enrichment analyses for alternatively polyadenylated transcripts. The REVIGO of GO enrichment analysis for the transcripts associated with multiple polyadenylation sites (Sheet 1) and coding region polyadenylation (Sheet 2).

Table S5: **Transcripts reported to be alternatively polyadenylated in other organisms.** The corresponding homolog identified in planaria along with its polyadenylation status is reported in this table.

Table S6: Degradome tags from sexual and asexual genome. The degradome tags that correspond to miRNA from degradome sequencing are given here. The table contains degradome tag id and the corresponding miRNA for that tag along with the alignment of miRNA to the degradome tag.

Table S7: Coding region polyadenylation candidates that exhibited domain loss. Coding region polyadenylation candidates (crApA), which showed putative domain loss.

Table S8: Liftover of polyadenylation site coordinates from SxlV3.1 to SxlV4 genome. The overlap of polyadenylation sites derived from SxlV3.1 genome and SxlV4 genome. The lift-over of coordinates at 85% (Sheetl) and 50% (Sheet2) overlap between the annotated regions is given.

Table S9: Primers for the RT-PCR and WISH used in this study.

Table S10: X1 enriched Alternatively polyadenylated transcripts. A blast search was performed by using all the transcripts that have more than one polyadenylation site (5644) against Isotigs transcriptome (Onal P *et al* ,2012) (parameters: evalue 1e-05, max_target_seqs 1). This resulted in identification of 4412 corresponding isotigs with more than 50% coverage and 95% sequence identity. Out of these 1606 transcripts were atleast two-fold up regulated in X1 in comparison to Xins population. The table consist of blast results and fold change values for these 1606 transcripts.

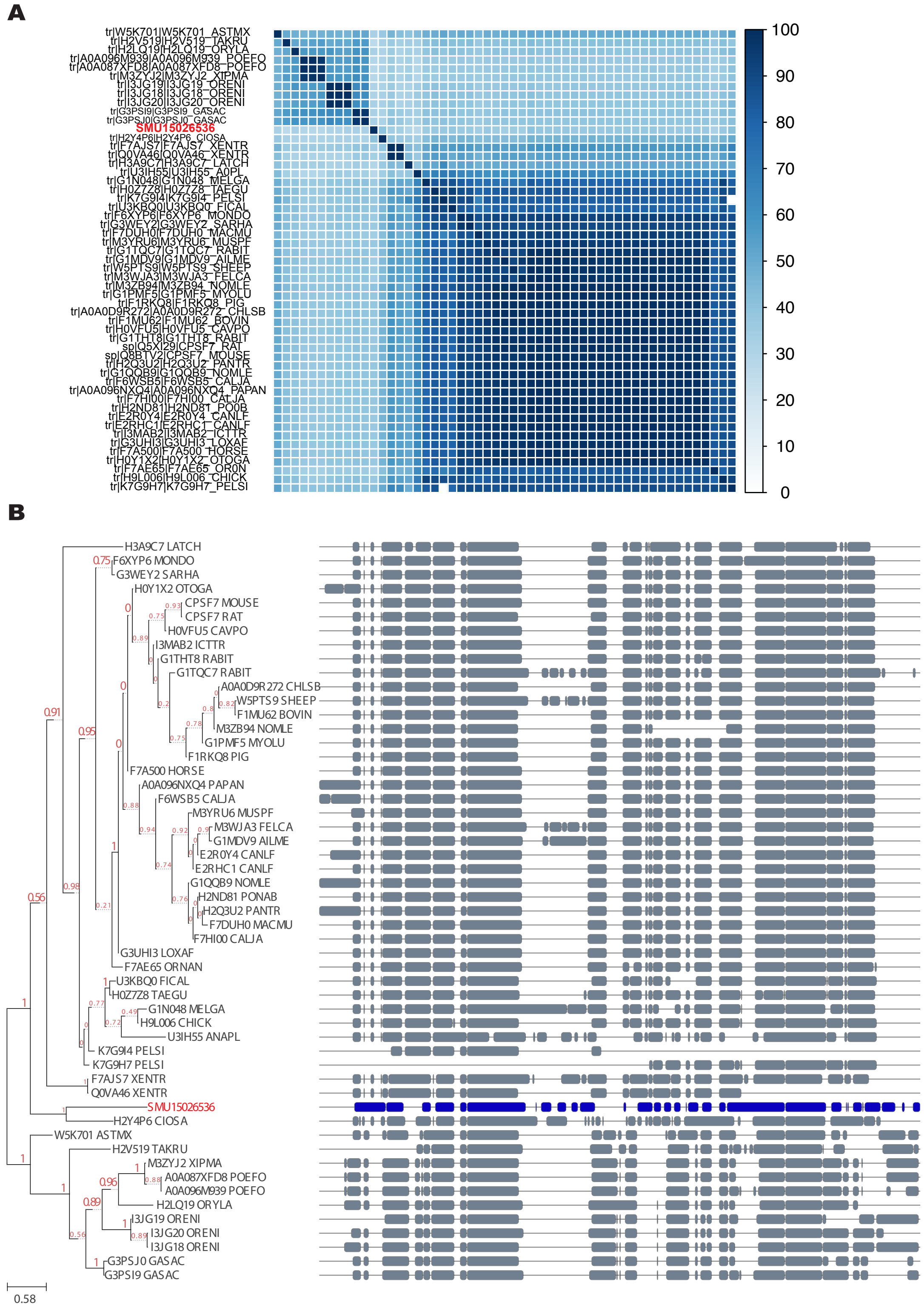

Figure S1. CFIm59 (CPSF7) in *Schmidtea mediterranea*. A) The percentage sequence identity matrix derived from *clustal omega* for CFIm59, a component of cleavage factor-Im59 complex. **B**) Single gene tree plotted for planarian CFIm59 with other organisms CFIm59 using ***ete-build***. Left panel of the plot shows phylogenetic tree constructed based on alignment blocks shown on right. Planarian CPSF7 sequence stands out as an outlier in

comparison to other CFIm59 proteins, suggesting the high variation in the sequence that could exist for this protein within planaria.

A

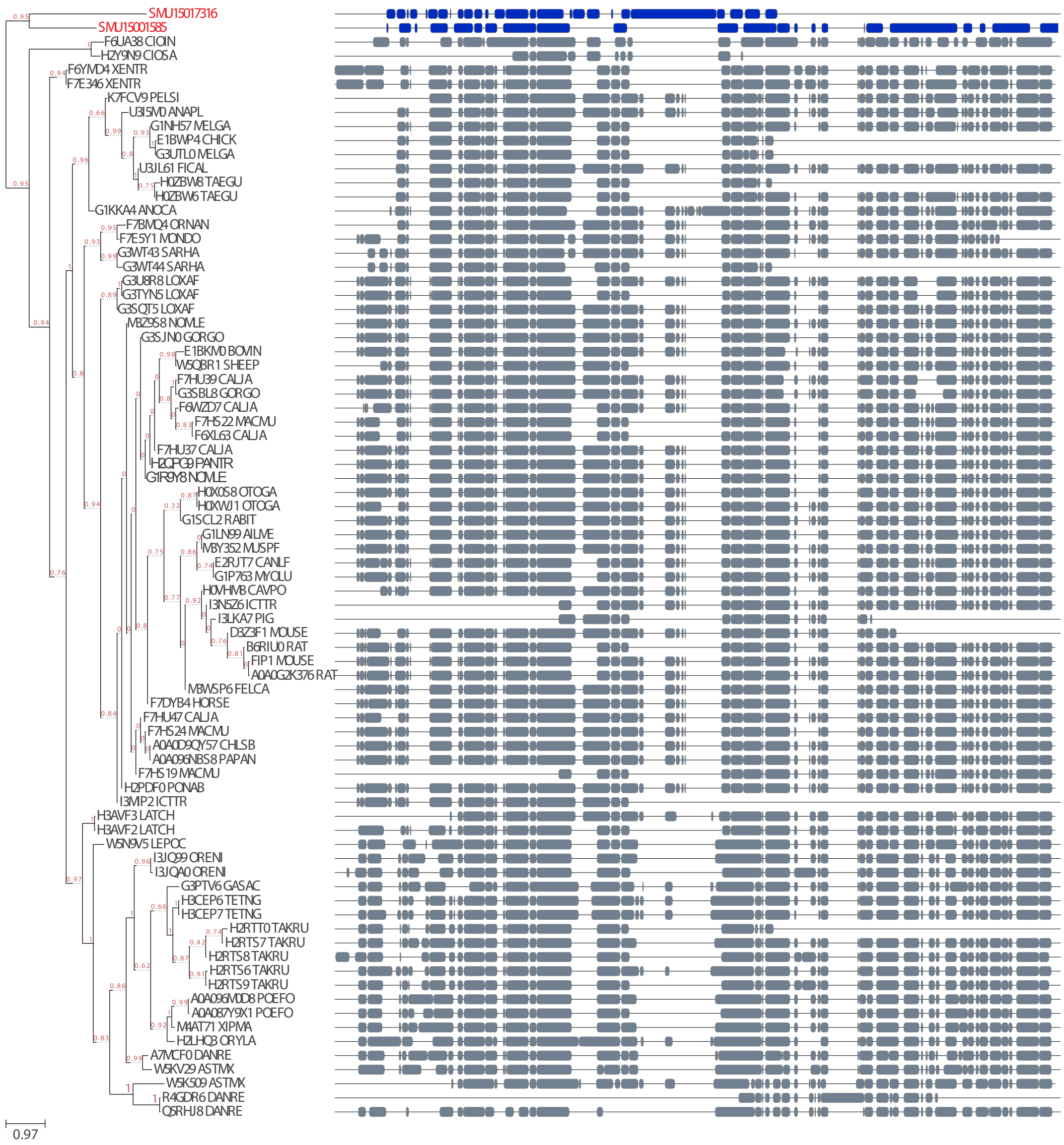

Figure S2. Gene tree for putative *Schmidtea* Fip1. A) Single gene based phylogeny plotted for planarian Fip1, a subunit from cleavage and polyadenylation machinery along with Fip1 gene from other organisms using ***ete-build***. Planarian Fip1 sequence clusters outside all known Fip1 genes and has very poor % sequence identity with other known Fip1 sequences (data not shown). This suggests that currently derived planarian Fip1 sequence is highly variable in comparison to Fip1 from other organisms.

A

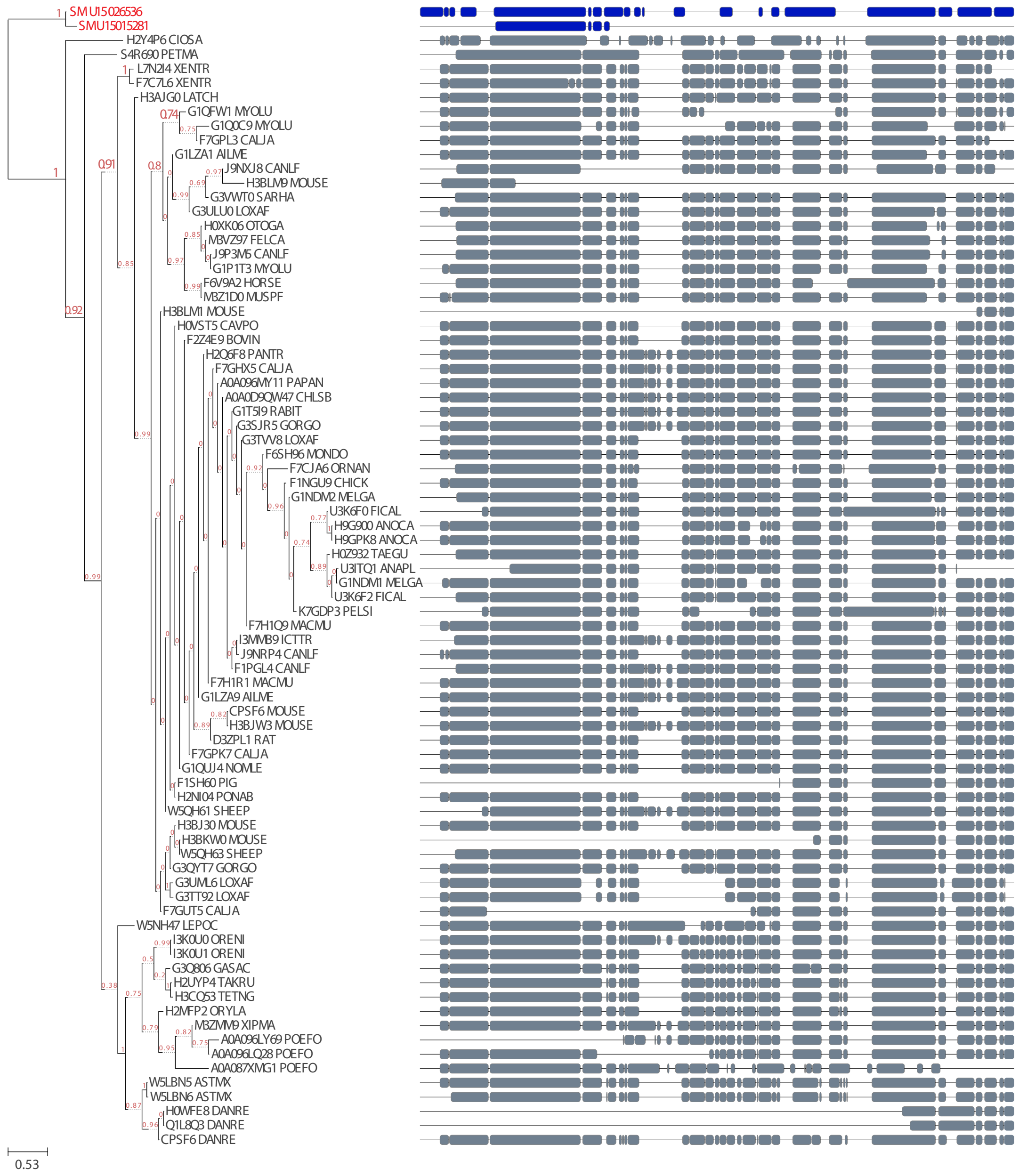

Figure S3. Gene tree for *Schmidtea* CFIm68 (CPSF6). A) Single gene based phylogeny plotted for planarian CPSF6, a component of cleavage factor-Im68 complex with CPSF6 gene from other organisms using ***ete-build***. Planarian CPSF6 sequence clusters outside all known CPSF6 genes and has very poor % sequence identity with other known CPSF6 sequences (data not shown). This suggests that currently predicted planarian CPSF6 from recent genome has high sequence variation in comparison with CPSF6 from other organisms.

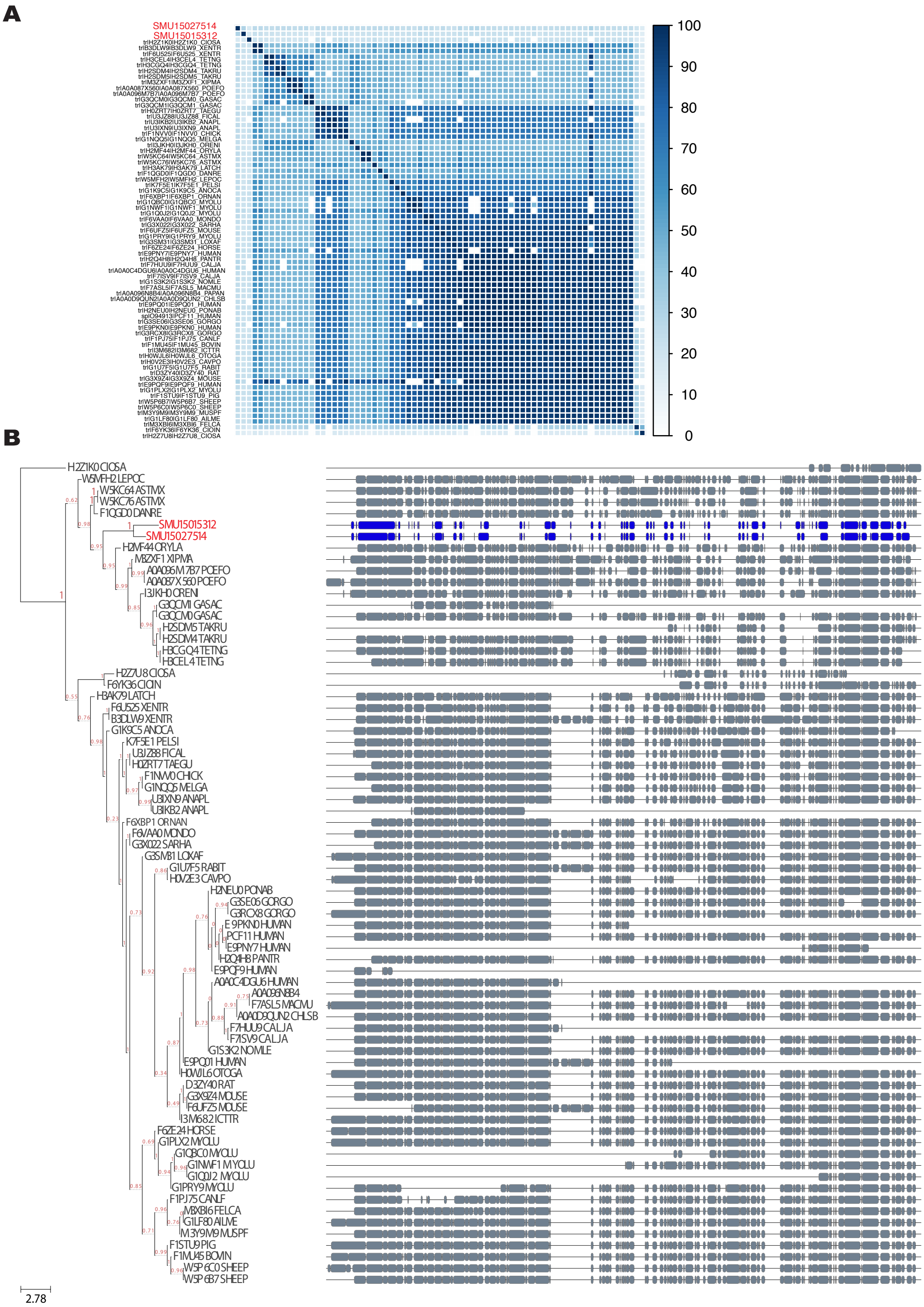

Figure S4. Sequence divergence of putative *Schmidtea* Pcf11. A) The percentage sequence identity matrix from multiple sequence alignment derived using *clustal omega* for Pcf11, a subunit from cleavage and polyadenylation factor. The highlighted planarian Pcf11 % sequence identity with known Pcf11 genes from other organisms is <10%. **B**) Single gene based phylogeny plotted for planarian Pcf11 with other organisms Pcf11 using ***ete-build***. Left side of the plot shows phylogenetic tree constructed based on alignment blocks shown in right.

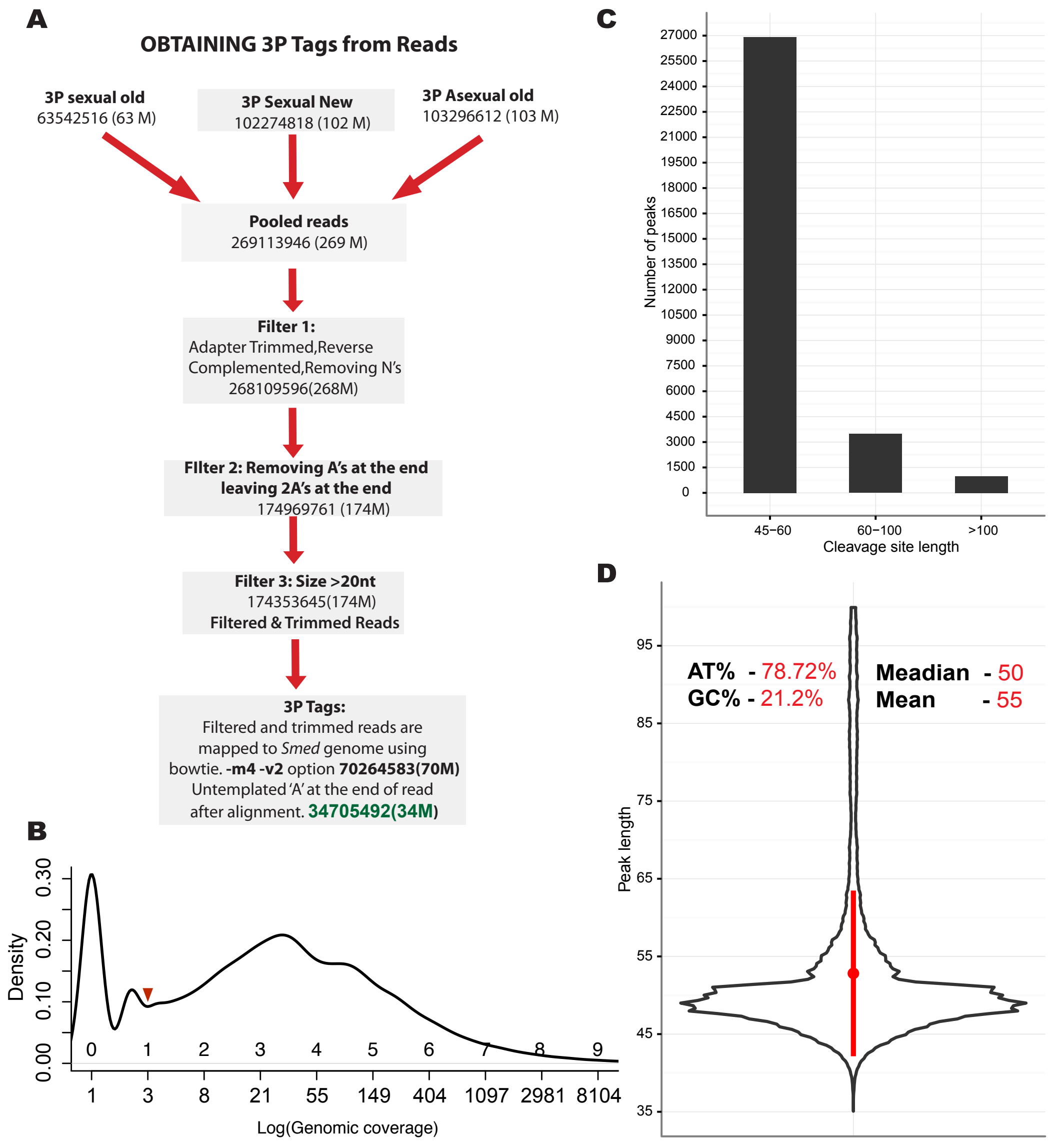

Figure S5. Identification of polyadenylation/cleavage sites from SmedSxl.V3.1 genome. **A**) The computational pipeline to obtain 3P tags from 3P-seq reads. The workflow explains all the pre-processing steps to obtain 3P-tags. **B**) Density distribution of reads per locus indicating the genomic coverage of 3P-tags. The x-axis denotes the genomic coverage in normal (below) & log scale (above), whereas the y-axis represents the density of number of locus. **C)** Binned histogram representing the length of 3P-peaks. Majority of the 3P-Peaks are 45-60nts in length. **D**) Violin plot representing the length distribution of identified cleavage/polyadenylation peaks (3P-Peaks) from our computational pipeline. The mean and median of the distribution is 50 nts & 55nts respectively. As expected, identified cleavage sites are AT rich suggesting that these are from 3' end of the transcript.

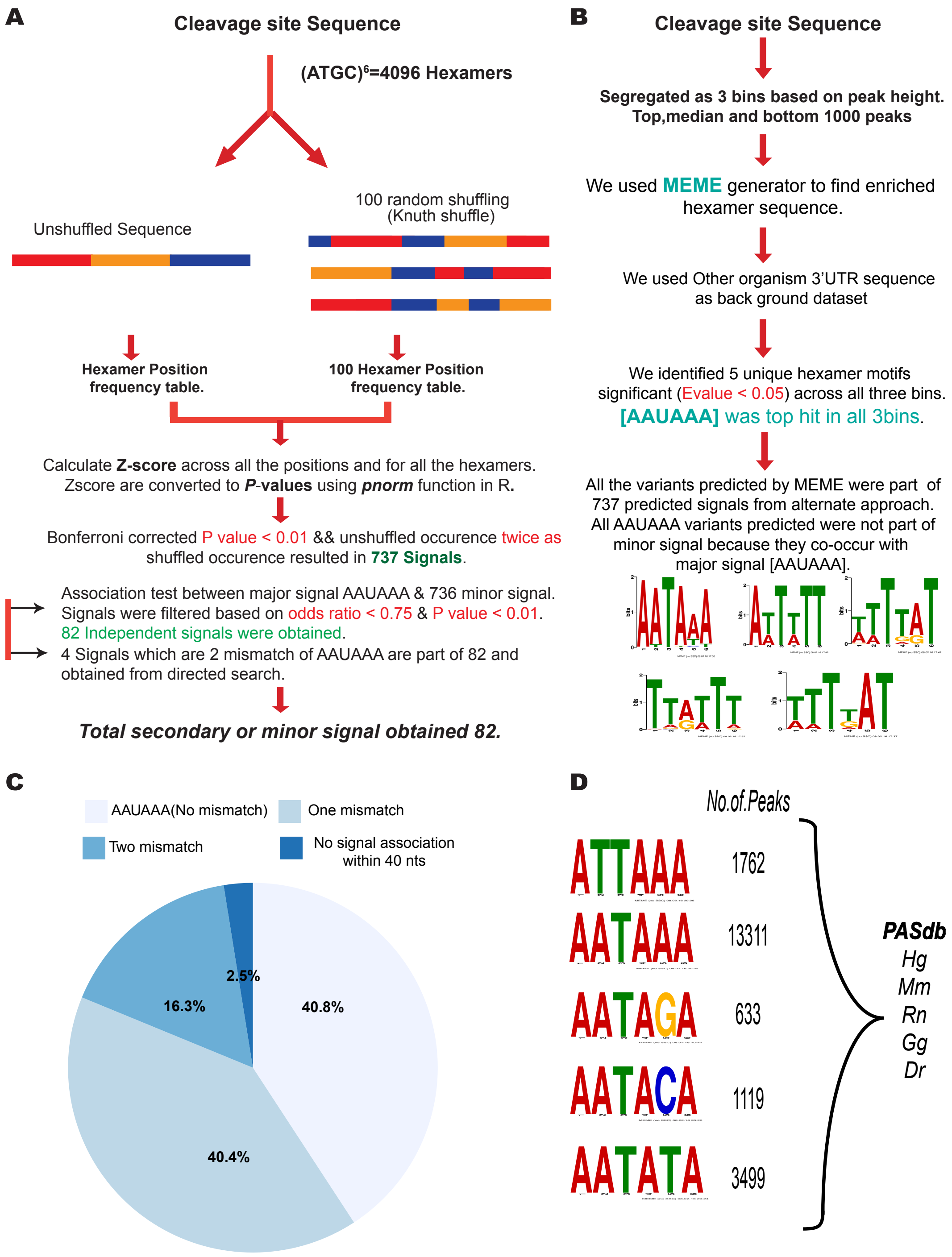

Figure S6. Identification of conserved PolyA signal (PAS). A) Schematic describing the computational pipeline to identify PAS signal. Identification of major and minor/secondary signal by fisher's exact test & directed PAS search. **B**) Alternate pipeline used to identify PAS from identified cleavage site based on peak height. **C**) Association of PAS to identified cleavage sites. Venn diagram depicting the percentage of identified cleavage sites that have AAUAAA or single nucleotide variant of AAUAAA at 10-40nt upstream of cleavage sites. **D**) Overlap of the identified PAS from *Schmidtea* with PolyA signal database.

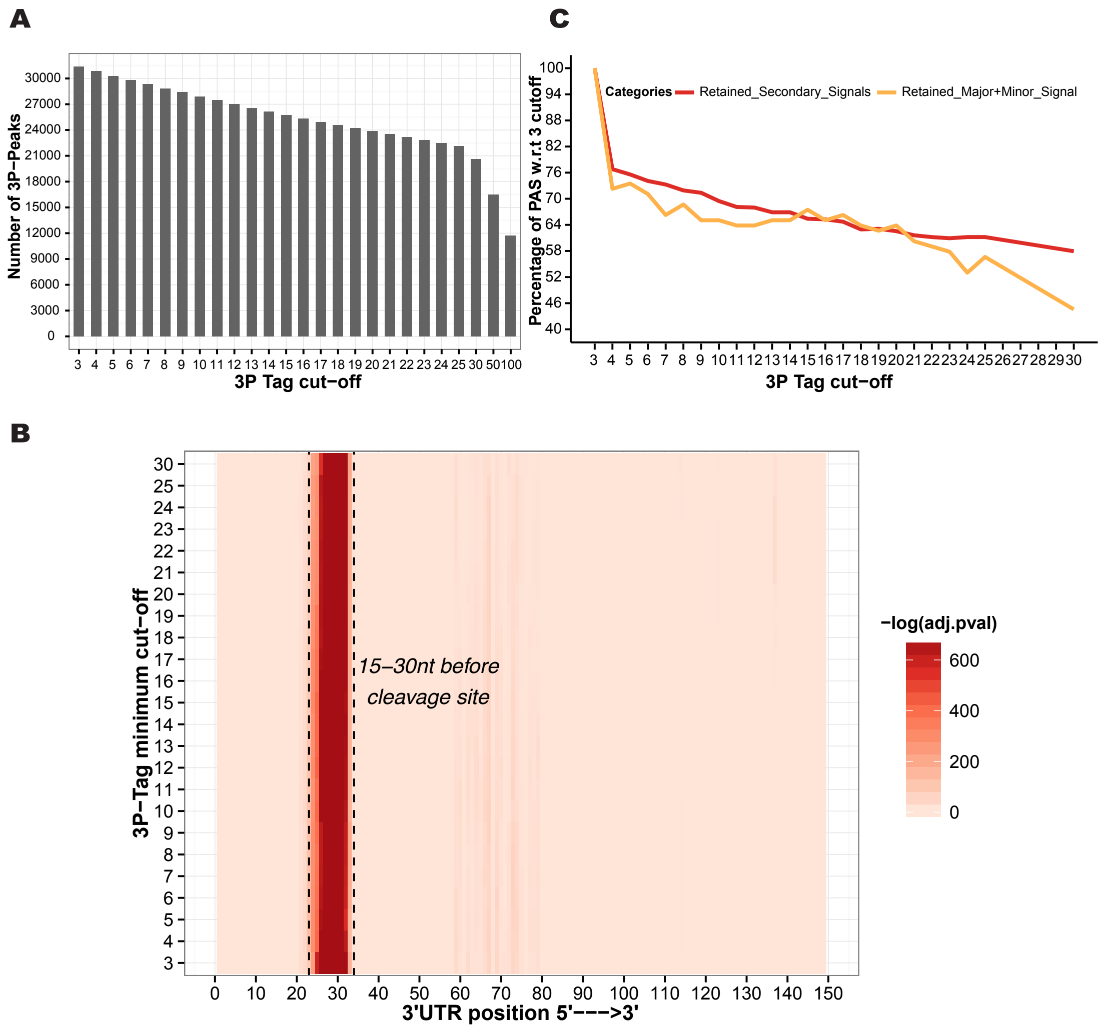

Figure S7. Retained PolyA signals (PAS) in different 3P-Tags cutoff. A) Bar plot depicting total number of identified cleavage sites (3P-Peaks) at different tag cut-off. **B**) Adjusted *Pvalue* obtained from hexamer enrichment analysis (as described before) for the major signal AAUAAA across 3P-peaks obtained from different 3P tag cutoff. **C**) Overlap percentage of enriched PAS and major+minor signal obtained from three 3P tag cutoff used in this study with other 3P tag cutoffs (4-30).

A

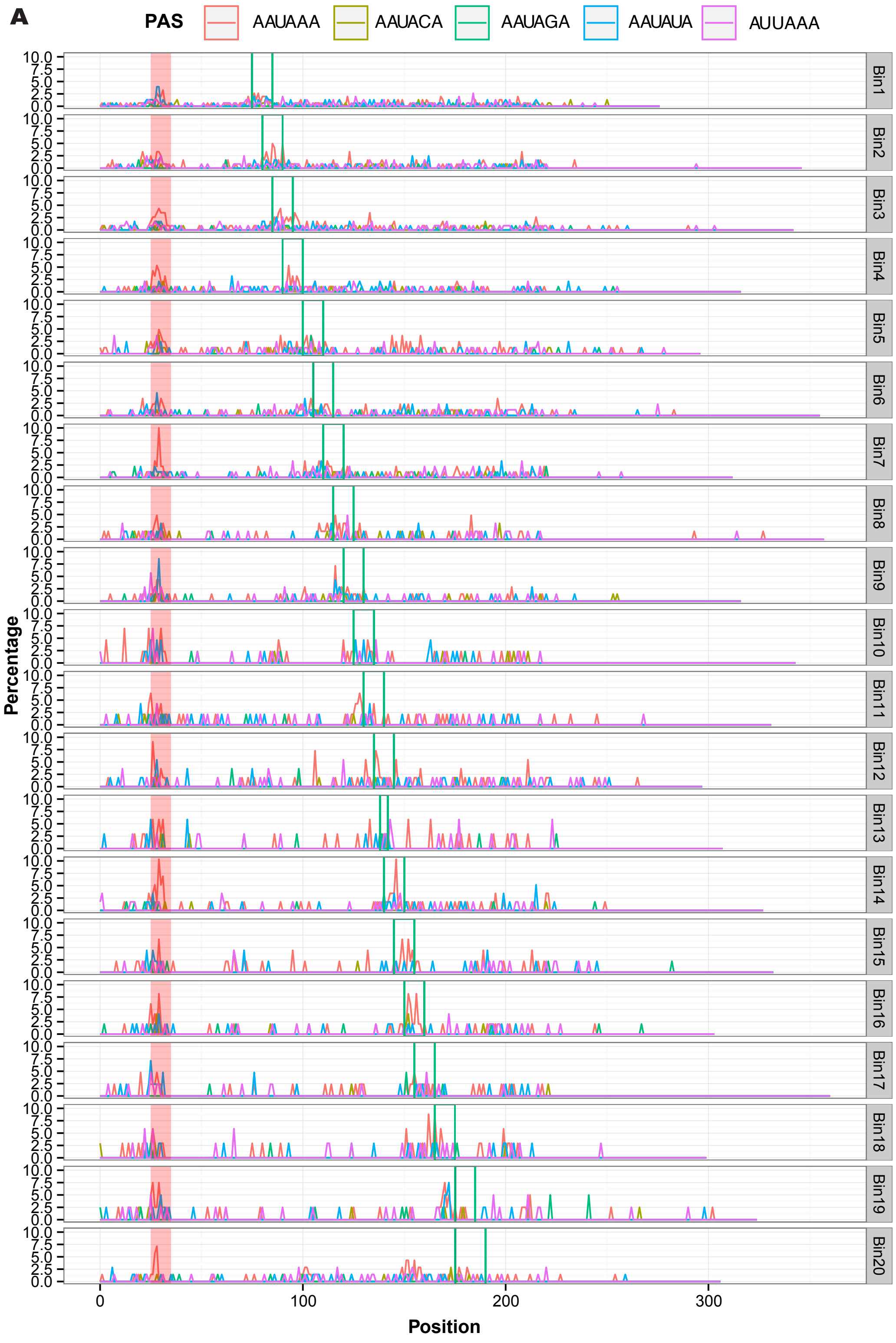
PAS
AAUAAA
AAUACA
AAUAGA
AAUAUA
AUUAAA
Percentage
10.0
7.5
5.0
2.5
0.0
Bin1
Bin2
Bin3
Bin4
Bin5
Bin6
Bin7
Bin8
Bin9
Bin10
Bin11
Bin12
Bin13
Bin14
Bin15
Bin16
Bin17
Bin18
Bin19
Bin20
0
100
200
300
Position

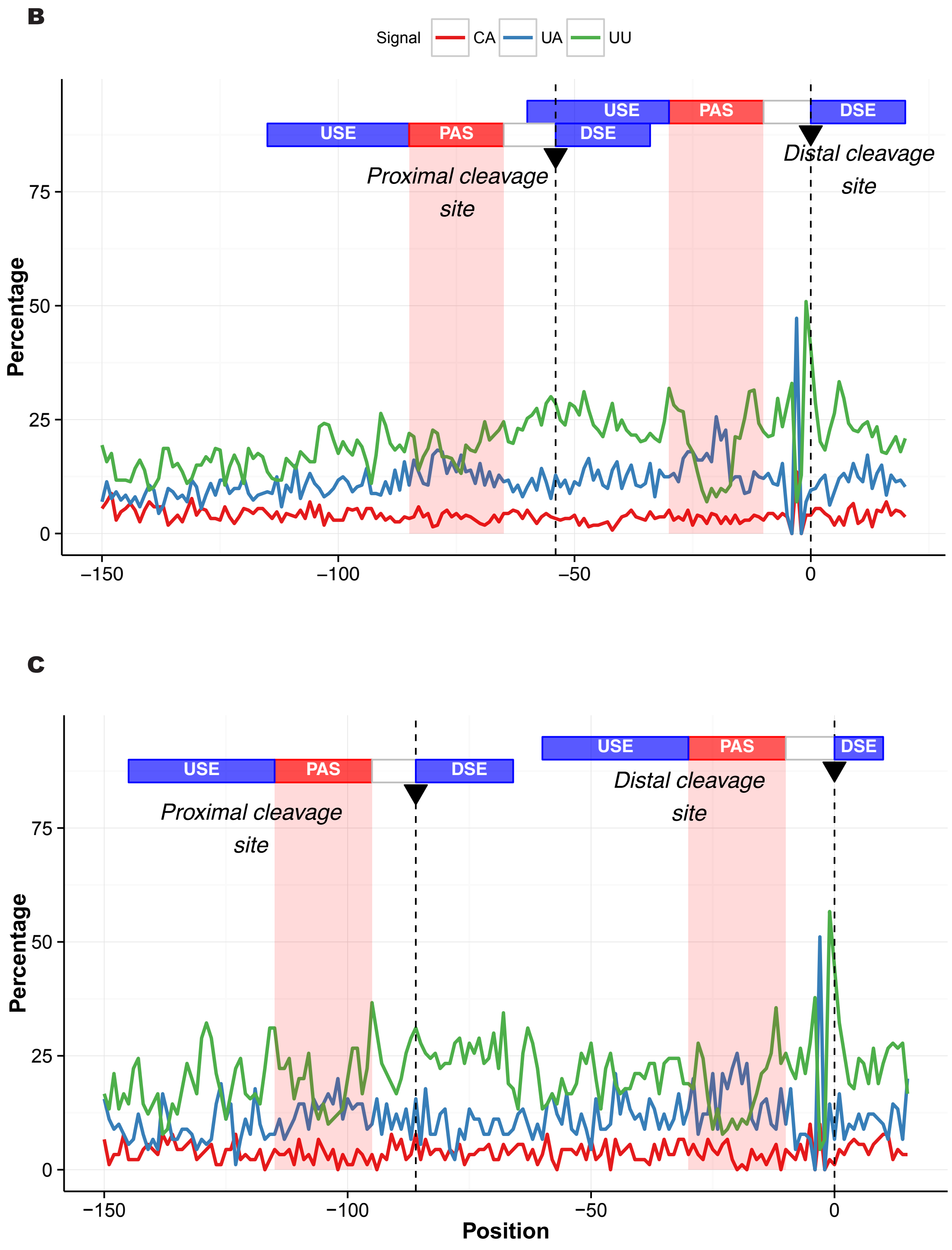

Figure S8. Distribution of PolyA signals for 3P-peaks that are in close proximity. **A)** Polyadenylation sites that are in close proximity to each other in genome (within 100nt) are binned with 5nt interval. We scanned for the presence of five enriched PAS signal in the region spanning 100nts on either side of the two-cleavage site that are close to each other. We observed that polyadenylation sites that are as close as 5nt (bin1) have distinct PAS associated at the distance of 10-30nt upstream suggesting these are independent polyadenylation sites. Highlighted region shows PAS signal in proximal 3P-Peak and boxed

region shows PAS signal in distal 3P-Peak. **B**) A line-plot showing the dinucleotide profile(CA, UU, UA) of all the 3P-peaks that are separated < 10nts. The 0-position on the x-axis denotes the cleavage site (annotated with dotted line and arrow). The upstream elements (USE) and the downstream elements (DSE), along with the polyadenylation signal (PAS) have been separately highlighted in blue and red respectively. **C**) A line plot similar to (**B**) showing dinucleotide profile of all the 3P peaks that are separated by 30-35nts.

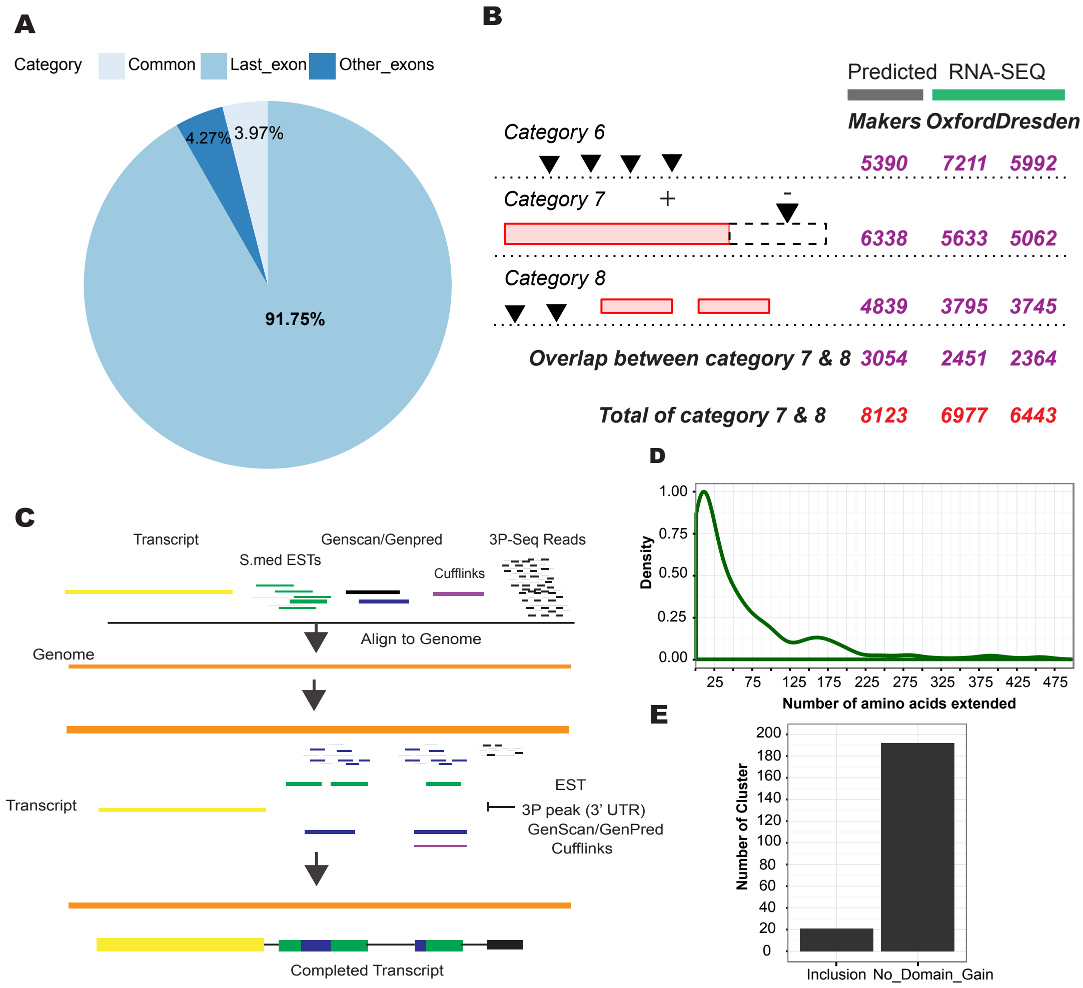

Figure S9. Completing gene models for planarian transcriptomes. A) The pie chart depicts the percentage distribution of polyadenylation sites across different exons. **B**) 3P-peaks that were present on the genomic contigs with no transcript annotation(Category 6). Number of polyadenylation sites that are in opposite orientation to the transcript (Category 7) & polyadenylation sites that cannot be associated with specific transcripts (Category 8). **C**) The schematic explains the computational workflow used to stitch the annotated 3'UTR to known transcripts. **D**) The density distribution of number of amino acids extended after stitching the known transcript model. **E**) The number of transcripts that had additional protein domain in the extended region of the transcript.

A

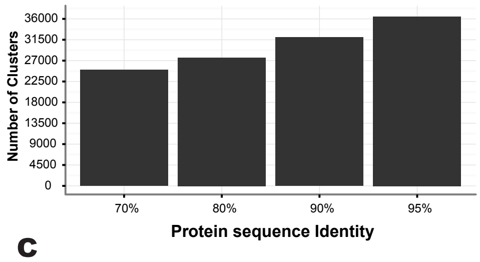

B

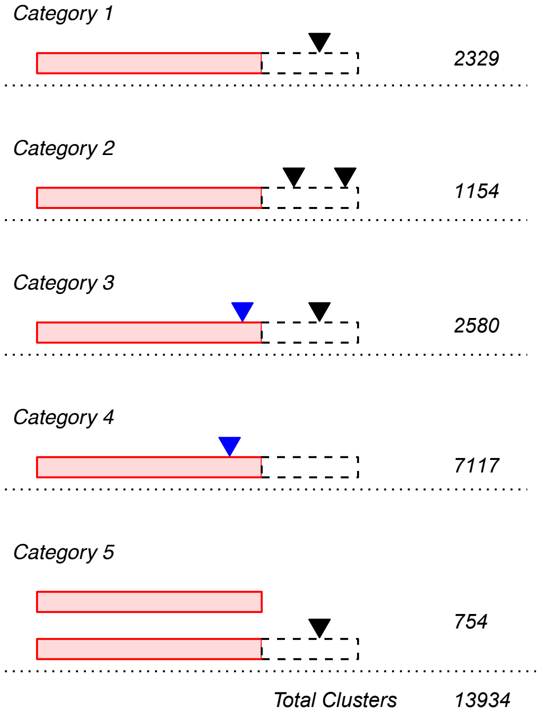

C

	Number of Clusters	Percentage
One Peak	8920	59.5
Two Peak	3550	25.5
Three Peak	1322	9.5
>=4 Peaks	772	5.5

D

	Number of Clusters
Annotated	7117
With Evidence	2647
No Evidence	4170

E

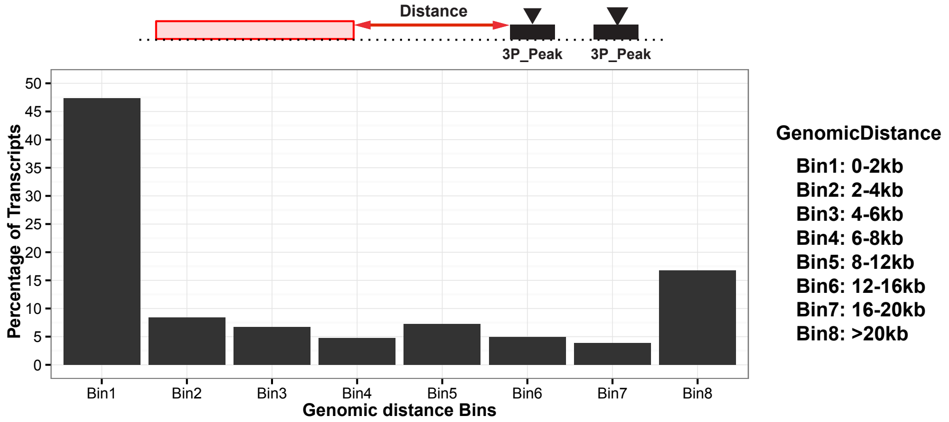

Figure S10. 3'UTR association with the non-redundant planarian transcriptome. A) The barplot represents the number of clusters obtained from CD-HIT using different % sequence identity thresholds. **B)** The distribution of derived gene clusters across different transcript association categories. **C)** The number of transcripts (gene clusters) associated with different polyadenylation sites. Around 40% of transcripts associate with more than one polyadenylation sites. **D)** **N**umber of gene clusters that could be extended further based upon the 3P-Peaks associated with them. **E)** Genomic distance of nearest polyadenylation site found from the known transcript models are binned. Around 48% of gene models are associated with polyadenylation/cleavage site within 2kb downstream.

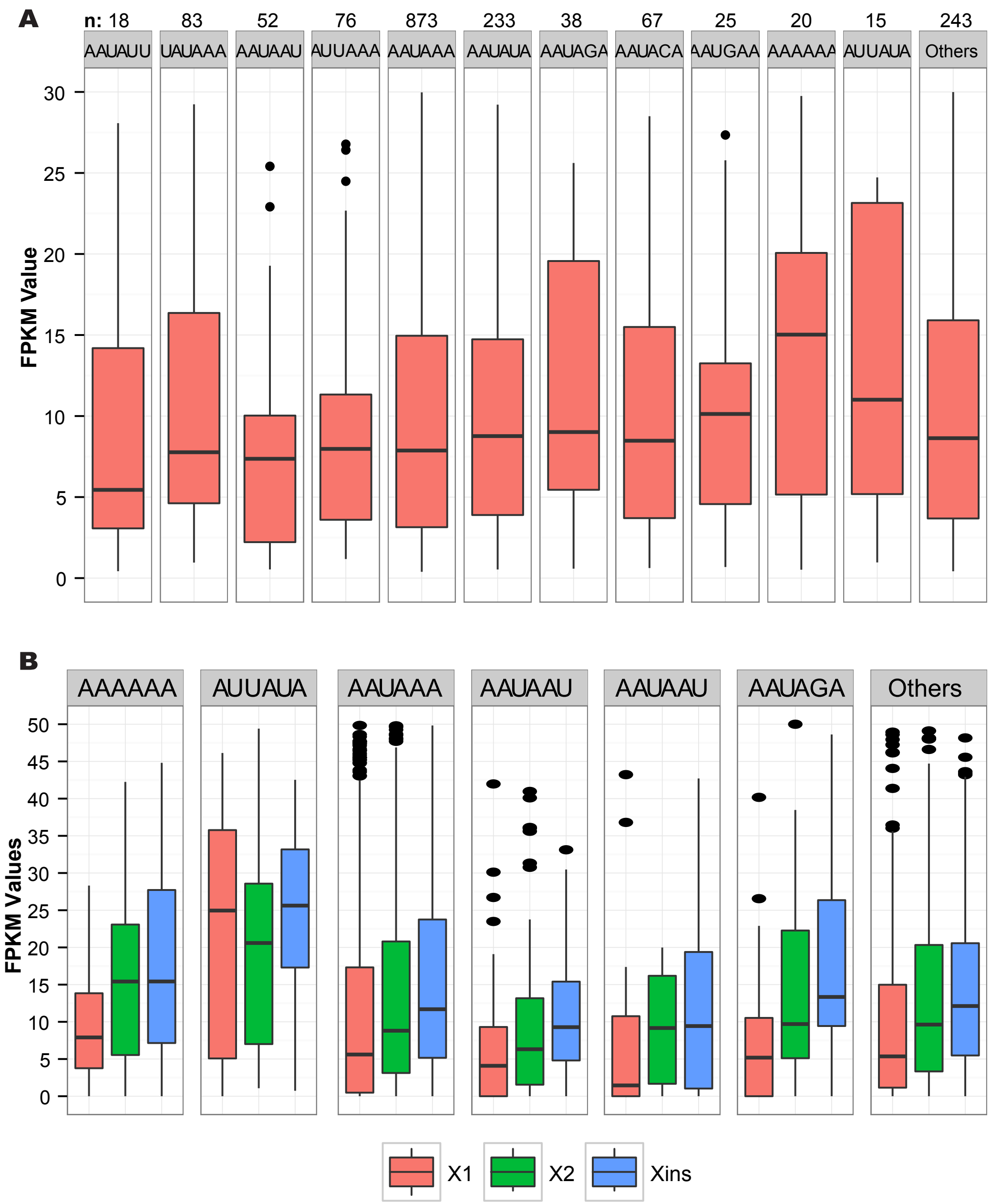

Figure S11. Correlation of PAS with transcript level (FPKM). A) The box plot depicts FPKM distribution of transcripts having top 12 polyadenylation signals. Transcripts with one annotated 3'UTR is considered for analysis. **B**) Transcripts with particular PAS, which showed distinct FPKM distribution profile are chosen and looked for cell-type specific FPKM distribution.

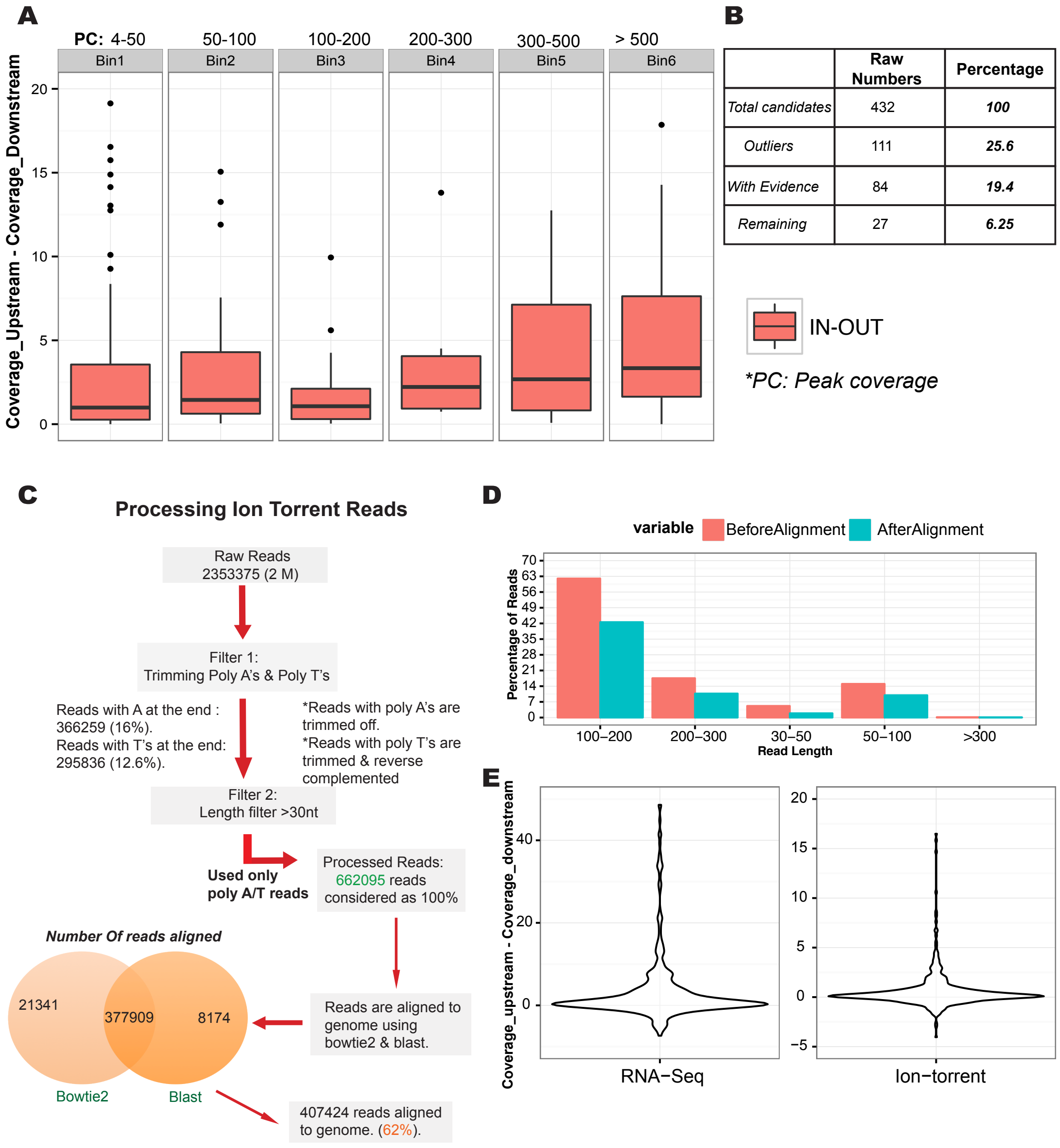

Figure S12. Validation of 3'UTRs using RNA-Seq data. A) RNASeq coverage for upstream region (from transcript end to polyadenylation site start) and downstream region (after polyadenylation site) across different bins of polyadenylation site coverage (PC). The difference in the RNA-Seq coverage values in the upstream and the downstream region increases with the increase in PC. **B**) Table depicting raw number and percentage of transcripts used in validation of 3P-Seq using RNA-Seq data analysis. **C**) The schematic describing the different steps of pre-processing for reads obtained from Ion torrent platform. **D**) The binned-frequency of the Ion-torrent read lengths. More than 80% of reads have > 100nts. **E**) A violin plot depicting the difference in the coverage between the upstream and downstream region derived from RNA-Seq and Ion-Torrent reads.

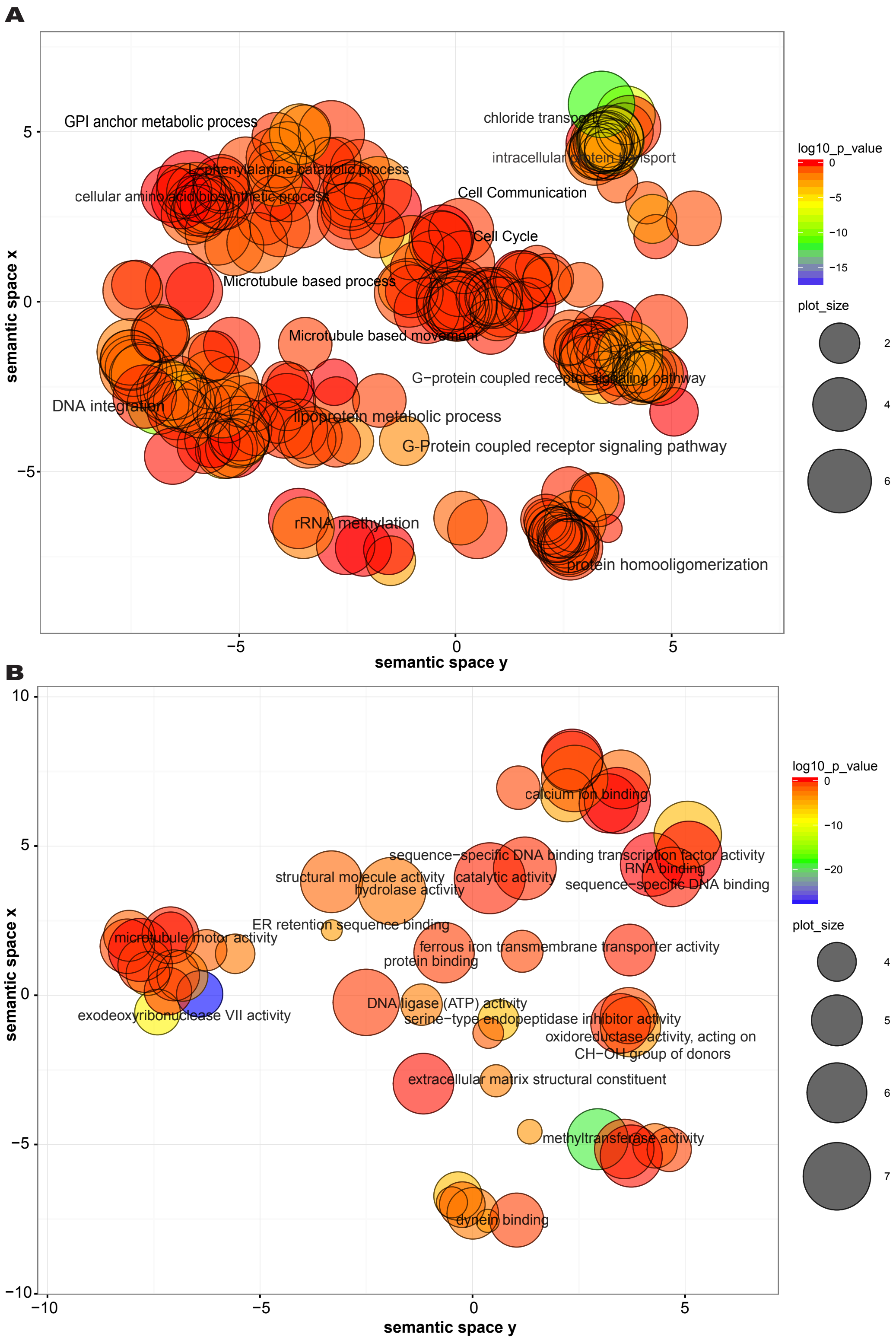

Figure S13. GO enrichment for two-3P peak & coding region polyadenylation (crApA) candidates. A) Scatterplot derived from REVIGO analysis depicting the clusters of GO terms (Biological Process) associated with the transcripts having multiple polyadenylation sites. Each circle represents a GO-term, the color reflects the significance of enrichment and the size denotes the abundance of that particular GO-term in the background uniprot dataset. **B**)

Scatterplot derived from REVIGO analysis depicting the clusters of GO terms associated with polyadenylation event that could lead to putative loss of protein domains.

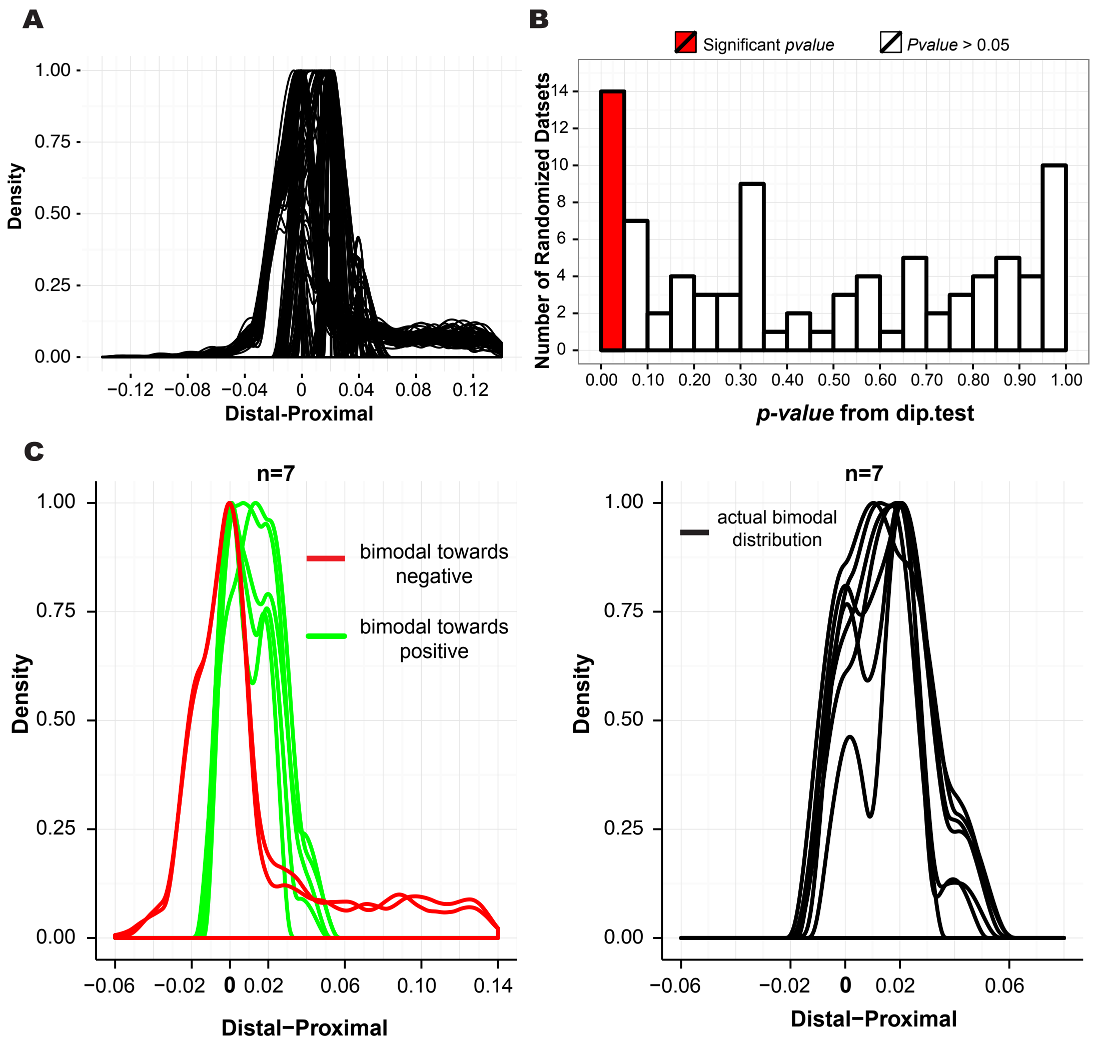

Figure S14. Control dataset for miRNA binding site distribution across two 3P peak candidates. A) Density distribution of difference between number of miRNA binding site at proximal & distal 3P-peak of randomized control dataset (explained in methods). **B**) We performed Hartigans' dip test for multi-modality of the randomized 100 datasets to see how many of the randomized dataset follow multi-modal distribution. Histogram of binned *P-values* from Hartigans' dip test is plotted. **C**) This shows that 14 out of 101 randomized dataset show significant *P-value* from dip test (suggesting bimodal distribution). Only seven (right panel) of the 14 datasets had the same bimodal profile as seen in actual dataset.

A

	Smed_SxlV4	Smed_ASxlV1
Total Reads	90416419	83937226
Aligned Reads	33841718	31539047
3P Tags	15983576	16557733
3P Peaks	29836	11998
Enriched Signal	607	409
Minor Signals	58	34
Peaks with AAUAAA	45.2% peaks	47.2% peaks

B

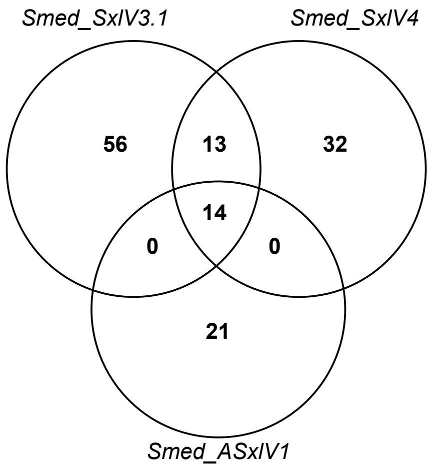

C

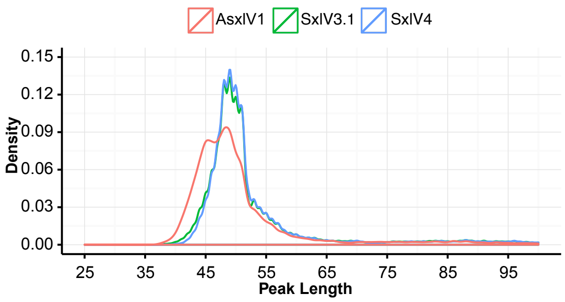

D

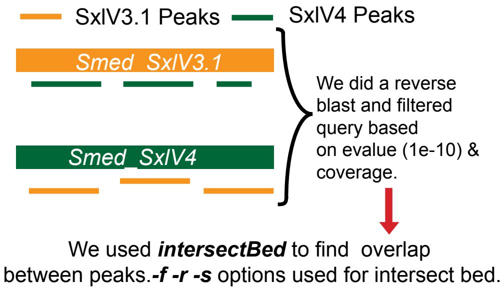

E

Peak overlap cutoff	*SxlV3.1 Peaks*	*SxlV4 Peaks*
>= 85%	18366 (**58%**)	23520 (**78.8%**)
>= 50%	19189 (**61%**)	24820 (**83%**)

Figure S15. Lift over of polyadenylation site coordinates from SmedSxl_V3.1 to SmedSxl_V4.0. A) The 3P reads derived from this study are individually aligned to SxlV4 & AsxlV1 genome and the same computational pipeline described earlier was used to identify polyadenylation sites. We identified 29836 & 11998 peaks from SxlV4 & AsxlV1 genome respectively. More than 45% of identified cleavage sites have AAUAAA as their PAS signal. **B**) Venn diagram depicting the overlap of identified major & minor/secondary signals from SxlV3.1, SxlV4 and AsxlV1 genome assemblies. 14 PAS are conserved across polyadenylation sites derived from three genome assemblies. **C**) The distribution of 3P-Peak lengths derived from three different genome assemblies. Polyadenylation site identified from SxlV3.1 & SxlV4 shows similar distribution. **D**) Schematic explaining methodology used to identify correspondence of polyadenylation peaks from SxlV3.1 to SxlV4 genome. We used reverse blast to identify genomic coordinates & then used bedtools (intersectbed) to calculate the overlap. **E**) Table highlighting the corresponding polyadenylation sites from SxlV3.1 and SxlV4 genome. Around 79% of the 3P-peaks determined from SmedSxlV4 genome overlapped with previously derived 3P-peak using SmedSxlV3.1 genome (with >=85% coverage overlap between cleavage sites).
