# Supplementary material for: Genome-Wide Analysis of Polyadenylation Events in Schmidtea mediterranea
Source: G3 (Bethesda). 2016 Aug 2;6(10):3035–48. doi: 10.1534/g3.116.031120 (PMC5068929; doi:10.1534/g3.116.031120)
Supplement: Supplemental Material [file supp_g3.116.031120_FigureS9.pdf]

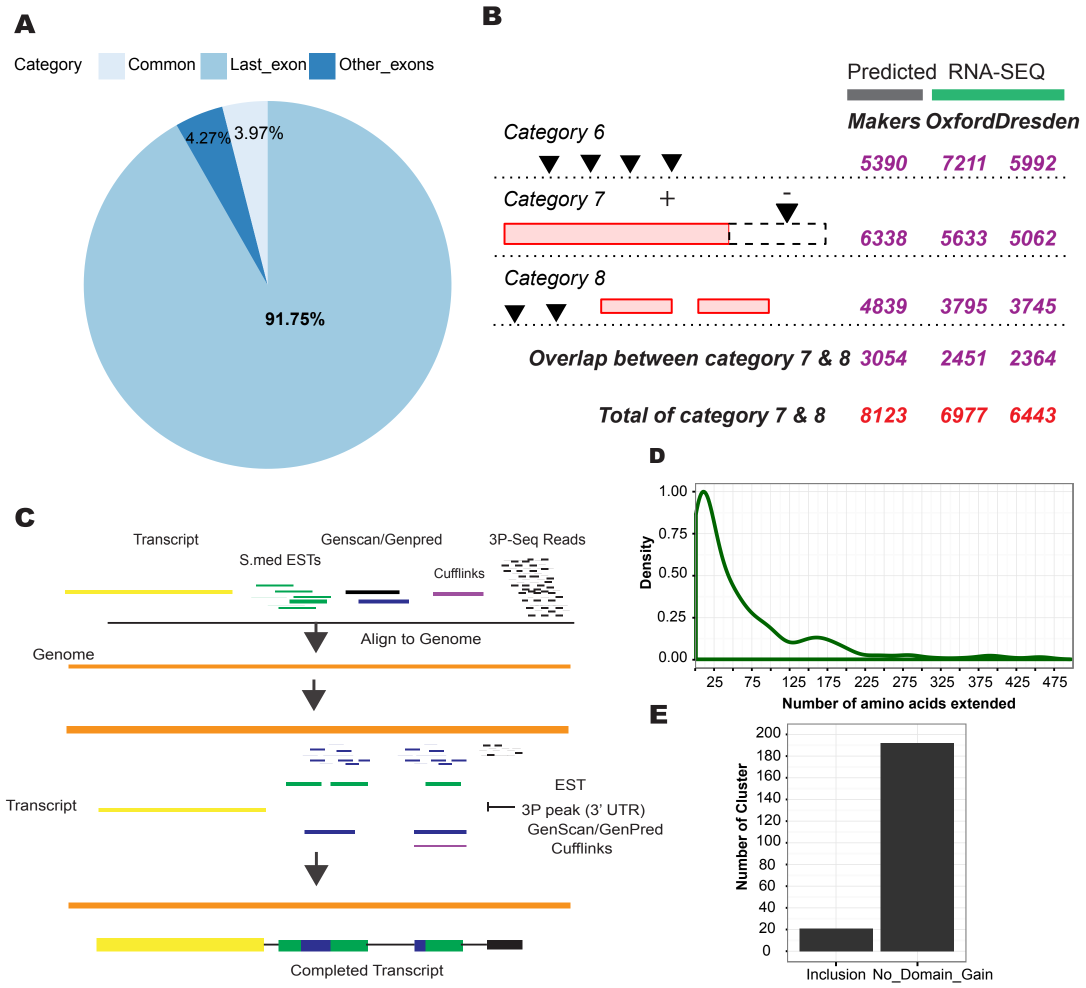

Figure S9. Completing gene models for planarian transcriptomes. A) The pie chart depicts the percentage distribution of polyadenylation sites across different exons. **B**) 3P-peaks that were present on the genomic contigs with no transcript annotation(Category 6). Number of polyadenylation sites that are in opposite orientation to the transcript (Category 7) & polyadenylation sites that cannot be associated with specific transcripts (Category 8). **C**) The schematic explains the computational workflow used to stitch the annotated 3'UTR to known transcripts. **D**) The density distribution of number of amino acids extended after stitching the known transcript model. **E**) The number of transcripts that had additional protein domain in the extended region of the transcript.
