# Supplementary material for: Genome-Wide Analysis of Polyadenylation Events in Schmidtea mediterranea
Source: G3 (Bethesda). 2016 Aug 2;6(10):3035–48. doi: 10.1534/g3.116.031120 (PMC5068929; doi:10.1534/g3.116.031120)
Supplement: Supplemental Material [file supp_g3.116.031120_FigureS10.pdf]

A

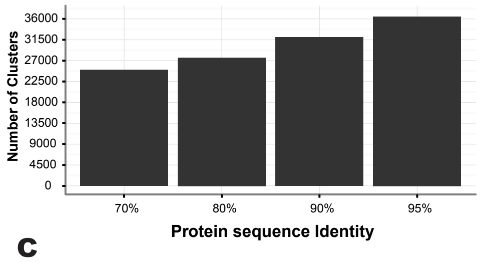

B

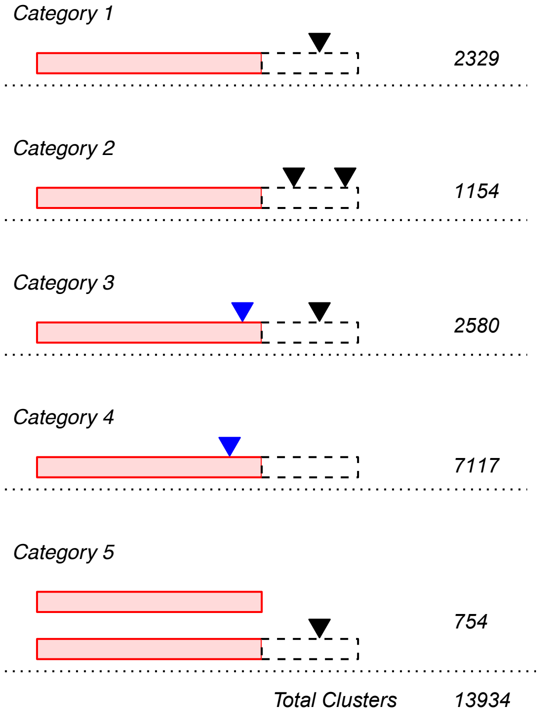

C

	Number of Clusters	Percentage
One Peak	8920	59.5
Two Peak	3550	25.5
Three Peak	1322	9.5
>=4 Peaks	772	5.5

D

	Number of Clusters
Annotated	7117
With Evidence	2647
No Evidence	4170

E

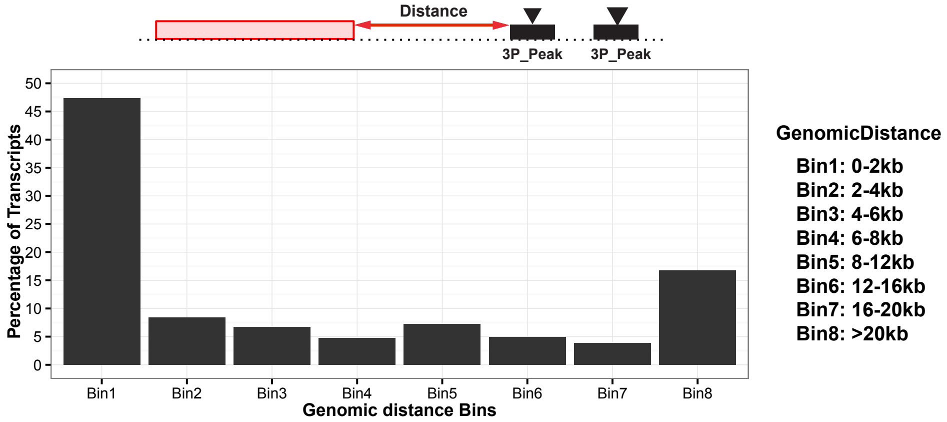

Figure S10. 3'UTR association with the non-redundant planarian transcriptome. A) The barplot represents the number of clusters obtained from CD-HIT using different % sequence identity thresholds. **B)** The distribution of derived gene clusters across different transcript association categories. **C)** The number of transcripts (gene clusters) associated with different polyadenylation sites. Around 40% of transcripts associate with more than one polyadenylation sites. **D)** **N**umber of gene clusters that could be extended further based upon the 3P-Peaks associated with them. **E)** Genomic distance of nearest polyadenylation site found from the known transcript models are binned. Around 48% of gene models are associated with polyadenylation/cleavage site within 2kb downstream.
