# Supplementary material for: Genome-Wide Analysis of Polyadenylation Events in Schmidtea mediterranea
Source: G3 (Bethesda). 2016 Aug 2;6(10):3035–48. doi: 10.1534/g3.116.031120 (PMC5068929; doi:10.1534/g3.116.031120)
Supplement: Supplemental Material [file supp_g3.116.031120_FigureS11.pdf]

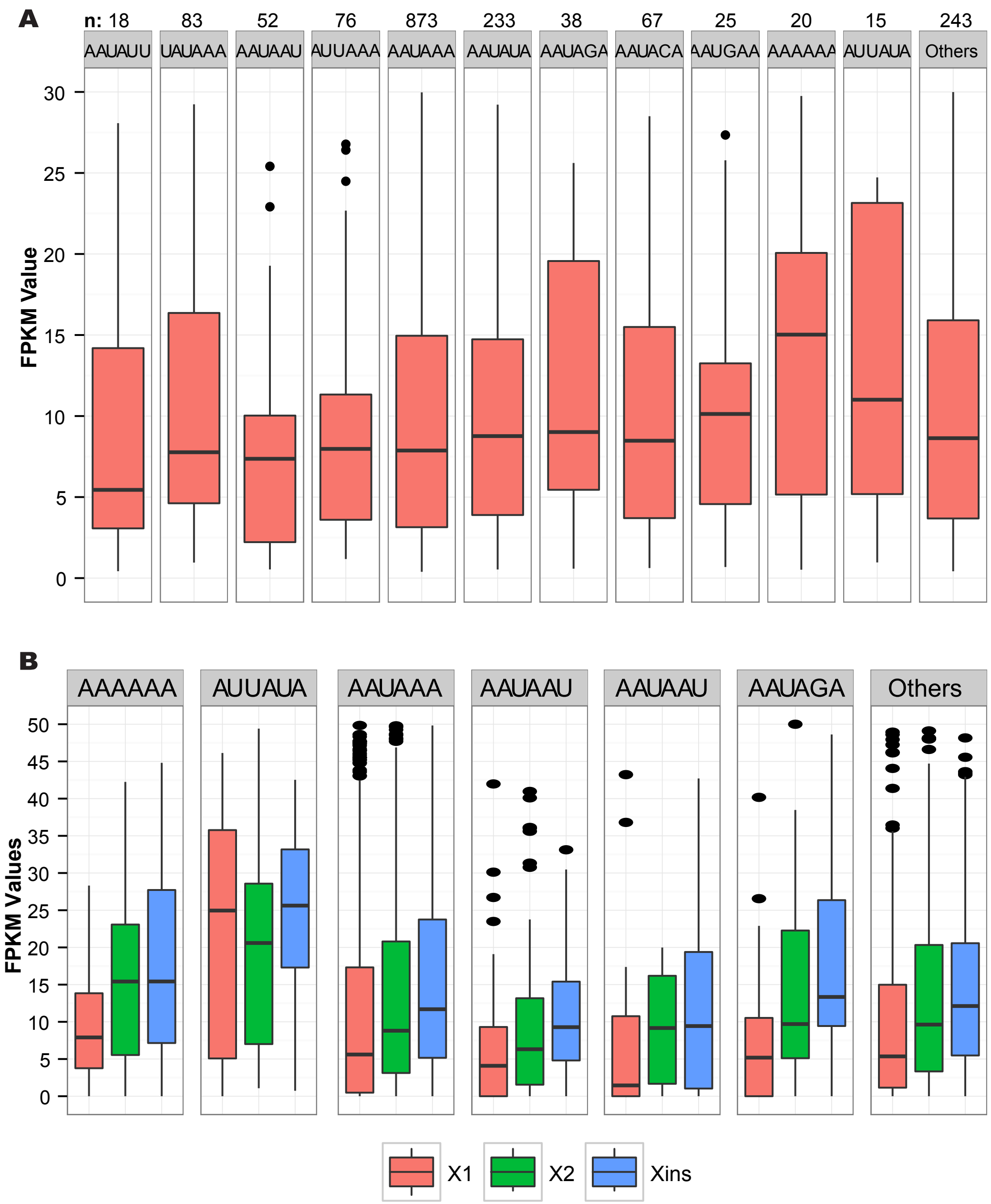

Figure S11. Correlation of PAS with transcript level (FPKM). A) The box plot depicts FPKM distribution of transcripts having top 12 polyadenylation signals. Transcripts with one annotated 3'UTR is considered for analysis. **B**) Transcripts with particular PAS, which showed distinct FPKM distribution profile are chosen and looked for cell-type specific FPKM distribution.
