# Supplementary material for: Genome-Wide Analysis of Polyadenylation Events in Schmidtea mediterranea
Source: G3 (Bethesda). 2016 Aug 2;6(10):3035–48. doi: 10.1534/g3.116.031120 (PMC5068929; doi:10.1534/g3.116.031120)
Supplement: Supplemental Material [file supp_g3.116.031120_FigureS12.pdf]

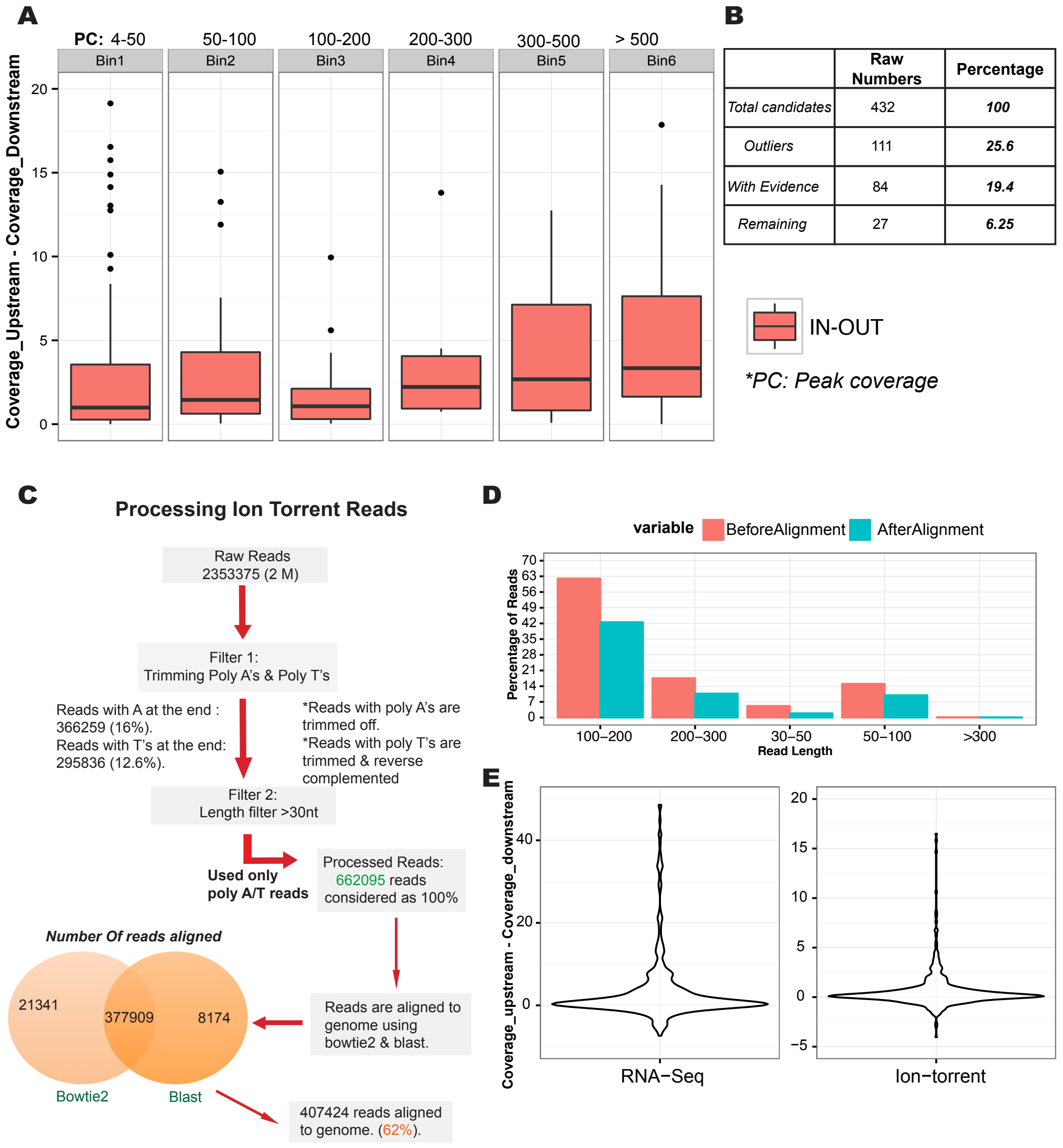

Figure S12. Validation of 3'UTRs using RNA-Seq data. A) RNASeq coverage for upstream region (from transcript end to polyadenylation site start) and downstream region (after polyadenylation site) across different bins of polyadenylation site coverage (PC). The difference in the RNA-Seq coverage values in the upstream and the downstream region increases with the increase in PC. **B)** Table depicting raw number and percentage of transcripts used in validation of 3P-Seq using RNA-Seq data analysis. **C)** The schematic describing the different steps of pre-processing for reads obtained from Ion torrent platform. **D)** The binned-frequency of the Ion-torrent read lengths. More than 80% of reads have > 100nts. **E)** A violin plot depicting the difference in the coverage between the upstream and downstream region derived from RNA-Seq and Ion-Torrent reads.
