# Supplementary material for: Genome-Wide Analysis of Polyadenylation Events in Schmidtea mediterranea
Source: G3 (Bethesda). 2016 Aug 2;6(10):3035–48. doi: 10.1534/g3.116.031120 (PMC5068929; doi:10.1534/g3.116.031120)
Supplement: Supplemental Material [file supp_g3.116.031120_FigureS13.pdf]

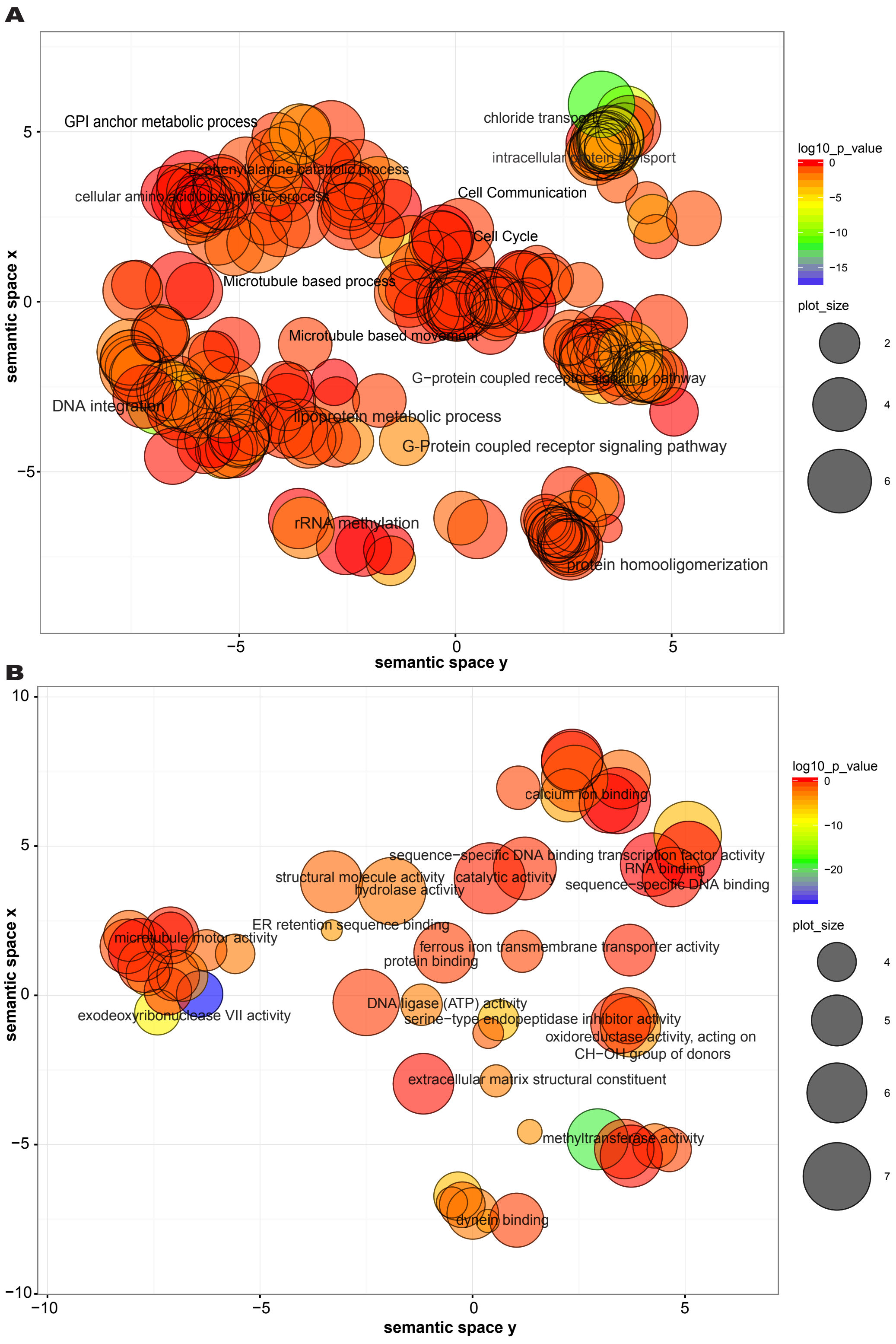

Figure S13. GO enrichment for two-3P peak & coding region polyadenylation (crApA) candidates. **A**) Scatterplot derived from REVIGO analysis depicting the clusters of GO terms (Biological Process) associated with the transcripts having multiple polyadenylation sites. Each circle represents a GO-term, the color reflects the significance of enrichment and the size denotes the abundance of that particular GO-term in the background uniprot dataset. **B**)

Scatterplot derived from REVIGO analysis depicting the clusters of GO terms associated with polyadenylation event that could lead to putative loss of protein domains.
