# Supplementary material for: Genome-Wide Analysis of Polyadenylation Events in Schmidtea mediterranea
Source: G3 (Bethesda). 2016 Aug 2;6(10):3035–48. doi: 10.1534/g3.116.031120 (PMC5068929; doi:10.1534/g3.116.031120)
Supplement: Supplemental Material [file supp_g3.116.031120_FigureS14.pdf]

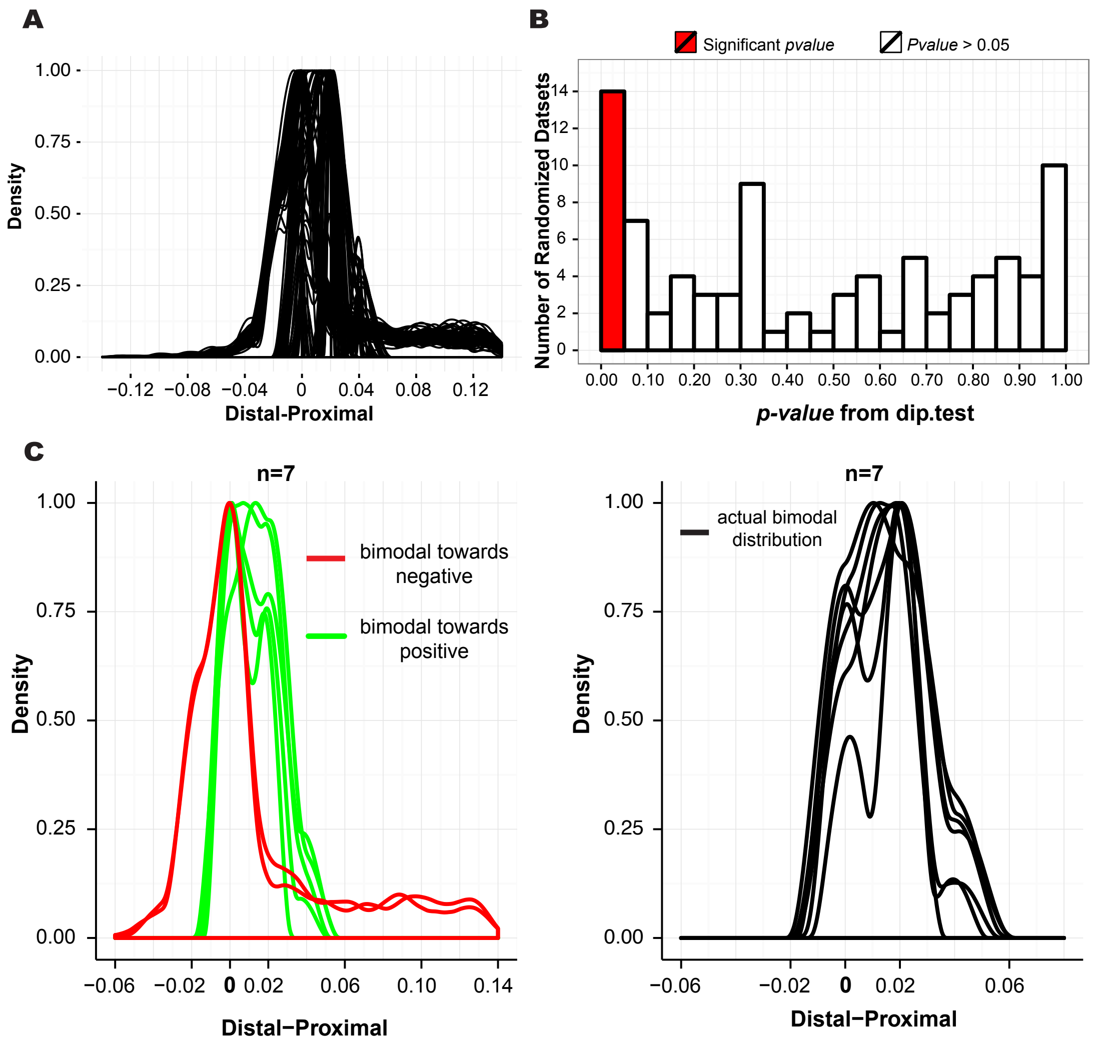

Figure S14. Control dataset for miRNA binding site distribution across two 3P peak candidates. A) Density distribution of difference between number of miRNA binding site at proximal & distal 3P-peak of randomized control dataset (explained in methods). **B**) We performed Hartigans' dip test for multi-modality of the randomized 100 datasets to see how many of the randomized dataset follow multi-modal distribution. Histogram of binned *P-values* from Hartigans' dip test is plotted. **C**) This shows that 14 out of 101 randomized dataset show significant *P-value* from dip test (suggesting bimodal distribution). Only seven (right panel) of the 14 datasets had the same bimodal profile as seen in actual dataset.
