# Supplementary material for: Genome-Wide Analysis of Polyadenylation Events in Schmidtea mediterranea
Source: G3 (Bethesda). 2016 Aug 2;6(10):3035–48. doi: 10.1534/g3.116.031120 (PMC5068929; doi:10.1534/g3.116.031120)
Supplement: Supplemental Material [file supp_g3.116.031120_FigureS15.pdf]

A

	Smed_SxlV4	Smed_ASxlV1
Total Reads	90416419	83937226
Aligned Reads	33841718	31539047
3P Tags	15983576	16557733
3P Peaks	29836	11998
Enriched Signal	607	409
Minor Signals	58	34
Peaks with AAUAAA	45.2% peaks	47.2% peaks

B

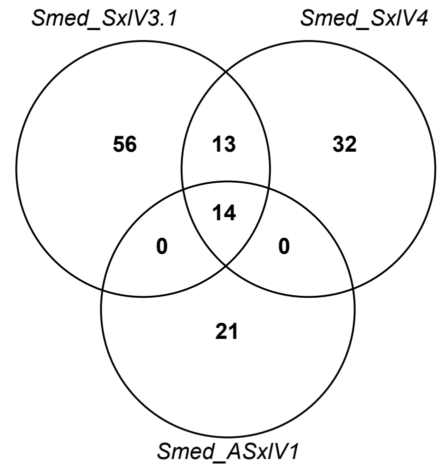

C

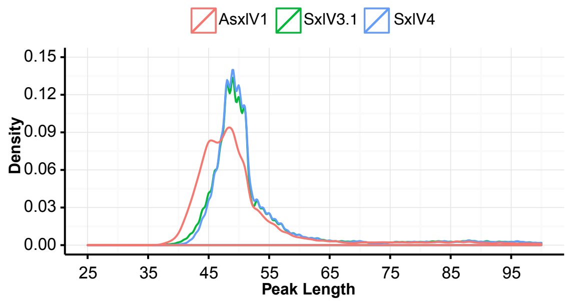

D

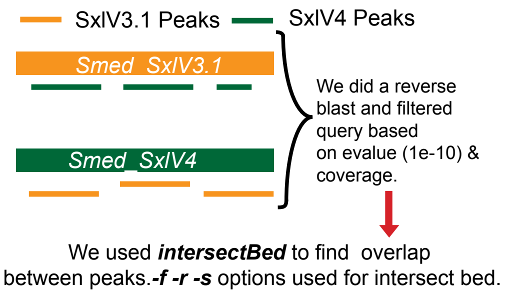

E

Peak overlap cutoff	*SxlV3.1 Peaks*	*SxlV4 Peaks*
>= 85%	18366 (**58%**)	23520 (**78.8%**)
>= 50%	19189 (**61%**)	24820 (**83%**)

Figure S15. Lift over of polyadenylation site coordinates from SmedSxl_V3.1 to SmedSxl_V4.0. A) The 3P reads derived from this study are individually aligned to SxlV4 & AsxlV1 genome and the same computational pipeline described earlier was used to identify polyadenylation sites. We identified 29836 & 11998 peaks from SxlV4 & AsxlV1 genome respectively. More than 45% of identified cleavage sites have AAUAAA as their PAS signal. **B**) Venn diagram depicting the overlap of identified major & minor/secondary signals from SxlV3.1, SxlV4 and AsxlV1 genome assemblies. 14 PAS are conserved across polyadenylation sites derived from three genome assemblies. **C**) The distribution of 3P-Peak lengths derived from three different genome assemblies. Polyadenylation site identified from SxlV3.1 & SxlV4 shows similar distribution. **D**) Schematic explaining methodology used to identify correspondence of polyadenylation peaks from SxlV3.1 to SxlV4 genome. We used reverse blast to identify genomic coordinates & then used bedtools (intersectbed) to calculate the overlap. **E**) Table highlighting the corresponding polyadenylation sites from SxlV3.1 and SxlV4 genome. Around 79% of the 3P-peaks determined from SmedSxlV4 genome overlapped with previously derived 3P-peak using SmedSxlV3.1 genome (with >=85% coverage overlap between cleavage sites).
