# Supplementary material for: Genome-Wide Analysis of Polyadenylation Events in Schmidtea mediterranea
Source: G3 (Bethesda). 2016 Aug 2;6(10):3035–48. doi: 10.1534/g3.116.031120 (PMC5068929; doi:10.1534/g3.116.031120)
Supplement: Supplemental Material [file supp_g3.116.031120_FigureS1.pdf]

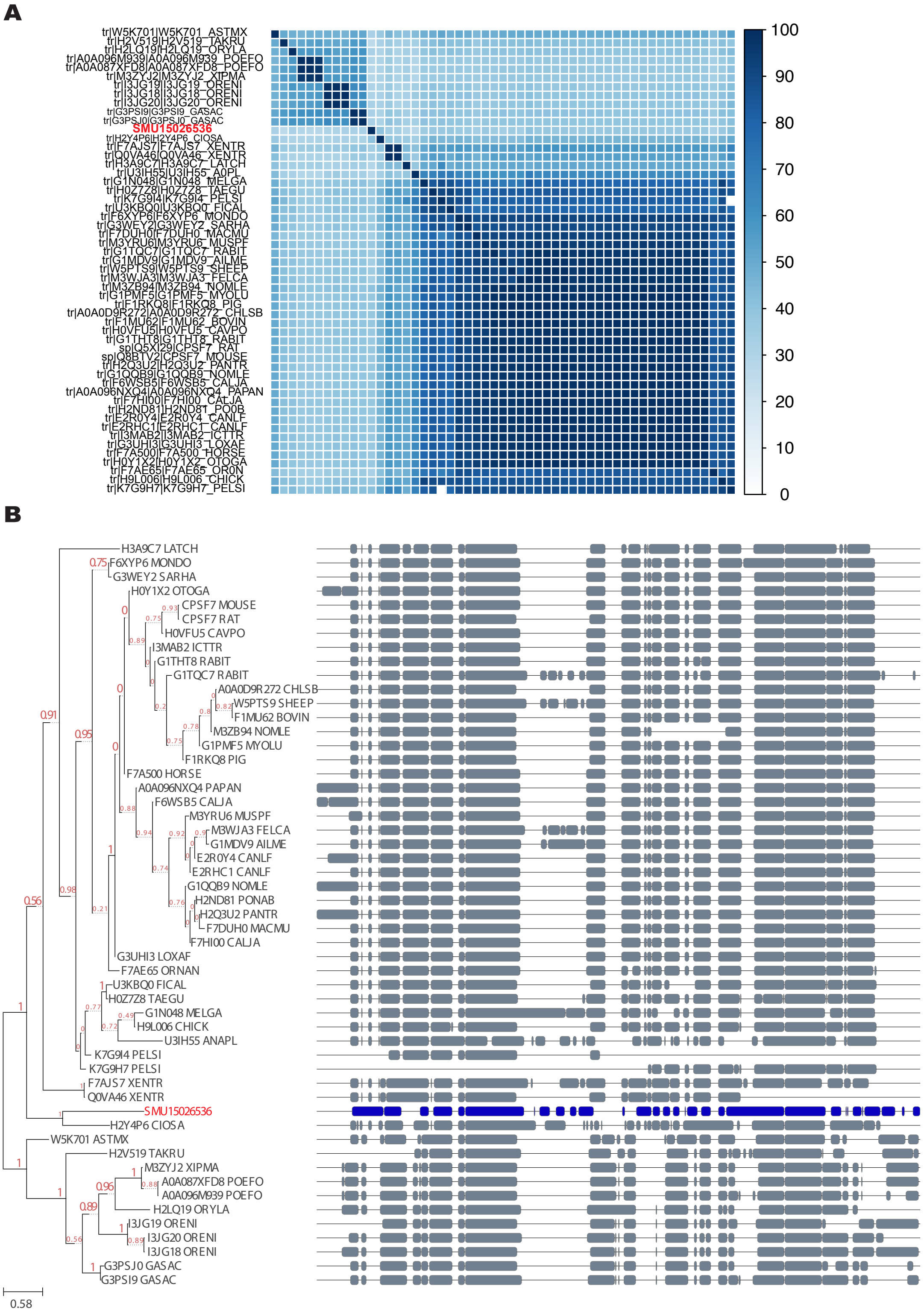

Figure S1. CFIm59 (CPSF7) in *Schmidtea mediterranea*. A) The percentage sequence identity matrix derived from *clustal omega* for CFIm59, a component of cleavage factor-Im59 complex. **B**) Single gene tree plotted for planarian CFIm59 with other organisms CFIm59 using ***ete-build***. Left panel of the plot shows phylogenetic tree constructed based on alignment blocks shown on right. Planarian CPSF7 sequence stands out as an outlier in

comparison to other CFIm59 proteins, suggesting the high variation in the sequence that could exist for this protein within planaria.
