# Supplementary material for: Genome-Wide Analysis of Polyadenylation Events in Schmidtea mediterranea
Source: G3 (Bethesda). 2016 Aug 2;6(10):3035–48. doi: 10.1534/g3.116.031120 (PMC5068929; doi:10.1534/g3.116.031120)
Supplement: Supplemental Material [file supp_g3.116.031120_FigureS2.pdf]

A

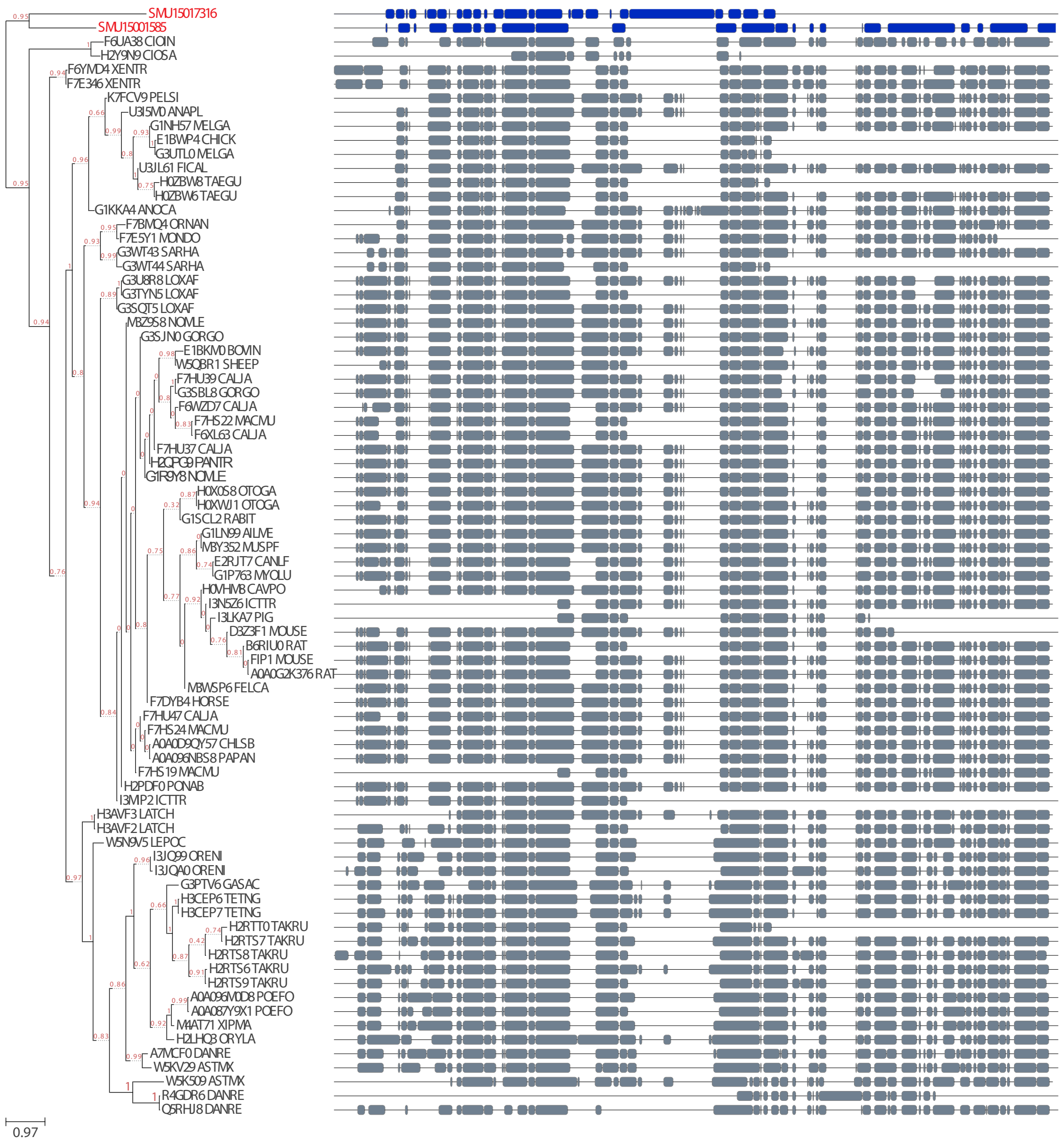

Figure S2. Gene tree for putative *Schmidtea* Fip1. A) Single gene based phylogeny plotted for planarian Fip1, a subunit from cleavage and polyadenylation machinery along with Fip1 gene from other organisms using ***ete-build***. Planarian Fip1 sequence clusters outside all known Fip1 genes and has very poor % sequence identity with other known Fip1 sequences (data not shown). This suggests that currently derived planarian Fip1 sequence is highly variable in comparison to Fip1 from other organisms.
