# Supplementary material for: Genome-Wide Analysis of Polyadenylation Events in Schmidtea mediterranea
Source: G3 (Bethesda). 2016 Aug 2;6(10):3035–48. doi: 10.1534/g3.116.031120 (PMC5068929; doi:10.1534/g3.116.031120)
Supplement: Supplemental Material [file supp_g3.116.031120_FigureS3.pdf]

A

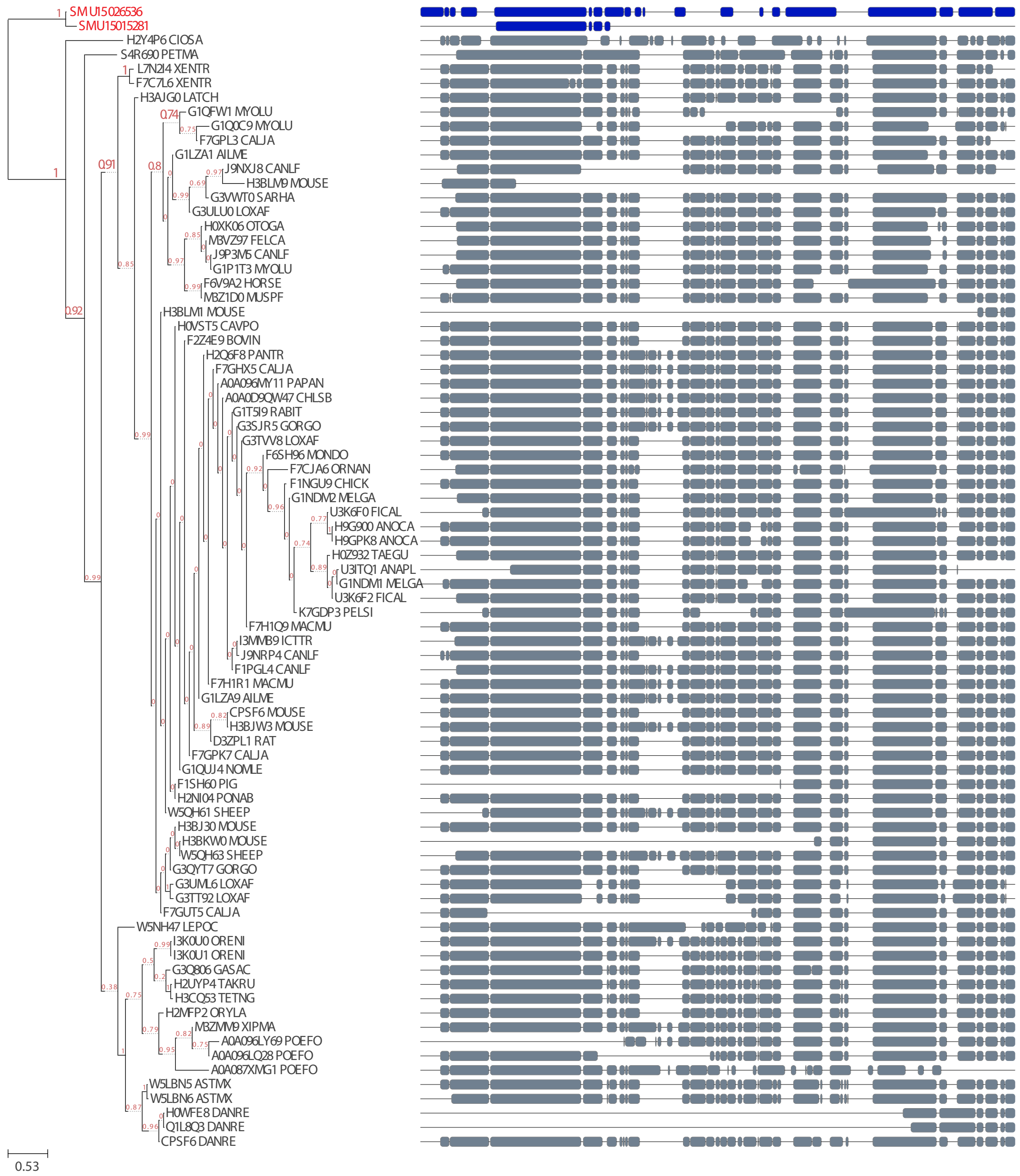

Figure S3. Gene tree for *Schmidtea* CFIm68 (CPSF6). A) Single gene based phylogeny plotted for planarian CPSF6, a component of cleavage factor-Im68 complex with CPSF6 gene from other organisms using ***ete-build***. Planarian CPSF6 sequence clusters outside all known CPSF6 genes and has very poor % sequence identity with other known CPSF6 sequences (data not shown). This suggests that currently predicted planarian CPSF6 from recent genome has high sequence variation in comparison with CPSF6 from other organisms.
