# Supplementary figures and images for: Genome-Wide Analysis of Polyadenylation Events in Schmidtea mediterranea
Source: G3 (Bethesda). 2016 Aug 2;6(10):3035–48. doi: 10.1534/g3.116.031120 (PMC5068929; doi:10.1534/g3.116.031120)

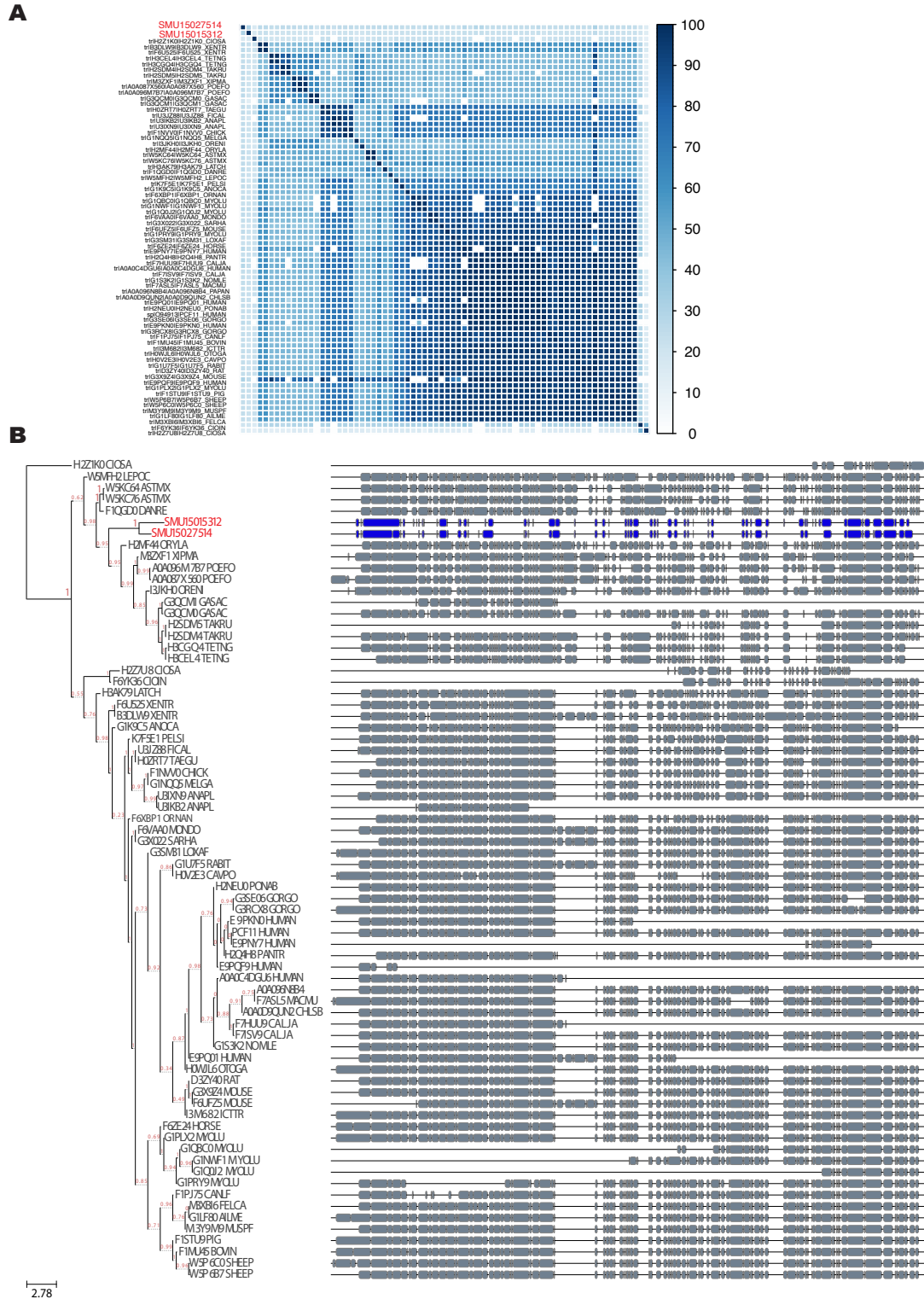

Supplement: Supplemental Material [file supp_g3.116.031120_FigureS4.pdf]
