# Supplementary material for: Genome-Wide Analysis of Polyadenylation Events in Schmidtea mediterranea
Source: G3 (Bethesda). 2016 Aug 2;6(10):3035–48. doi: 10.1534/g3.116.031120 (PMC5068929; doi:10.1534/g3.116.031120)
Supplement: Supplemental Material [file supp_g3.116.031120_FigureS5.pdf]

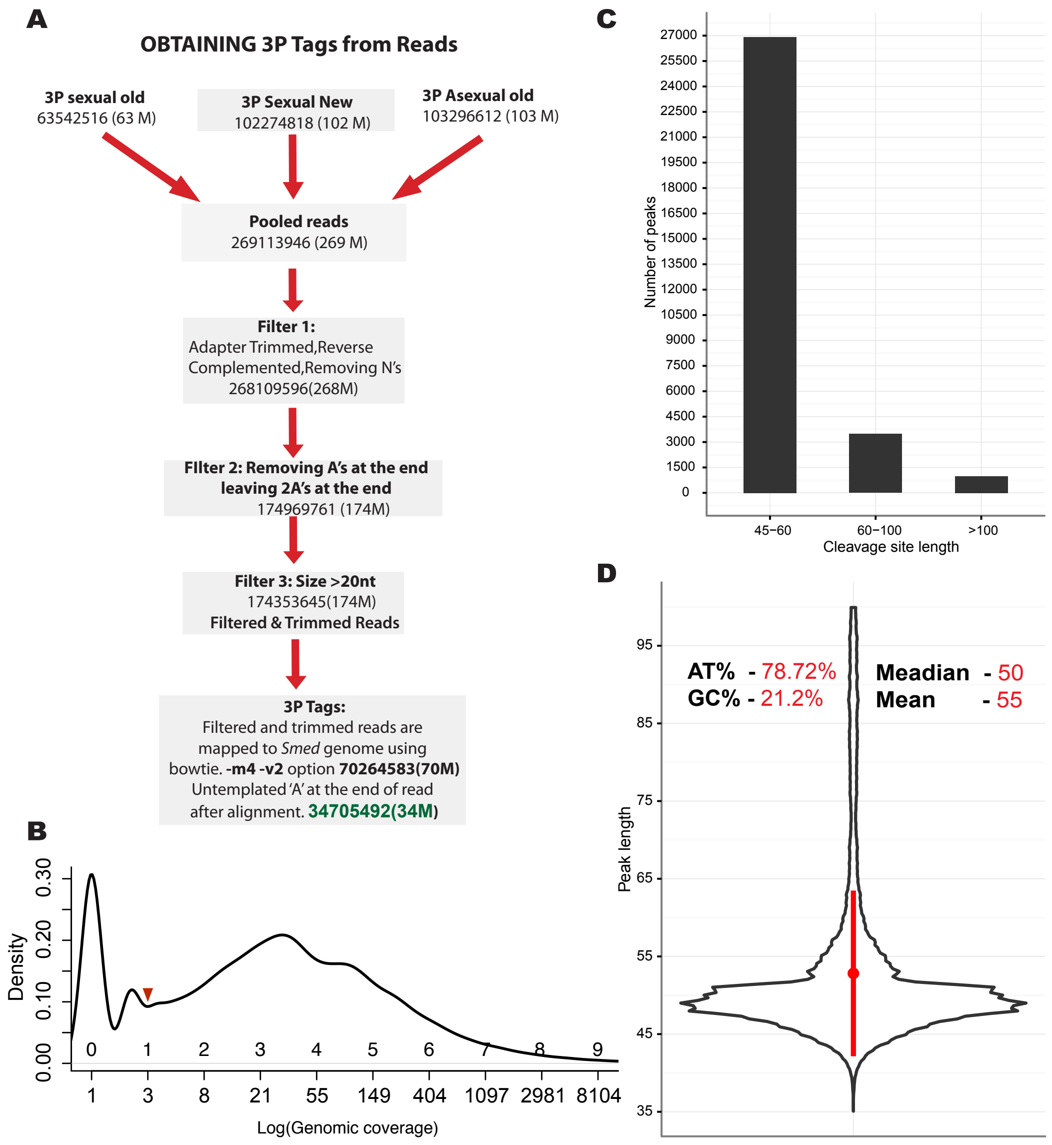

Figure S5. Identification of polyadenylation/cleavage sites from SmedSxl.V3.1 genome. **A**) The computational pipeline to obtain 3P tags from 3P-seq reads. The workflow explains all the pre-processing steps to obtain 3P-tags. **B**) Density distribution of reads per locus indicating the genomic coverage of 3P-tags. The x-axis denotes the genomic coverage in normal (below) & log scale (above), whereas the y-axis represents the density of number of locus. **C)** Binned histogram representing the length of 3P-peaks. Majority of the 3P-Peaks are 45-60nts in length. **D**) Violin plot representing the length distribution of identified cleavage/polyadenylation peaks (3P-Peaks) from our computational pipeline. The mean and median of the distribution is 50 nts & 55nts respectively. As expected, identified cleavage sites are AT rich suggesting that these are from 3' end of the transcript.
