# Supplementary material for: Genome-Wide Analysis of Polyadenylation Events in Schmidtea mediterranea
Source: G3 (Bethesda). 2016 Aug 2;6(10):3035–48. doi: 10.1534/g3.116.031120 (PMC5068929; doi:10.1534/g3.116.031120)
Supplement: Supplemental Material [file supp_g3.116.031120_FigureS6.pdf]

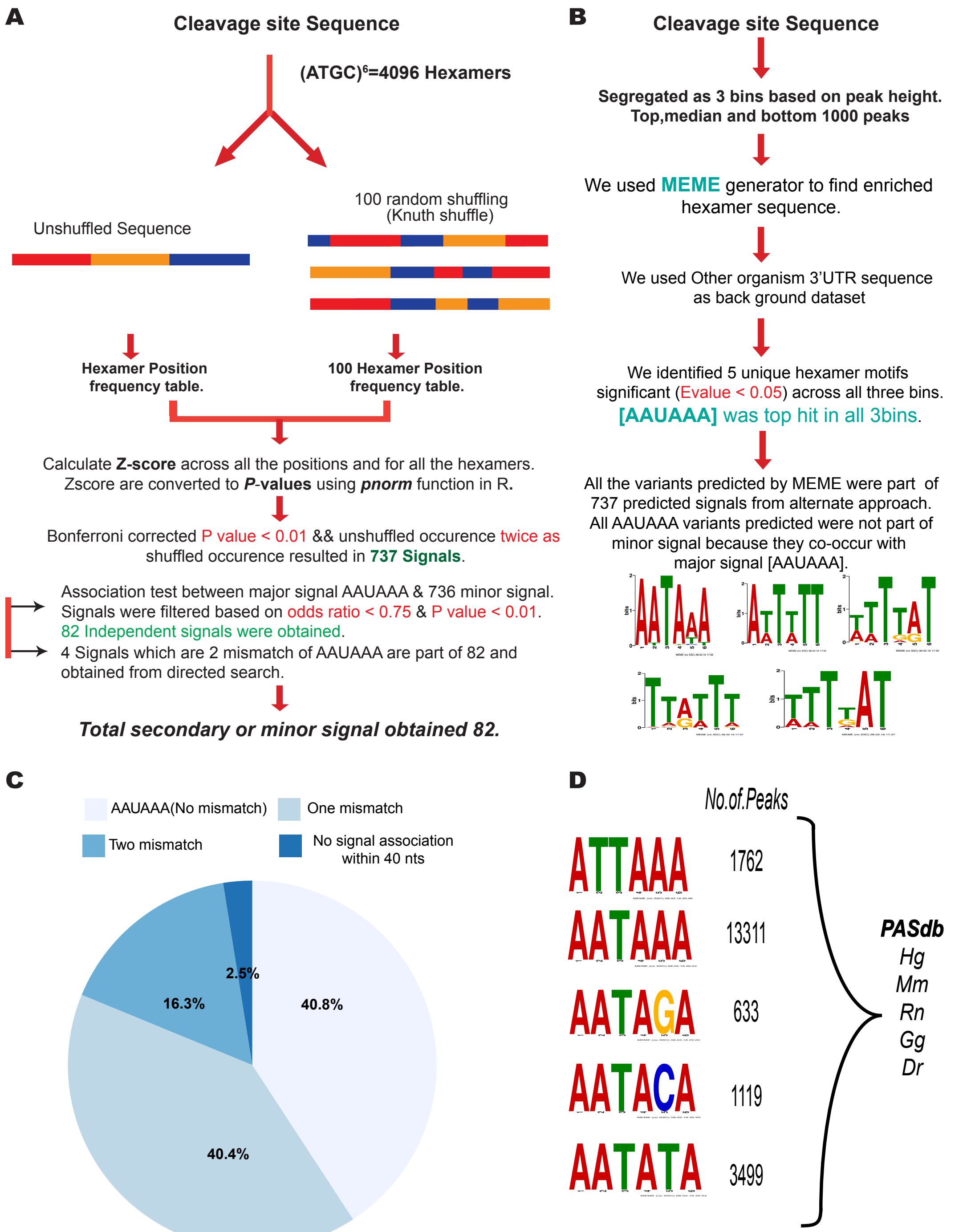

Figure S6. Identification of conserved PolyA signal (PAS). **A**) Schematic describing the computational pipeline to identify PAS signal. Identification of major and minor/secondary signal by fisher's exact test & directed PAS search. **B**) Alternate pipeline used to identify PAS from identified cleavage site based on peak height. **C**) Association of PAS to identified cleavage sites. Venn diagram depicting the percentage of identified cleavage sites that have AAUAAA or single nucleotide variant of AAUAAA at 10-40nt upstream of cleavage sites. **D**) Overlap of the identified PAS from *Schmidtea* with PolyA signal database.
