# Supplementary material for: Genome-Wide Analysis of Polyadenylation Events in Schmidtea mediterranea
Source: G3 (Bethesda). 2016 Aug 2;6(10):3035–48. doi: 10.1534/g3.116.031120 (PMC5068929; doi:10.1534/g3.116.031120)
Supplement: Supplemental Material [file supp_g3.116.031120_FigureS7.pdf]

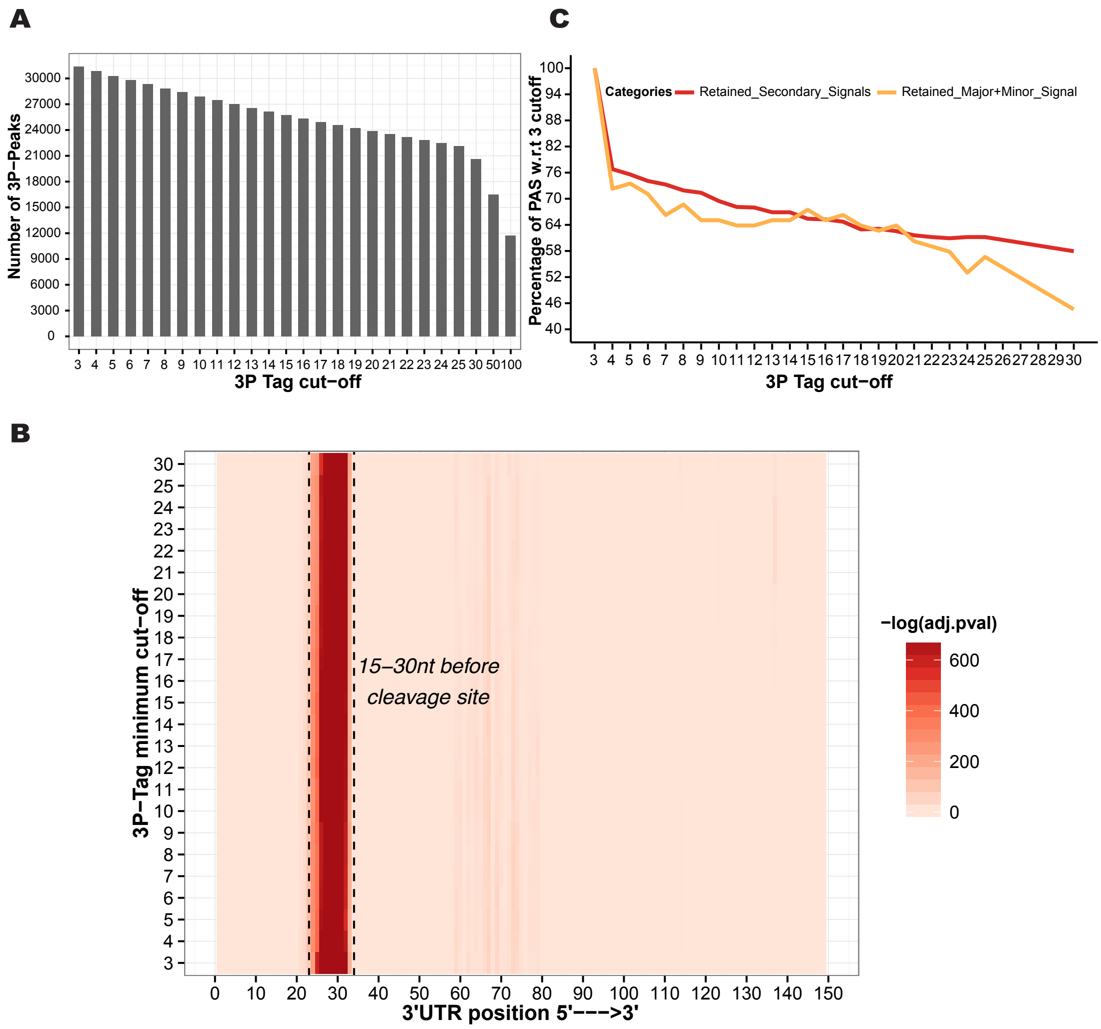

Figure S7. Retained PolyA signals (PAS) in different 3P-Tags cutoff. A) Bar plot depicting total number of identified cleavage sites (3P-Peaks) at different tag cut-off. **B**) Adjusted *Pvalue* obtained from hexamer enrichment analysis (as described before) for the major signal AAUAAA across 3P-peaks obtained from different 3P tag cutoff. **C**) Overlap percentage of enriched PAS and major+minor signal obtained from three 3P tag cutoff used in this study with other 3P tag cutoffs (4-30).
