# Supplementary material for: Genome-Wide Analysis of Polyadenylation Events in Schmidtea mediterranea
Source: G3 (Bethesda). 2016 Aug 2;6(10):3035–48. doi: 10.1534/g3.116.031120 (PMC5068929; doi:10.1534/g3.116.031120)
Supplement: Supplemental Material [file supp_g3.116.031120_FigureS8.pdf]

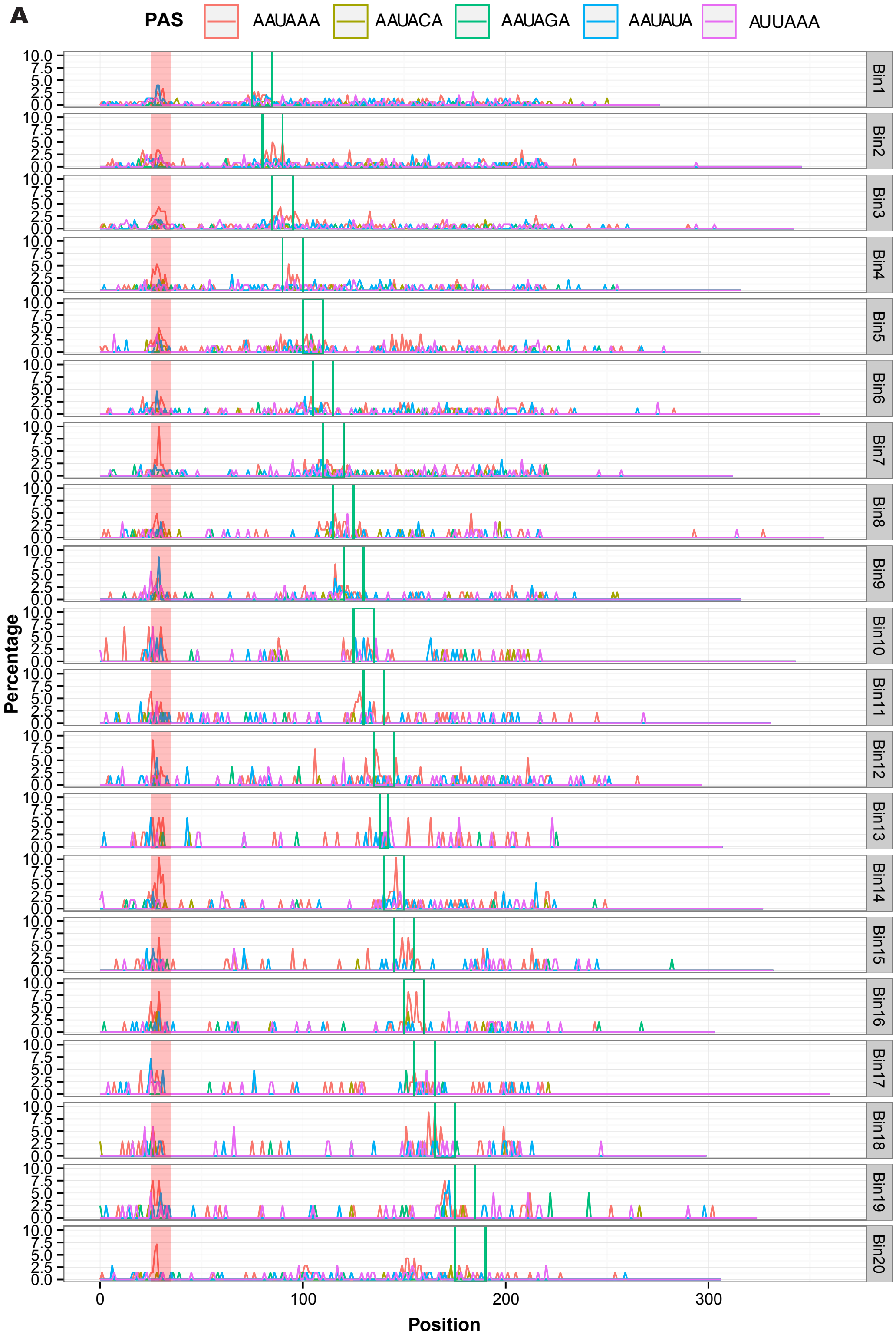
A
PAS
AAUAAA
AAUACA
AAUAGA
AAUAUA
AUUAAA
Percentage
Position
0
100
200
300
10.0
7.5
5.0
2.5
0.0
Bin1
Bin2
Bin3
Bin4
Bin5
Bin6
Bin7
Bin8
Bin9
Bin10
Bin11
Bin12
Bin13
Bin14
Bin15
Bin16
Bin17
Bin18
Bin19
Bin20

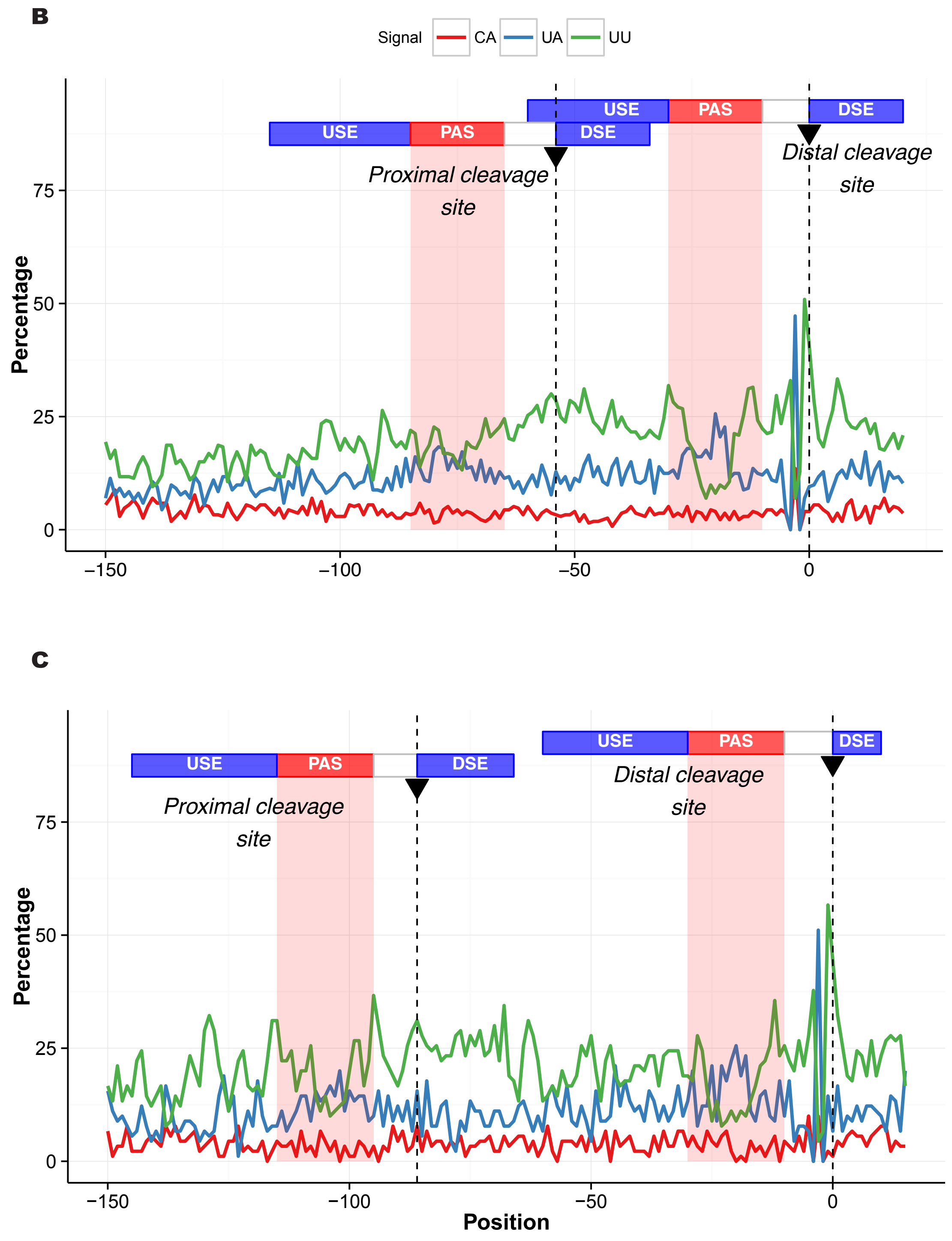

Figure S8. Distribution of PolyA signals for 3P-peaks that are in close proximity. A) Polyadenylation sites that are in close proximity to each other in genome (within 100nt) are binned with 5nt interval. We scanned for the presence of five enriched PAS signal in the region spanning 100nts on either side of the two-cleavage site that are close to each other. We observed that polyadenylation sites that are as close as 5nt (bin1) have distinct PAS associated at the distance of 10-30nt upstream suggesting these are independent polyadenylation sites. Highlighted region shows PAS signal in proximal 3P-Peak and boxed

region shows PAS signal in distal 3P-Peak. **B**) A line-plot showing the dinucleotide profile(CA, UU, UA) of all the 3P-peaks that are separated < 10nts. The 0-position on the x-axis denotes the cleavage site (annotated with dotted line and arrow). The upstream elements (USE) and the downstream elements (DSE), along with the polyadenylation signal (PAS) have been separately highlighted in blue and red respectively. **C**) A line plot similar to (**B**) showing dinucleotide profile of all the 3P peaks that are separated by 30-35nts.
